# Approaching disorder-tolerant semiconducting polymers

**DOI:** 10.1038/s41467-021-26043-y

**Published:** 2021-09-29

**Authors:** Xinwen Yan, Miao Xiong, Xin-Yu Deng, Kai-Kai Liu, Jia-Tong Li, Xue-Qing Wang, Song Zhang, Nathaniel Prine, Zhuoqiong Zhang, Wanying Huang, Yishan Wang, Jie-Yu Wang, Xiaodan Gu, Shu Kong So, Jia Zhu, Ting Lei

**Affiliations:** 1 Key Laboratory of Polymer Chemistry and Physics of Ministry of Education, School of Materials Science and Engineering, Peking University, Beijing, 100871 China; 2 Beijing National Laboratory for Molecular Science, College of Chemistry and Molecular Engineering, Peking University, Beijing, 100871 China; 3 Key Laboratory of Catalysis and Energy Materials Chemistry of Ministry of Education & Hubei Key Laboratory of Catalysis and Materials Science, Hubei R&D Center of Hyperbranched Polymers Synthesis and Applications, South-Central University for Nationalities, Wuhan, 430074 China; 4 Center for Optoelectronic Materials and Devices, School of Polymer Science and Engineering, The University of Southern Mississippi, Hattiesburg, MS 39406 USA; 5 Department of Physics, Institute of Advanced Materials, Hong Kong Baptist University, Kowloon Tong, Hong Kong SAR, China; 6 Key Laboratory of Theoretical and Computational Photochemistry of Ministry of Education, College of Chemistry, Beijing Normal University, Beijing, 100875 China; 7 Laboratory of Theoretical and Computational Nanoscience, CAS Center for Excellence in Nanoscience, National Center for Nanoscience and Technology, Chinese Academy of Sciences, Beijing, 100190 China

**Keywords:** Conjugated polymers, Synthesis and processing, Polymers

## Abstract

Doping has been widely used to control the charge carrier concentration in organic semiconductors. However, in conjugated polymers, n-doping is often limited by the tradeoff between doping efficiency and charge carrier mobilities, since dopants often randomly distribute within polymers, leading to significant structural and energetic disorder. Here, we screen a large number of polymer building block combinations and explore the possibility of designing n-type conjugated polymers with good tolerance to dopant-induced disorder. We show that a carefully designed conjugated polymer with a single dominant planar backbone conformation, high torsional barrier at each dihedral angle, and zigzag backbone curvature is highly dopable and can tolerate dopant-induced disorder. With these features, the designed diketopyrrolopyrrole (DPP)-based polymer can be efficiently n-doped and exhibit high n-type electrical conductivities over 120 S cm^−1^, much higher than the reference polymers with similar chemical structures. This work provides a polymer design concept for highly dopable and highly conductive polymeric semiconductors.

## Abstract (web summary)

In conjugated polymers, n-doping is often limited by the tradeoff between doping efficiency and charge carrier mobilities, since dopants often randomly distribute within polymers, leading to significant structural and energetic disorder. Here, the authors screen a large number of polymer building block combinations and explore the possibility of designing n-type conjugated polymers with good tolerance to dopant-induced disorder.

## Introduction

Polymeric semiconductors have been intensively studied for next-generation flexible or stretchable optoelectronic devices because of their unique mechanical properties and good solution processability^1,2^. To tune the charge-carrier concentration, Fermi level, and electrical conductivity of conjugated polymers, molecular doping is widely used^3–7^. However, the introduced dopants tend to randomly distribute within polymer film and bring large structural and energetic disorder^8^. *P*-type dopants can be small-size Lewis acids (e.g., ferric chloride), small molecules (e.g., 2,3,5,6-tetrafluoro-7,7,8,8-tetracyanoquinodimethane), and polymers (e.g., poly(4-styrene sulfonate) (PSS)). These *p*-dopants are strong oxidants or acids, and can be incorporated into the polymer matrix without significantly disrupting the ordered molecular packings and thus leading to high *p*-type electrical conductivities^9^. High charge-carrier concentration over 10^21^ cm^−3^ and high electrical conductivities over 1000 S cm^−1^ have been achieved in *p*-doped polymers, such as poly(2,5-bis(3-hexadecylthiophen-2-yl)thieno[3,2-b]thiophene)^7^ and poly(3,4-ethylenedioxythiophene):PSS)^10^. In contrast, the commonly used *n*-type dopants usually have weak reducing ability and relatively large size to achieve good air stability^11^, resulting in a significant increase of disorder after doping. Therefore, the charge-transport properties of *n*-type polymers are strongly hindered by the undesirable doping-induced disorder. Only a few *n*-doped conjugated polymers are reported to achieve conductivities approaching or over 10 S cm^−1^ ^12–17^.

The low electrical conductivities of *n*-doped conjugated polymers are mainly restricted by low doping efficiency and dopant-induced structural and energetic disorder. Many efforts have been devoted to improving the *n*-doping efficiency of conjugated polymers through the introduction of electron-deficient building blocks in the past decades^18^. However, the synthesis of strong electron-deficient building blocks is challenging^17^, as organic molecules with the lowest unoccupied molecular orbital (LUMO) energy levels lower than −4.4 eV might become unstable^19^. Furthermore, the *n*-doping efficiency is also limited by the solid-state miscibility between polymer and *n*-dopants, as efficient charge transfer between polymer and dopant requires close contact^20,21^. To enhance the solid-state miscibility, several polymer design strategies have been developed. For example, polar ethylene glycol side chains were employed to replace conventional alkyl side chains to improve the *n*-doping efficiency^22^. Twisted and “kinked” donor moieties were used to decrease the crystallinity of the conjugated polymers, constructing percolation space for dopants to improve doping efficiency^23,24^. However, these strategies always result in significant structural and energetic disorder, bringing about declined charge-carrier mobilities and poor electrical conductivities^22–24^.

**Fig. 1:**
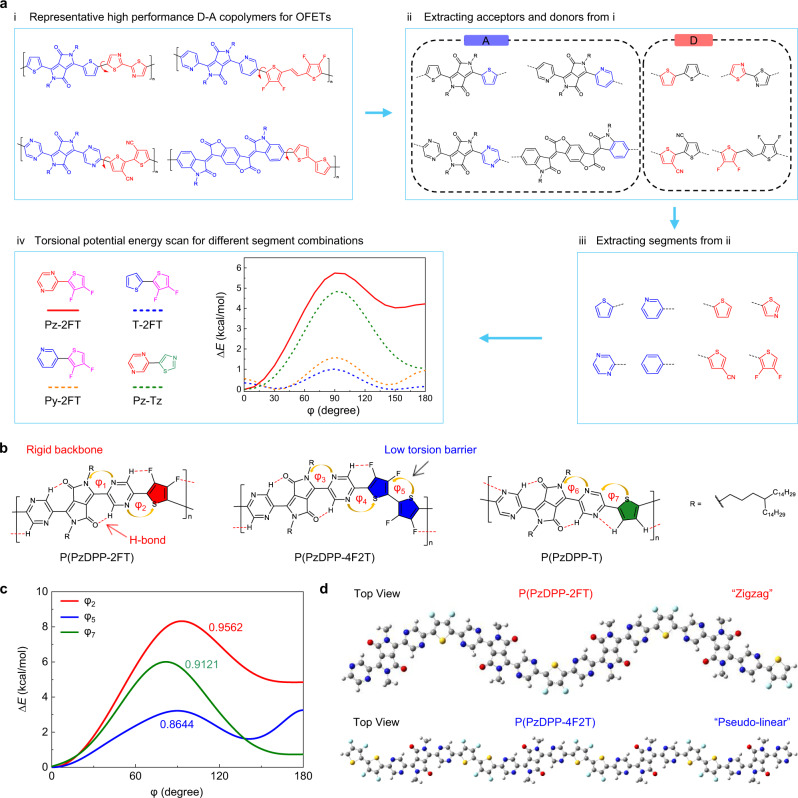
Computer-aided polymer design. **a** Computer-aided polymer building block screening process: (i) Collecting representative high-performance D–A copolymers used for OFETs; (ii) extracting building blocks; (iii) further extracting the segments for simplifying the screening process; (iv) PES of the dihedral angles of different segment combinations. (**b**) Chemical structures of P(PzDPP-2FT), P(PzDPP-4F2T), and P(PzDPP-T). **c** PES results of the torsional angles *φ*_2_, *φ*_5_, and *φ*_7_ in these three polymers (ωB97X-D/6-311G(d,p)). The energy is relative to the minimal energy conformer. Planarity indices 〈cos^2^*φ*〉 of these torsional angles were calculated based on PES results. **d** The DFT-optimized geometries of the tetramers of P(PzDPP-2FT) and P(PzDPP-4F2T). Long alkyl chains were replaced with methyl groups.

Here we explore the design of *n*-type conjugated polymers with high tolerance to dopant-induced disorder. With a computer-aided molecular design approach, we screened currently available polymer building blocks and their possible combinations. We found that the pyrazine (Pz) and 3,4-difluorothiophene (2FT) combination exhibited the highest torsional barrier, best planarity, and single dominant conformation, providing a highly rigid polymer backbone (Fig. 1). Therefore, we synthesized the polymer P(PzDPP-2FT) (Fig. 1b) with a zigzag backbone and relatively large Urbach energy, which are usually thought to result in low charge-carrier mobilities^25,26^. However, P(PzDPP-2FT) exhibits high electron mobility due to its narrow conformation distribution, low energetic disorder, and strong interchain interactions. The zigzag backbone provides the polymer with a dopant-binding cavity compared with polymers with linear backbones and enhances doping efficiency. High mobility and enhanced doping efficiency synergistically contribute to a high *n*-type electrical conductivity over 120 S cm^−1^.

## Results

### Computer-aided polymer design

Previous studies have suggested that to achieve efficient charge transport in conjugated polymers, intrachain charge transport and interchain short-range contacts are critical^27,28^. As the intrachain charge-transport behavior is closely associated with the conformational distribution of the polymer backbone, carefully tuning the torsion-angle distribution between the polymer building blocks from the level of molecular design is crucial for maintaining low conformation disorder^29^. Therefore, we propose that there are three critical rules of designing conjugated polymers to enhance the tolerance to dopant-induced disorder: (1) high coplanarity to obtain good intrachain and interchain charge transport, which can be quantified through a planarity index 〈cos^2^*φ*〉^30^; (2) single preferential planar backbone conformation; and (3) high and steep torsion barriers that are essential to restrain the polymer backbone to maintain a coplanar conformation and enhance the tolerance to dopant-induced disorders. Although Rule (1) (〈cos^2^*φ*〉 value) has considered all torsional conformations and their relative contribution to the overall structural disorder, it is still not enough to screen the desired polymers with minimal dihedral angle distributions. Therefore, we introduced Rules (2) and (3) (see Supplementary Information for more details).

Based on these concepts, we employed density functional theory (DFT) calculations to screen currently available high-performance polymer building blocks. We hope to find a combination with the best planarity, highest torsional barrier, and single dominant conformation (Fig. 1a and more details in Supplementary Information Section 2). After extracting donor and acceptor segments from various representative donor–acceptor (D-A) polymers, relaxed potential energy scans (PESs) were performed at the dihedral angles of adjacent units. After comparing the performance of several different computational methods, ωB97X-D/6-311G(d, p) was selected due to its reasonable accuracy and better efficiency in predicting the torsional barrier heights of conjugated polymers (Supplementary Fig. 4). We found that Pz and 2FT combination shows the highest 〈cos^2^*φ*〉 value of 0.9329, i.e., the highest planarization, which could be attributed to its highest torsional barrier as well as its lowest relative energy at 0°. Electronegative atom N on the ring increases the rotational barriers by enhancing the backbone conjugation^31^. The fluorine has little steric hindrance and provides attractive non-covalent interactions, which also plays a significant role in promoting the planarization of Pz-2FT (see detailed discussions in the Supplementary Information Section 2). Thus, based on these building blocks, P(PzDPP-2FT) was designed (Fig. 1b), in which the torsion angles are small (*φ*_1_ = *φ*_2_ = 0.002°). For comparison, reference polymers, P(PzDPP-4F2T) and P(PzDPP-T), with similar chemical structures were also synthesized (Fig. 1b). DFT calculations reveal that the three polymers exhibit coplanar backbones from the side view under the optimized conformations (Supplementary Fig. 8). PES results demonstrate that Pz-DPP tends to form planar connections for all the polymers (*φ*_1_, *φ*_3,_ and *φ*_6_) due to the non-covalent O ∙ ∙ ∙ H bonding (Fig. 1c). However, the small torsional barrier between the two thiophene units (*φ*_5_) in P(PzDPP-4F2T) makes the polymer readily deviate from planarity. Even though the dihedral angle of the Pz-T (*φ*_7_) in P(PzDPP-T) exhibits a relatively high torsional barrier, the low torsional barriers at both 0° and 180° make P(PzDPP-T) have more than one preponderant conformation. Interestingly, the strong F ∙ ∙ ∙ H interaction at Pz-2FT brings about a steeper and higher torsional barrier, and only one preponderant conformation at 0°. These two crucial features promote P(PzDPP-2FT) to form narrower torsion-angle distributions and thus low conformation disorder. Compared with the pseudo-linear backbone of both P(PzDPP-4F2T) and P(PzDPP-T), P(PzDPP-2FT) has a zigzag backbone curvature (Fig. 1d and Supplementary Fig. 8). We will show that this structure feature allows P(PzDPP-2FT) to form unique binding sites with *n*-dopants and further reducing the energetic disorder after doping.

### Polymer properties

**Fig. 2:**
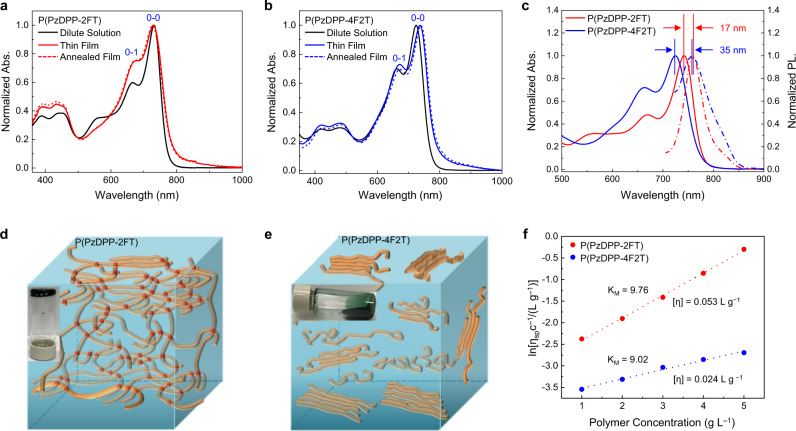
Comparison of the optical properties, the microstructures, and interchain interactions. **a**, **b** Normalized absorption spectra of both P(PzDPP-2FT) and P(PzDPP-4F2T) in solution, thin film, and annealed film. **c** Normalized absorption (solid lines) and photoluminescence (dash lines) (*λ*_ex_ = 460 nm) spectra of the polymer solutions. **d**, **e** Schematic illustration of the possible microstructures of both polymers in solution. The red dot in the figure represents the crosslinking points or strong interchain short contacts. **f** Viscosity comparison of both polymer solutions as a function of concentration at 20 °C.

According to the cyclic voltammetry measurement, the estimated LUMO energy levels of P(PzDPP-2FT) and P(PzDPP-4F2T) are −3.90 eV and −3.82 eV, respectively (Supplementary Fig. 12). As P(PzDPP-2FT) and P(PzDPP-4F2T) have very similar chemical structures and close LUMO energy levels, we will focus on the comparison of their properties and device performance. Previous study has shown that the absorption profiles of polymers can reflect the rigidity and coplanarity of polymer backbones^32^. Conjugated polymers with larger torsional angles will become more planar in solid state due to interchain *π*–*π* stacking, leading to a red-shift in the solid-state absorption spectra. The thin-film absorption spectra of P(PzDPP-2FT) do not exhibit a noticeable shift compared to its solution one (Fig. 2a), suggesting that P(PzDPP-2FT) has a rigid and coplanar backbone with similar molecular conformations in both solution and solid state. In contrast, the film absorption spectrum of P(PzDPP-4F2T) exhibited an obvious red-shift compared to solution one (Fig. 2b), suggesting that P(PzDPP-4F2T) has a relatively flexible backbone and it may adopt more planar backbone conformation in solid state. Moreover, it has been proved that conjugated polymers with more planar and rigid backbone usually exhibit smaller Stokes shift and smaller full width at half maxima (FWHM) of their absorption and emission peaks^33,34^. Figure 2c shows that the Stokes shift of P(PzDPP-2FT) is 17 nm, which is much smaller than that of P(PzDPP-4F2T) (35 nm). The FWHM of the 0-0 absorption and emission peaks of P(PzDPP-2FT) are 41.1 and 39.5 nm, respectively, which are also smaller than that of P(PzDPP-4F2T) (43.8 and 41.6 nm for the absorption and emission peaks, respectively) (Supplementary Fig. 15). These results indicate that P(PzDPP-2FT) has a more planar and more shape-persistent backbone than P(PzDPP-4F2T).

We observed that the P(PzDPP-2FT) solution can form a gel when aged at room temperature, whereas the P(PzDPP-4F2T) solution remains fluid after aging even though both polymers have similar solubility (Fig. 2d and Supplementary Fig. 28). As gel formation usually requires the presence of a cross-linked polymer chain network that percolates the system and confines the solvent^35^, we assume that P(PzDPP-2FT) may have strong interchain short contacts. To further prove this, viscosity was employed to evaluate properties of both polymers in solutions. As was expected, P(PzDPP-2FT) always exhibit higher viscosity and larger Martin constant *K*_M_ (reflect the polymer–polymer interaction^36^) than P(PzDPP-4F2T). The intrinsic viscosity [*η*] of P(PzDPP-2FT) is over two times higher than that of P(PzDPP-4F2T), indicating the existence of larger macromolecules or aggregates^37^. These results confirm that P(PzDPP-2FT) has short interchain contacts or crosslinking points in solution to form such strong interactions, which will be further discussed and supported by the photothermal deflection spectroscopy (PDS) measurement.

### Charge-transport properties and thin-film characterization

**Fig. 3:**
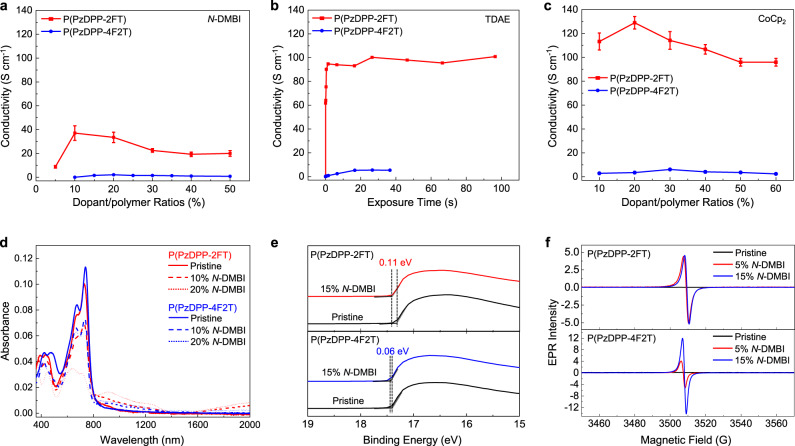
*N*-doped electrical conductivities and doping efficiency comparison. **a** Electrical conductivities at different *N*-DMBI/polymer ratios. **b** Electrical conductivities after different exposure times in TDAE vapor. **c** Electrical conductivities at different CoCp_2_/polymer ratios. The error bars are determined according to 2% measurement errors. **d** Absorption spectra of the pristine and *N*-DMBI-doped P(PzDPP-2FT) and P(PzDPP-4F2T) films. **e** UPS binding energy of the pristine and 15% *N*-DMBI-doped films. **f** EPR signals of the pristine and *N*-DMBI-doped films at different dopant/polymer ratios.

Various mass fractions of 4-(1,3-dimethyl-2,3-dihydro-1H-benzoimidazol-2-yl)phenyl)dimethylamine (*N*-DMBI) were mixed with P(PzDPP-2FT) and P(PzDPP-4F2T) to optimize their electrical conductivities. P(PzDPP-2FT) has a maximum conductivity of 43.3 S cm^−1^, which is almost 20 times higher than that of P(PzDPP-4F2T) (Fig. 3a). To eliminate the influence of different dopants and doping methods, the two polymers were also doped with tetrakis(dimethylamino)ethylene (TDAE) and bis(cyclopentadienyl)cobalt (CoCp_2_). In both cases, P(PzDPP-2FT) still showed higher *n*-type electrical conductivities than P(PzDPP-4F2T) (Fig. 3b, c). Notably, after being doped with CoCp_2_, P(PzDPP-2FT) exhibits the highest *n*-type electrical conductivity of 129 S cm^−1^. As both TDAE and CoCp_2_ are small and highly diffusible dopants (Supplementary Fig. 23), the conductivity enhancement is probably due to their better permeability and miscibility with the polymers, which can be supported by the uniform microstructures of the polymer films at higher doping concentrations (Supplementary Fig. 24). Besides, the temperature-dependent electrical conductivity of the doped P(PzDPP-2FT) shows weaker temperature dependence compared with that of P(PzDPP-4F2T) (Supplementary Fig. 17), suggesting that P(PzDPP-2FT) has lower charge-transport barriers after *n*-doping.

The doping efficiencies of the films were studied by absorption spectroscopy, ultraviolet photoelectron spectroscopy, and X-ray photoelectron spectroscopy (XPS). At each dopant/polymer ratio, P(PzDPP-2FT) showed stronger (bi)polaron absorptions than P(PzDPP-4F2T) (Fig. 3d). The larger shifts of the onset of the secondary electron cutoff for doped P(PzDPP-2FT) also imply that the Fermi level of P(PzDPP-2FT) shifts higher than that of P(PzDPP-4F2T) (Fig. 3e). The doping efficiency could also be evaluated by the newly formed signal of *N*-DMBI^+^ at 402 eV with XPS. For each dopant/polymer ratio, the relative intensity of the cationic *N*-DMBI^+^ to other *N* (1 s) signals in P(PzDPP-2FT) film is larger than that of P(PzDPP-4F2T) (Supplementary Fig. 18). The electron paramagnetic resonance (EPR) (Fig. 3f) and alternating current (AC) magnetic field Hall (Supplementary Fig. 19) measurements also showed that the *N*-DMBI-doped P(PzDPP-2FT) exhibited higher charge-carrier concentrations than P(PzDPP-4F2T). These results indicate that P(PzDPP-2FT) exhibited higher doping levels than P(PzDPP-4F2T) in film.

Doping efficiency enhancement is usually attributed to the low-lying LUMO energy levels of the polymer or the enhanced miscibility between polymer and *n*-dopant. However, P(PzDPP-2FT) shows almost the same LUMO energy level as P(PzDPP-4F2T), which is relatively high among other *n*-type polymers reported in the literature (Supplementary Fig. 12 and Supplementary Table 4). Therefore, the slightly lower LUMO level of P(PzDPP-2FT) cannot explain its high doping levels. The microstructures of both the pristine and the *N*-DMBI-doped P(PzDPP-2FT) and P(PzDPP-4F2T) films were characterized by grazing incidence wide-angle X-ray scattering (GIWAXS). The two-dimensional (2D) GIWAXS patterns of the pristine polymer films are displayed in Supplementary Fig. 20. P(PzDPP-4F2T) exhibits stronger (*h*00) diffraction peaks than P(PzDPP-2FT) along the *q*_*z*_ axis, indicating that P(PzDPP-4F2T) has a highly ordered polymer packing, whereas P(PzDPP-2FT) has lower crystallinity. P(PzDPP-2FT) mainly exhibits a face-on orientation, whereas P(PzDPP-4F2T) exhibits mixed face-on and edge-on orientations. The *π*–*π* stacking distances were estimated to be 3.48 Å and 3.37 Å for P(PzDPP-2FT) and P(PzDPP-4F2T), respectively. After *N*-DMBI doping, the lamellar packing and *π*–*π* stacking distances of P(PzDPP-4F2T) remain almost unchanged, whereas a slight increase of both the lamellar packing and *π*–*π* stacking distances was observed for P(PzDPP-2FT) (Supplementary Fig. 21). The paracrystallinity (*g*) change of both polymers is shown in Supplementary Fig. 22^27^. Usually, after heavily doping, polymer films showed increased paracrystallinity due to dopant-induced structural disorder. Interestingly, the paracrystallinity of P(PzDPP-2FT) does not change much even for dopant/polymer ratios of up to 60%, suggesting its tolerance to dopant-induced structural disorder. Atomic force microscope (AFM) combined with infrared-spectroscopy can be used to probe the dopant miscibility in polymer films^38^. When doped with 30% *N*-DMBI, the P(PzDPP-2FT) film exhibited a domain size of around 50 nm, much smaller than that of the doped P(PzDPP-4F2T) film (90–110 nm) (Supplementary Fig. 27). Moreover, the 30% *N*-DMBI doped P(PzDPP-4F2T) film has large doped polymer domains separated by a large amount of unreacted dopant (Supplementary Fig. 27b). In contrast, the doped P(PzDPP-2FT) film has smaller polymer domains and a tiny amount of unreacted dopant (Supplementary Fig. 27a). Therefore, the enhanced doping efficiency of P(PzDPP-2FT) is attributed to its good miscibility with dopants.

### Disorder-tolerant features

**Fig. 4:**
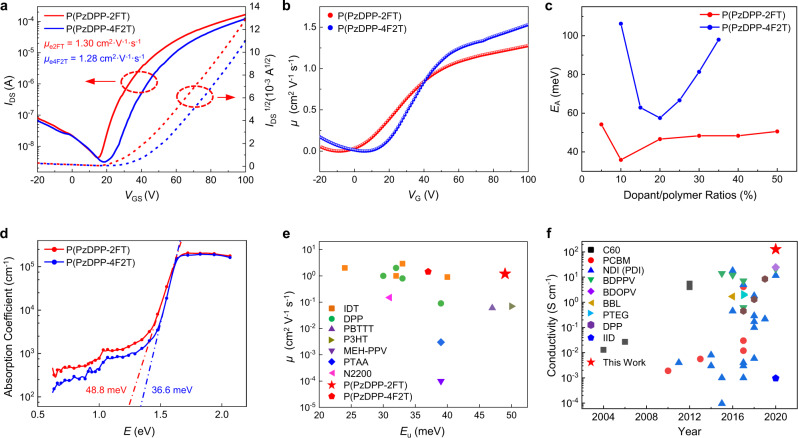
Comparison of the charge-transport properties, Urbach energies, and activation energies. **a** Transfer characteristics of the pristine P(PzDPP-2FT) and P(PzDPP-4F2T) films. **b** Gate-voltage-dependent saturation mobilities. **c** Extracted activation energies of the doped films at different dopant/polymer ratios. **d** Absorption coefficients of both the pristine polymer films measured by PDS. Dash lines represent exponential tail fits for extraction of the Urbach energies. **e** Mobility vs. Urbach energy of ours and other reported polymers. **f** Electrical conductivity comparison of reported *n*-doped semiconducting polymers.

The pristine P(PzDPP-2FT) exhibited comparable electron mobilities (1.30 ± 0.14 cm^2^ V^−1^ s^−1^) to that of P(PzDPP-4F2T) (1.28 ± 0.25 cm^2^ V^−1^ s^−1^) (Fig. 4a). The gate-voltage-dependent mobilities of P(PzDPP-2FT) and P(PzDPP-4F2T) were extracted from their transfer characteristics (Fig. 4b). For P(PzDPP-2FT), the mobility is nearly independent of the gate voltage (*V*_G_) when *V*_G_ > 50 V, whereas the mobility of P(PzDPP-4F2T) increases as *V*_G_ increases. The temperature-dependent drain current *I*_D_ vs*. V*_G_ in the saturation regime was tested (Supplementary Fig. 35). For P(PzDPP-2FT), the exponent *γ*, which reflects the temperature dependence, takes a nearly temperature-independent value of about 3.3 (Supplementary Fig. 36). In contrast, the exponent *γ* for P(PzDPP-4F2T) increases when decreasing temperature, where *γ* = *T*_0_/*T* + 2. This can be explained by the carrier hopping within an exponential density of states (DOSs)^26,39^. The temperature-dependent mobility measurement shows that P(PzDPP-2FT) has lower activation energy (*E*_a_) than P(PzDPP-4F2T) (Supplementary Fig. 37). All these results suggest that the pristine P(PzDPP-2FT) has lower energetic disorder than P(PzDPP-4F2T)^26^. Previous studies demonstrated that disorder in semiconductors would result in the formation of the tail states of their DOSs, broadening of the absorption onset, and creating an exponential sub-bandgap absorption tail called the Urbach tail. The Urbach energy (*E*_u_) is often used to evaluate the energetic disorder of a conjugated polymer film^26,40^. According to PDS characterization, P(PzDPP-2FT) exhibits a larger Urbach energy (48.8 meV) than P(PzDPP-4F2T) (36.6 meV) (Fig. 4d). This seems to conflict with our device results that P(PzDPP-2FT) has lower energetic disorder. In fact, the disorder in conjugated polymer film could come from the torsional disorder along the polymer backbone or different molecular packings. A conjugated polymer film usually contains crystalline, semi-crystalline, and amorphous regions. Compared with the crystalline region, the DOS broadening in the semi-crystalline and the amorphous regions is more significant (further discussed in the theoretical modeling part). The GIWAXS results suggest that P(PzDPP-2FT) is less crystalline with more disordered polymer packing. As P(PzDPP-2FT) has a rigid and planar backbone, its larger Urbach energy does not come from the torsional disorder along the polymer backbone. Therefore, the larger Urbach energy of P(PzDPP-2FT) can be attributed to its low crystallinity and less ordered molecular packing. However, charge-transport properties in polymer film are mostly influenced by the DOS along the charge-transport pathways. We also compared the charge-carrier mobilities and *E*_u_ data of different types of polymers reported in the literature. It turns out that there is no clear relationship between charge-carrier mobility and *E*_u_ (Fig. 4e). Interestingly, for the polymers with high mobilities over 1 cm^2^ V^−1^ s^−1^, P(PzDPP-2FT) has the largest *E*_u_. This result can be explained by a previous assumption that fast intrachain charge transport and a few interchain short-range contacts are sufficient to allow high charge-carrier mobilities in conjugated polymers^27^. Recent studies have indicated that a stronger sub-bandgap absorption coefficient area in PDS suggests the existence of stronger interchain short contacts within materials’ amorphous domains^41^. The larger sub-bandgap area of P(PzDPP-2FT) in PDS measurements also suggests it has stronger interchain short-range interactions (Supplementary Fig. 39), consistent with the gel formation experiment and the higher viscosity of P(PzDPP-2FT) solution. In addition, the DFT calculations, absorption profiles, and Stokes shifts all support that P(PzDPP-2FT) has a rigid and single preferential planar conformation, which guarantees its fast intrachain charge transport. Therefore, the high electron mobility of P(PzDPP-2FT) can be attributed to its efficient intrachain charge transport and good interchain short-range interactions.

The activation energies of the polymer films at different doping concentrations were extracted (Supplementary Fig. 40). P(PzDPP-2FT) exhibited constantly lower activation energies than P(PzDPP-4F2T) (Fig. 4c). The activation energies of both P(PzDPP-2FT) and P(PzDPP-4F2T) first decrease and then increase as the dopant concentration increasing. Molecular doping of a disorder organic semiconductor has two counteracting effects. On the one hand, it increases the charge-carrier concentration and fills the traps, leading to a reduced activation energy for charge transport^42^. On the other hand, the insertion of the ionized dopants causes structural and energetic disorder because of film microstructure damage and the randomly distributed Coulombic interactions between the dopant ions and the charge carriers. These effects result in increased activation energy, especially at high dopant concentrations^43^. Therefore, the decrease of the activation energy might be due to the trap-filling mechanism and the increase of the activation energy could be attributed to the excessive dopant-induced structural and energetic disorder^44^. Compared with P(PzDPP-4F2T), P(PzDPP-2FT) shows a smaller activation energy fluctuation with the change of the doping concentration and the excessive dopants (>20%) did not obviously increase the activation energy. These results suggest that P(PzDPP-2FT) has a stronger tolerance to the dopant-induced energetic disorder^27,45^. Therefore, the high miscibility with dopants and disorder-tolerant feature of P(PzDPP-2FT) allows it to show high *n*-type electrical conductivities (Fig. 4f).

P(PzDPP-T) has a coplanar and linear backbone with relatively high torsional barriers but multiple coplanar conformations (i.e., at 0° and 180°). It showed low electrical conductivity of 0.01 S cm^−1^ when doped with TDAE or *N*-DMBI (Supplementary Fig. 34). The EPR measurement indicated that the doped P(PzDPP-T) films had a charge-carrier concentration of ca. 10^19^ cm^−3^, comparable to that of P(PzDPP-2FT) and P(PzDPP-4F2T) (Supplementary Fig. 33 and Supplementary Table 1). Therefore, the low electrical conductivity of the doped P(PzDPP-T) film is primarily attributed to its low charge-carrier mobility or significant structural and energetic disorder. This result suggests that apart from a planar backbone, a single dominant conformation is also important to realize the disorder-tolerant feature.

### Theoretical modeling

**Fig. 5:**
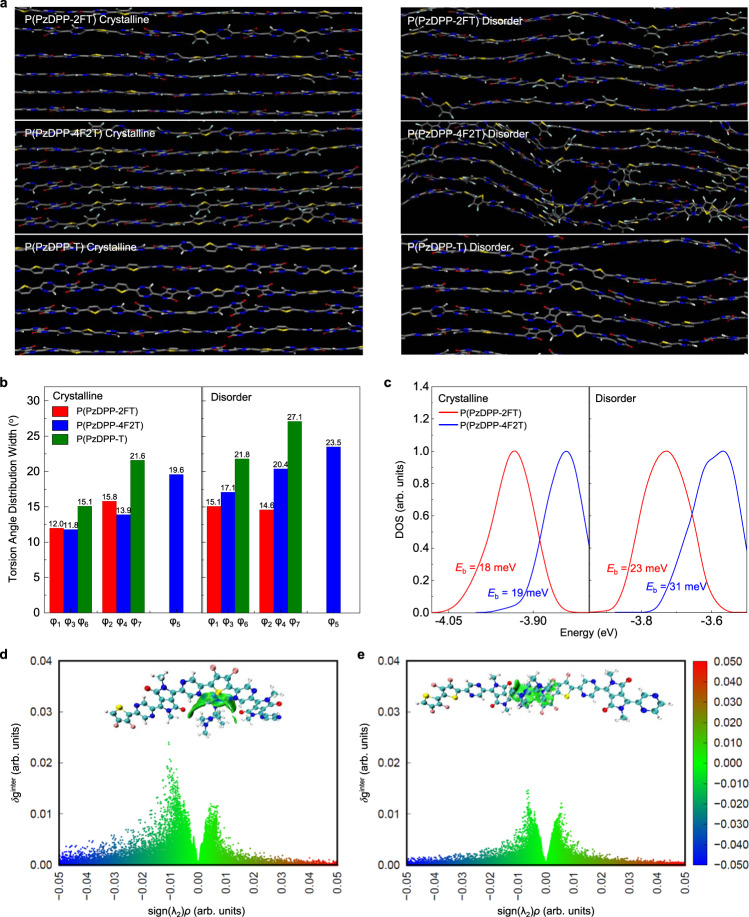
Computational studies. **a** MD simulations of the polymers in crystalline and disorder regions. **b** Torsion-angle distributions for dihedral angles along polymer backbone (*φ*_1_ ~ *φ*_7_). The torsion-angle distribution width (W) based on the Gaussian fitting could be considered as the SD from planarity. **c** Calculated DOS distributions for dodecamers of both polymers. **d**, **e** *δ*_g_^inter^-isosurface and *δ*_g_^inter^-sign(*λ*_*2*_)*ρ* 2D fingerprint plots for illustrating the different intermolecular interactions between **d** P(PzDPP-2FT)^−^-TDAE^+^ and **e** P(PzDPP-4F2T)^−^-TDAE^+^.

To further understand the difference among the three polymers in molecular scale and the importance of our design rules, we performed atomistic molecular dynamics simulations (see Supplementary Information for more details). Crystalline and disordered regions in polymer films are critical for interchain and intrachain charge transport^27^. Figure 5a, b show that both P(PzDPP-2FT) and P(PzDPP-4F2T) exhibit narrower torsion-angle distributions and more planar backbone in the crystalline region compared with P(PzDPP-T). This result is consistent with the better planarity of the fluorine-substituted polymers according to design Rule (1). Disorder regions may become more critical in doped conjugated polymers, as previous studies have shown that dopants tend to insert into the side chain or/and *π*–*π* stacking zones, perturbing the polymer packing and cause large structural disorder^46^. We then expanded the *π*–*π* stacking and lamellar distances of the polymers to simulate the disorder regions or the dopant-induced disorder regions. Without the restrain of tight *π*–*π* stacking, P(PzDPP-4F2T) shows obviously broadened torsion-angle distributions and its backbone severely deviates from planarity, whereas P(PzDPP-2FT) can still maintain planar conformation, thanks to its high and steep torsional barrier in each dihedral angle. The shape-persistent backbone and narrower torsion-angle distributions in P(PzDPP-2FT) provide the polymer with good interchain interactions, as demonstrated in the gelation experiment. As for P(PzDPP-T), it not only displays the widest dihedral angle distribution due to its low torsional potentials but also twists partly on account of its non-unique preponderant conformation at 0° or 180°, further emphasizing the importance of Rule (2) and Rule (3). To quantify the degree of disorder, we calculated the DOSs via DFT and compared the characteristic depth of the trap states (*E*_b_) of the two fluorinated polymers. In crystalline regions, both polymers exhibit similar *E*_b_ of the DOS of LUMO energy level. When turned to disorder regions, the *E*_b_ of P(PzDPP-2FT) increased slightly from 18 to 23 meV, compared to P(PzDPP-4F2T) from 19 to 31 meV, outlining that the former owns a better disorder-tolerant ability. Thus, charge carriers in P(PzDPP-2FT) can transport more efficiently with less energetic disorder after dopants are added^47^. Considering the large proportion of disorder regions in polymer films, the intrachain charge transport in these disorder regions may strongly affect the whole charge-transport characteristics. All these results allow us to conclude that P(PzDPP-2FT) undoubtedly has excellent transport properties and superior disorder-tolerant ability.

We also performed DFT calculations to explore the intermolecular interactions between the polymers and the dopants. Using TDAE as an example, we found that P(PzDPP-2FT) has a stronger binding energy with TDAE^+^, because the cationic TDAE^+^ tends to dock into the “cavity” formed by the zigzag backbone (Supplementary Fig. 44 and Supplementary Table 3). Independent Gradient Model (IGM) analysis in Fig. 5d suggests that the major interactions between TDAE^+^ and P(PzDPP-2FT)^−^ are hydrogen bonding and Van der Waals interactions, whereas only weaker Van der Waals interactions are found for P(PzDPP-4F2T)^−^-TDAE^+^ fragment. According to the IGM analysis, non-zero values of *δ*_g_^inter^ exclusively correspond to interaction situations: the larger the *δ*_g_^inter^ value, the stronger the interactions^48^. Therefore, larger *δ*_g_^inter^ in the 2D fingerprint plot of P(PzDPP-2FT)^−^-TDAE^+^ supports the stronger intermolecular interaction and thus larger binding energy, compared to that of P(PzDPP-4F2T)^−^-TDAE^+^ (Fig. 5d and Supplementary Table 3). During the geometry-optimization process, we found that TDAE^+^ was easily captured in the “cavity” formed by the zigzag backbone of P(PzDPP-2FT), whereas it tended to stay on the top of the P(PzDPP-4F2T) backbone. This could result in serious disruption of the polymer packing in P(PzDPP-4F2T) or refraining the TDAE from doping. These results indicate that the zigzag backbone curvature can effectively capture TDAE dopants without much destruction to polymer packing and lead to enhanced miscibility and higher doping efficiency in P(PzDPP-2FT) films.

## Discussion

 In conclusion, with three design rules and a computer-aided building block screening process, we have successfully designed and synthesized a disorder-tolerant *n*-type polymer, P(PzDPP-2FT). Our results indicate that a highly dopable and disorder-tolerant polymer might need the following features: (i) high and steep torsion-angle barriers to prevent any distortions caused by dopants; (ii) single preferential planar conformation; (iii) strong interchain interactions (as indicated by the gel formation experiment) that can endure dopant interference and provide efficient interchain charge transport; and (iv) strong dopant-binding sites that do not disturb polymer packing. We envisage that our disorder-tolerant design could provide a valuable strategy for highly dopable and highly conductive conjugated polymers.

## Supplementary information

Supplementary Information

## Data Availability

The source data generated in this study have been deposited in the materials cloud database (https://archive.materialscloud.org/record/2021.127) and are also available from the corresponding author upon reasonable request.

## Code availability

The codes that support the findings of this study are available from the corresponding author upon reasonable request.
