# Supplementary material for: Approaching disorder-tolerant semiconducting polymers
Source: Nat Commun. 2021 Sep 29;12:5723. doi: 10.1038/s41467-021-26043-y (PMC8481336; doi:10.1038/s41467-021-26043-y)
Supplement: Supplementary file 1 — Supplementary Information [file 41467_2021_26043_MOESM1_ESM.pdf]

Supplementary Information

For

Approaching disorder-tolerant semiconducting polymers

Xinwen Yan#, Miao Xiong#, Xin-Yu Deng, Kai-Kai Liu, Jia-Tong Li, Xue-Qing Wang, Song Zhang, Nathaniel Prine, Zhuoqiong Zhang, Wanying Huang, Yishan Wang, Jie-Yu Wang, Xiaodan Gu, Shu Kong So, Jia Zhu, Ting Lei*

Correspondence to: tinglei@pku.edu.cn

This PDF file includes:

Supplementary Methods and Discussions
Section 1. Device fabrication and characterization
Section 2. Computer-aided polymer building block screening process
Section 3. DFT-optimized geometries and PES calculation
Section 4. Characterization of molecular weights, thermal properties, electrical and optical properties
Section 5. Temperature-dependent conductivity (σ) measurement
Section 6. The doping efficiency comparison of P(PzDPP-2FT) and P(PzDPP-4F2T)
Section 7. AC Hall measurements
Section 8. Microstructure characterization of the pristine and *N*-DMBI doped polymers
Section 9. AFM-IR characterization
Section 10. Solubility evaluation for P(PzDPP-2FT) and P(PzDPP-4F2T).
Section 11. Field-effect transistor (FET) devices fabrication and characterization
Section 12. Photothermal deflection spectroscopy (PDS)
Section 13. Temperature-dependent electrical conductivities.
Section 14. Molecular dynamics calculations
Section 15. Visualization of weak interaction and calculation of binding energy
Section 16. Time-dependent decay of the electrical conductivity
Section 17. Synthesis of P(PzDPP-2FT), P(PzDPP-4F2T), and P(PzDPP-T).
Supplementary References

Supplementary Methods and Discussions

Section 1. Device fabrication and characterization

Device fabrication and conductivity measurement. The doping method used in this study is solution blending. P(PzDPP-2FT) was dissolved in 1,2-dichlorobenzene (*o*-DCB) with a concentration of 3 g/L. P(PzDPP-2FT) solution was blended with a dopant solution as a function of doping ratio at room temperature. All devices for conductivity measurements were fabricated using glass substrates. Thin films were deposited on the substrates by spin-coating the mixed solution at 2000 rpm for 60 s and annealed at 120 ^{o}C for 2 h. The conductivity was collected by four-probe measurements in a N_2 glove box with Keithley 4200 SCS semiconductor parameter analyzer. The film thickness was determined by AFM.

Characterization. UPS and XPS were conducted on a Kratos AXIS Ultra Photoelectron Spectrometer under an ultrahigh vacuum of about 3×10^{-9} Torr with unfiltered He I gas discharge lamp source (21.22 eV) and a monochromatic Al Kα source (1486.7 eV, θ = 90°) as the excitation source. Al Kα source operated at 14 kV and 15 mA. The instrumental energy resolution for UPS and XPS were 0.1 eV and 0.5 eV, respectively. Data analysis was performed by CasaXPS software. All films were deposited on a gold-coated substrate (60 nm of gold with a 15 nm of chromium adhesion layer deposited by thermal evaporation). Polymers were then spin-coated onto the substrates. The semiconducting film was removed from one edge of the sample by swabbing with chlorobenzene to expose the underlying gold layer. Silver ink was used to electrically connect the exposed gold on the top of the substrates to the airtight sample holder in order to prevent charging. The samples were transferred through a transport system without air exposure into the spectrometer analysis chamber. AFM-IR measurements were performed using Bruker Anasys nanoIR3 system equipped with a HyperSpectra QCL (800~1800 cm^{-1}) laser source.

Section 2. Computer-aided polymer building block screening process

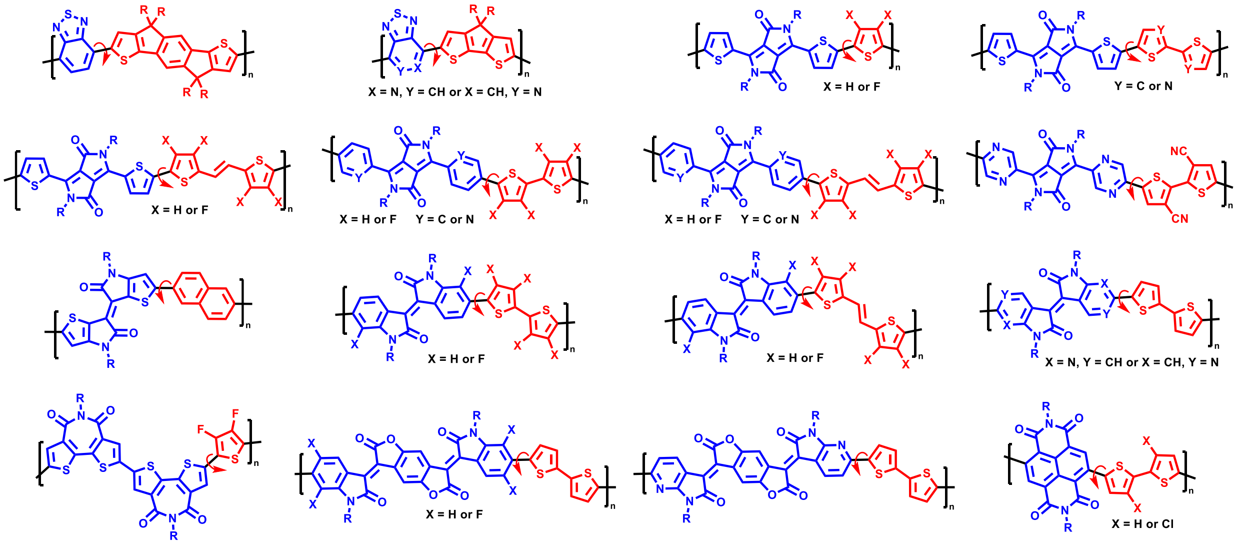

Supplementary Figure 1. High-performance D-A copolymers used in OFETs. We collected semiconducting polymers that were reported to have outstanding charge transport properties from recently published reviews[1,2]. These polymers are usually donor-acceptor (D-A) copolymers. The torsional angles between the donor and acceptor building blocks contribute to a large part of the energetic disorder in conjugated polymers. According to the previous studies[3], large torsion angles in polymer backbone will significantly increase the structural and energetic disorders and finally impact the charge transport properties in film. To construct conjugated polymers with high intrachain charge transport properties, a coplanar backbone conformation and narrow torsion angle distribution are essential. To find out the desired polymer building block combinations, we performed torsional potential energy scans (PES) of the dihedral angles in different polymer building block combinations. To simplify the DFT calculations, we divided these polymer building blocks into two parts: acceptors and donors (See Supplementary Fig. 2).

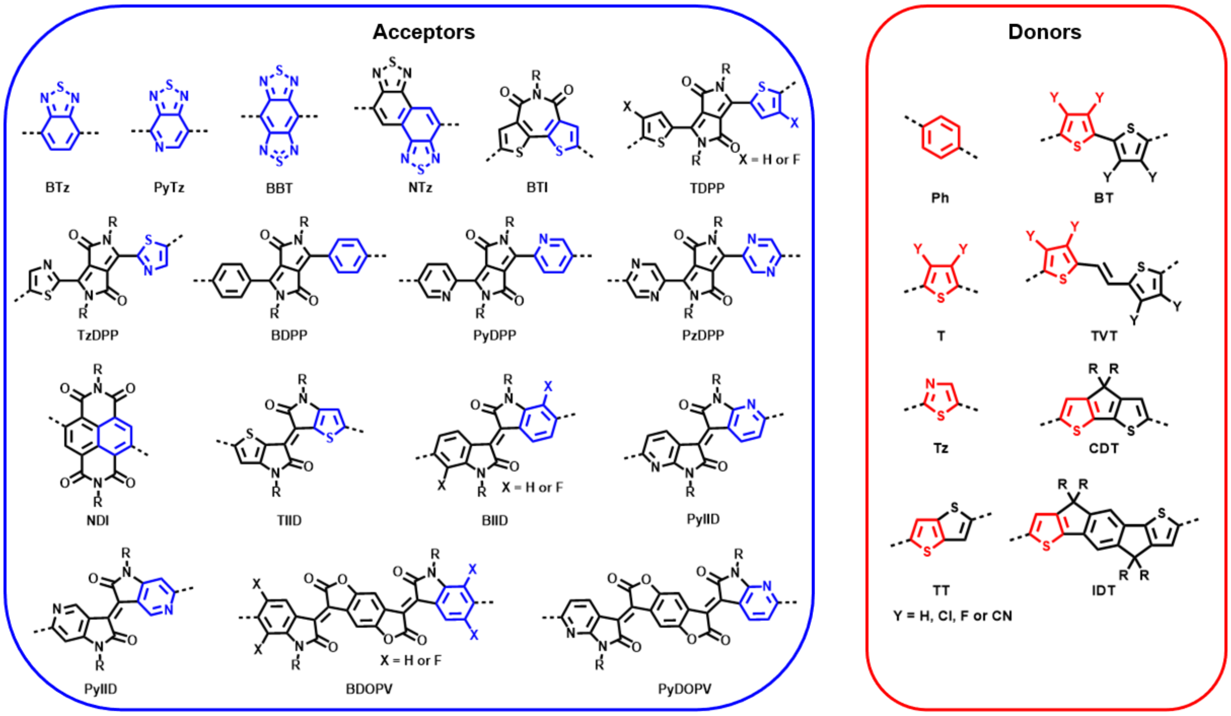

Supplementary Figure 2. Acceptors and donors that are commonly used to construct high-performance D-A semiconducting polymers. The acceptors usually exhibit large conjugated structures, while the donors usually are thiophene-based molecular fragments. The attached functional group in these building blocks will greatly affect the calculation results of torsional potential energy scans (PES). Among the various building blocks that are used to construct high-performance D-A copolymers, NDI is a special case that its protruded carbonyl group imposes a significant steric hindrance on the neighboring thiophene derivatives. However, this effect is very small in most other building blocks such as DPP, IID, and BDOPV. Clearly, the dihedral angles between NDI and the commonly used donor building blocks are always large. In contrast, without the steric hindrance, the dihedral angles between the other acceptor building blocks (DPP, IID, and BDOPV) and donor building blocks are small. It's obvious that NDI does not meet the design principles of coplanar backbone polymers, so we choose other acceptor building blocks such as DPP, IID, and BDOPV for the PES calculation. Of course, the PES calculations will be more accurate if the complete structure of building blocks is used. The work would be huge if we calculated all the combinations of donor and acceptor building blocks. Even though the overall charge distribution of the molecular fragment influences the results of torsional potential energy scans, the torsion barrier is mainly affected by the structure of the two directly connected segments (such as the blue-marked segments in the acceptors and the red-marked segments in the donors).

On the other hand, to construct high-performance n-type D-A conjugated polymers, a general strategy is to introduce electron-deficient groups into the acceptor and donor fragments at the same time. Therefore electron-rich fragments like Ph, T, TT, CDT, and IDT are not in our consideration. Based on this guidance, we further extracted some segments for further calculations (Supplementary Fig. 3).

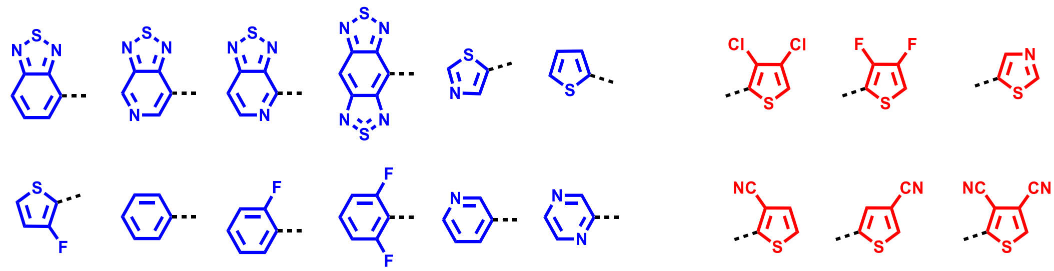

Supplementary Figure 3. Segments that are extracted for PES calculations. We combine these two groups of segments and calculate the corresponding torsional barriers. 3,4-Difluorothiophene unit was first used for calculation. We select desired polymer segments based on three criteria: (i) minimal energy is around 0° or 180° to guarantee good polymer planarity; (ii) single preferential planar conformation; (iii) highest torsional barrier and steepest energy changes to narrow conformation distributions. Some calculation results are detailed in Supplementary Fig. 5.

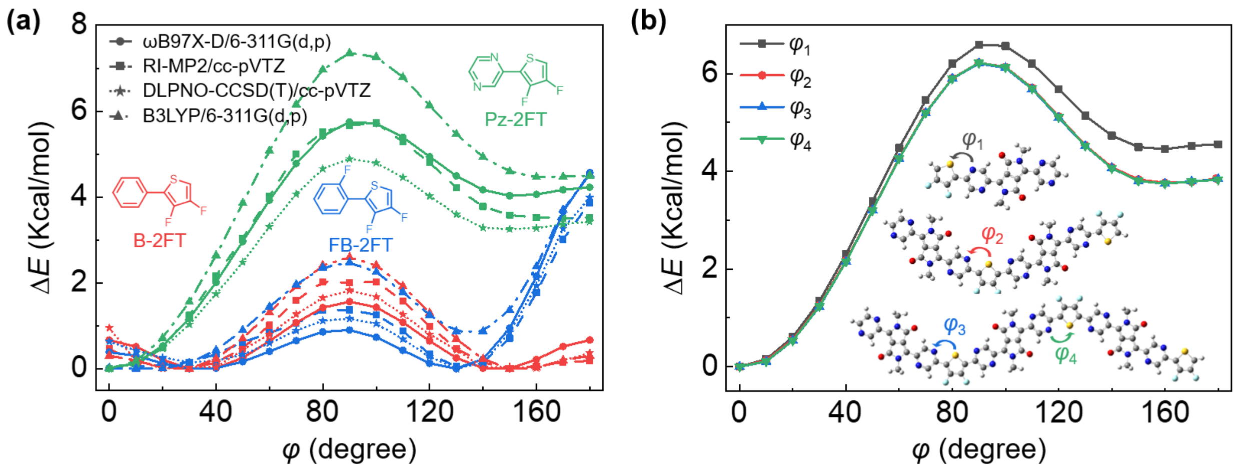

Supplementary Figure 4. (a) Torsional potential energy comparison for Pz-2FT (green curves), B-2FT (red curves) and FB-2FT (blue curves) using different computational methods. (b) Torsional potential energy curves for a series of P(PzDPP-2FT) oligomers.

To provide accurate torsional analysis, we compared the performance of several computational methods in potential energy scan (PES). As shown in Supplementary Fig. 4a, two popular DFT functionals (B3LYP and ωB9X-D), electron-correlation method (RI-MP2), and coupled-cluster theory (DLPNO-CCSD(T)) were assessed for different building block combinations. PES was computed at 10° intervals by fixing the dihedral angle. The domain-based local pair natural orbital coupled-cluster method (DLPNO-CCSD(T)) is used because it is considered approaching the gold standard CCSD(T) method in determining the barrier heights with less than 0.4 kcal/mol standard deviation[4]. Here, ωB97X-D, which is concluded to be the most reliable functional for conducting polymers[5], shows the best agreement to DLPNO-CCSD(T)/cc-pVTZ. The resolution of the identity second-order Moller-Plesset perturbation theory (RI-MP2), known to produce nearly identical to those of full MP2, also provides reliable barrier heights. We also considered the B3LYP functional with empirical dispersion corrections, even though it is well established to wrongly over-delocalize wave functions and thus maximize torsion potential barriers in conjugated polymers. While the PES is method-dependent, the observed trends are reproduced with different methods, allowing reliable comparisons for different building blocks calculated using the same functional. Hence, we selected ωB97X-D/6-311G(d, p) for the following analysis due to its reasonable accuracy and better efficiency in predicting barrier heights of conjugated materials, as well as its widespread use in the field which facilitates cross-comparisons.

We also examined how the PES of a backbone depended on its conjugation length. As shown in Supplementary Fig. 4b, monomer, dimer, and trimer were calculated for comparison. Clearly, the torsional barrier is not significantly affected by conjugation length, which suggests that PES for different pairs should allow us to predict the behavior of longer conjugated systems. Recently, a similar conclusion was also drawn for a range of conjugated systems based on torsional barrier calculation[6]. PES calculation results of other fragments based on ωB97X-D/6-311G(d, p) are detailed in Supplementary Fig. 5.

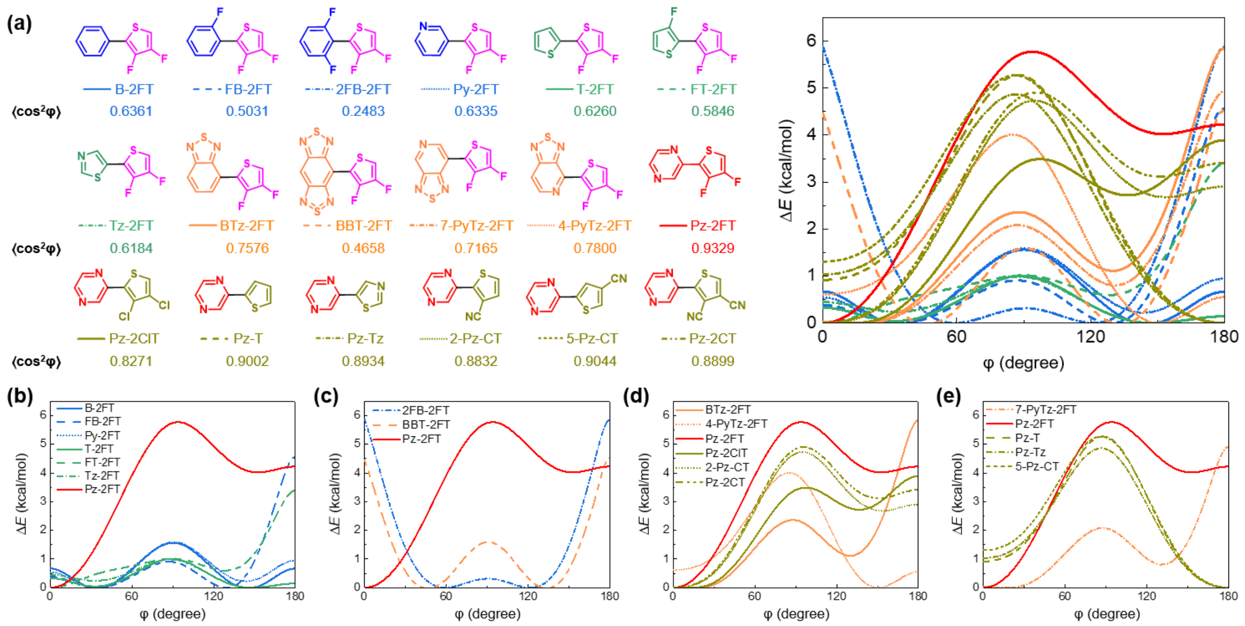

Supplementary Figure 5. Torsional potential energy scans (PES) of the dihedral angles in different polymer building block combinations (ωB97X-D/6-311G(d,p)). It's clear that the Pz-2FT has the minimum energy at 0°, the steepest and maximal torsion potential among all the segment combinations. As shown in Supplementary Fig. 5b, the B-2FT, Tz-2FT, T-2FT, and Py-2FT exhibit small torsional barriers in the range from 0° to 180°. Even though the FB-2FT and FT-2FT have large torsion barriers around 180°, the torsion barriers from 0° to 140° are still small, suggesting that these segment combinations have wide torsion angle distributions. In Supplementary Fig. 5c, it's clear that the 2FB-2FT and BBT-2FT have large torsion barriers around both 0° and 180°, indicating that these two fragments can hardly adopt a coplanar conformation. The 4-PyTz-2FT exhibits a relatively high torsion barrier around 90°; however, it has a wide torsion angle distribution from 140° to 220° (Supplementary Fig. 5d). The rest of these fragments have minimal energy at 0°, but they all exhibit less steep and lower torsion potential compared with Pz-2FT (Supplementary Fig. 5d & 5e). The previous study has summarized several key factors affecting the torsional potentials between building blocks, including steric repulsion, backbone conjugation, and attractive interaction[7]. Steric repulsion is the most important factor, which pushes the local minimum away from planar conformations, like B-2FT, and 2FB-2FT in Supplementary Fig. 5b and 5c. Strong electron-withdrawing substituent groups, such as cyano group, are found to reduce backbone conjugation resulting in reduced internal rotation barrier at 90°, e.g., Pz-Tz and Pz-T in Supplementary Fig. 5e. Attractive non-covalent interactions between

the substituents and rings contribute to the planarization at 0° as was established experimentally and computationally for oligomers and polymers with X···S and CH···X (where X = S, O, N, F) through space interactions[8]. We also observed the same trend in our PES results (e.g., Pz-2FT and Pz-T, Pz-2ClT and Pz-T, Pz-2CT, and 5-Pz-CT). To further analyze the relative complex weak interactions, we performed non-covalent interaction analysis (NCI)[9] of Pz-2FT as an example using the Multiwfn software[10].

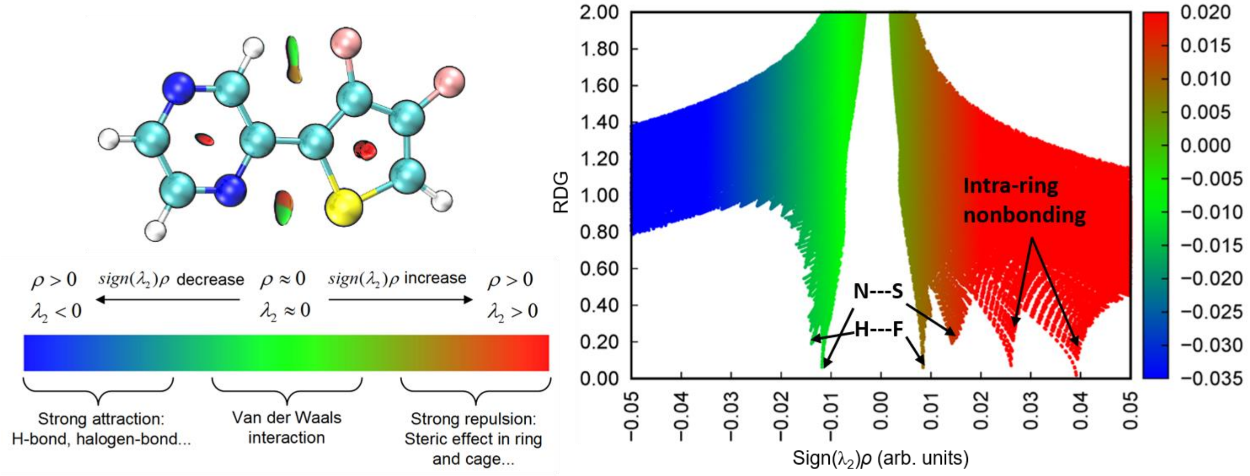

Supplementary Figure 6. NCI analysis for intramolecular interactions of Pz-2FT computed at the wB97X-D/6-311G(d,p). NCI is a method that searches for critical points in the electron density topology through the use of the reduced density gradient (RDG). When a singularity in the RDG is found, indicating an electron density critical point, the product of the sign of the second largest eigenvalue of electron density Hessian matrix and electron density (sign(λ_2)ρ) can be used to determine the strength of a particular nonbonding interaction[11]. NCI isosurfaces are generated with VMD[12] at RDG = 0.5 and are colored according to the values of sign(λ_2)ρ from −0.035 to 0.02, corresponding to evident attractive weak interaction (e.g., H-bond) and strong steric effect, respectively (as color bar shows in Supplementary Fig. 6). In addition, RDG vs. sign(λ_2)ρ scatter plot is presented to fully descript the topology of electron density, in which the singularities are labeled with the corresponding nonbonding interactions (Supplementary Fig. 6). It is clear from the analysis below that H···F and N···S interactions are attractive nonbonding interactions despite of little steric repulsion, in agreement with the conclusions from the torsional analysis that the favorable conformation of Pz-2FT at 0° are likely due to these types of interactions.

The planarity indexes ($\langle \cos^2\varphi \rangle$) of all these segment combinations were calculated, the Pz-2FT exhibited the highest $\langle \cos^2\varphi \rangle$ value of 0.9329, suggesting that Pz-2FT has the most rigid coplanar conformation. Although the $\langle \cos^2\varphi \rangle$ value has considered all torsional conformations and their relative contribution to the overall structural disorder, it is still not enough to screen the desired polymers with minimal dihedral angle distributions. For example, Pz-T and 5-Pz-CT also exhibited high $\langle \cos^2\varphi \rangle$ values over 0.9. However, PES results demonstrated that they have low torsion barriers at both 0° and 180°, suggesting that they have more than one preferential planar conformation. For comparison, the strong F···H hydrogen bonding interaction at Pz-2FT makes it have one and only preponderant conformation at 0°. In conclusion, newly designed D-A copolymers that containing Pz-2FT fragments should have coplanar backbone conformation, narrow torsion angle distribution, and single preferential planar conformation and thus exhibit high disorder-tolerant properties. Based on this guidance, we chose the PzDPP as the acceptor unit and 3,4-difluorothiophene as the donor unit to construct the new n-type semiconducting polymer.

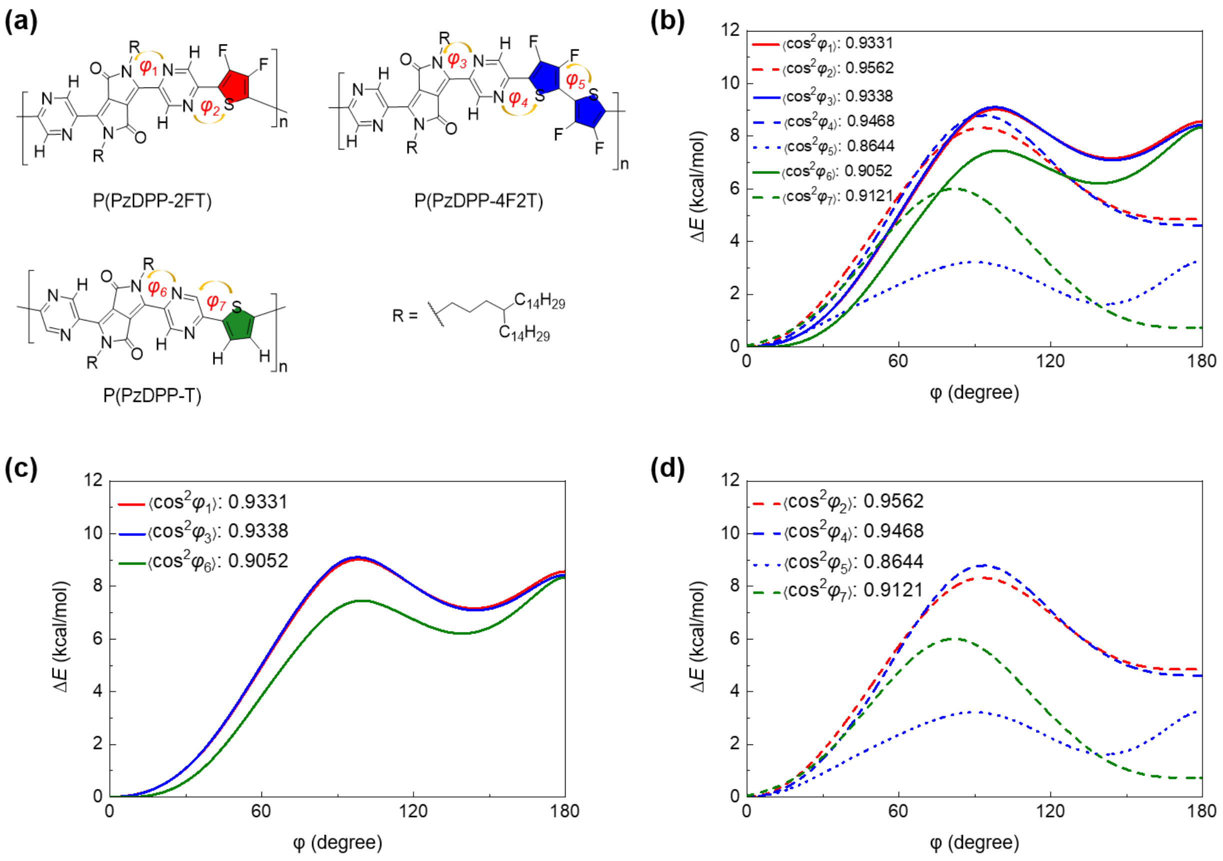

Supplementary Figure 7. (a) Chemical structures of P(PzDPP-2FT), P(PzDPP-4F2T), and P(PzDPP-T). (b) PES results and planarity indexes ⟨$\cos^2\varphi$⟩ of the torsional angles in these three polymers. (c) PES results and planarity indexes ⟨$\cos^2\varphi$⟩ of the torsional angles φ_1, φ_3, and φ_6. (d) PES results and planarity indexes ⟨$\cos^2\varphi$⟩ of the torsional angles φ_2, φ_4, φ_5, and φ_7.

Section 3. DFT-optimized geometries and PES calculation

The geometries of P(PzDPP-2FT), P(PzDPP-4F2T), and P(PzDPP-T) tetramer were optimized at the B3LYP/6-311G(d,p) level with Grimme's D3BJ dispersion using the Gaussian 16 software package[13]. Long alkyl chains were replaced with methyl groups to simplify the calculation. Supplementary Fig. 8 shows that these three polymers all exhibit coplanar backbone conformations under the optimized geometries. In P(PzDPP-2FT), both the dihedral angle between the pyrazine and the DPP core and the dihedral angle between the pyrazine and the 3,4-difluorothiophene (2FT) unit are small ($\varphi_1 = \varphi_2 = 0.002^o$). The O···H and F···H distances in the backbone of P(PzDPP-2FT) are smaller than the sum of the van der Waals radii of O, F, and H (1.52, 1.47, and 1.20 Å, respectively), indicating the existence of the intramolecular hydrogen bonds along the polymer

backbone. Computed torsional potential energy surfaces (PES) were calculated using the ωB97X-D /6-311G(d,p) in the gas phase. For each dihedral, the defined dihedral angle was fixed, and the rest of the repeat unit was allowed to relax.

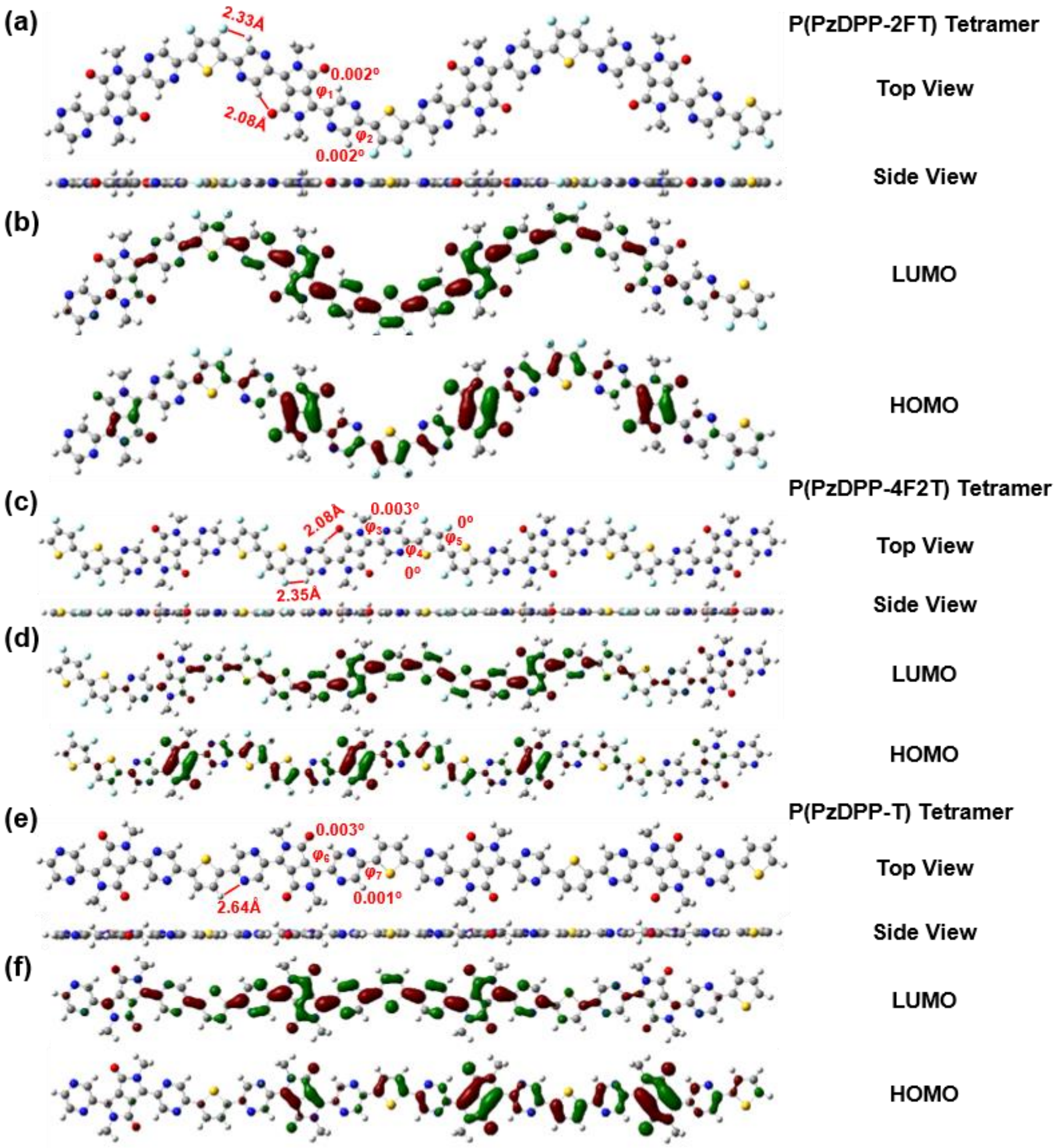

Supplementary Figure 8. DFT-optimized geometries and molecular frontier orbitals of the tetramers of (a) and (b) P(PzDPP-2FT), (c) and (d) P(PzDPP-4F2T), (e) and (f) P(PzDPP-T).

Section 4. Characterization of molecular weights, thermal properties, electrical and optical properties

Molecular weights of the polymers were determined by gel permeation chromatography (GPC) performed on Polymer Laboratories PL-GPC220 at 150 ^{o}C using 1,2,4-tricholorobenzene (TCB) as eluent. Supplementary Fig. 9 shows that both P(PzDPP-2FT) and P(PzDPP-4F2T) exhibited similar molecular weights (M_n) of approximately 53 kDa and polydispersity indexes (PDIs) around 2.1. P(PzDPP-T) exhibited a relatively lower molecular weight (M_n) of 42.2 kDa and PDI of 1.74. These three polymers were carefully purified using solvents extraction, and their GPC curves exhibited near monomodal molecular weight distribution.

Note that the bimodal molecular weight distribution of P(PzDPP-4F2T) and P(PzDPP-T) is a result of their strong aggregation[14]. Bao *et al.* reported a thiophene-diketopyrrolopyrrole based polymer, which shows a bimodal molecular weight distribution when the GPC system is working at a temperature below 180 ^{o}C. The aggregates were finally eliminated when the GPC system temperature increased to 200 ^{o}C, and the GPC results showed a monomodal molecular weight distribution. Moreover, the thiophene-diketopyrrolopyrrole based polymer exhibits almost constant M_n values as the temperature varied from 140 ^{o}C to 200 ^{o}C, suggesting that M_n is good to evaluate the polymers with strong aggregation.

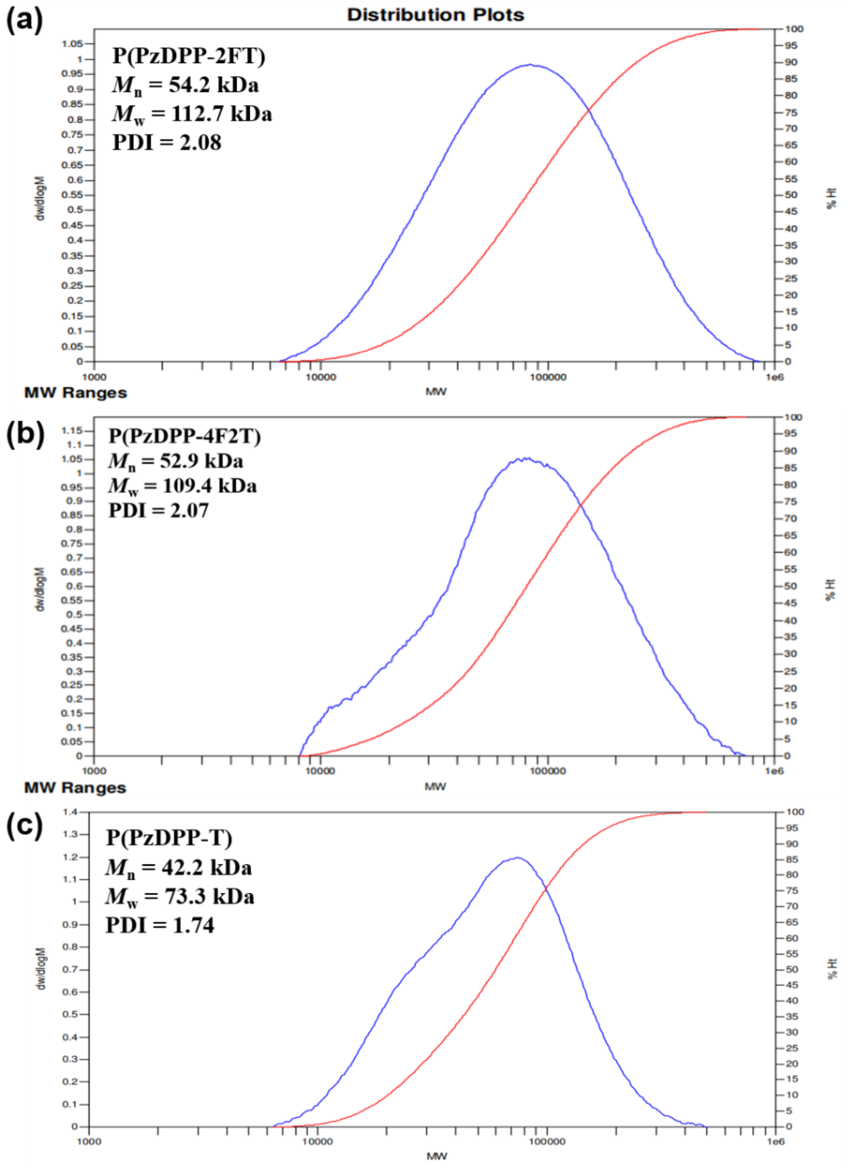

Supplementary Figure 9. Molecular weights and polymer dispersity indexes (PDIs) of (a) P(PzDPP-2FT), (b) P(PzDPP-4F2T) and (c) P(PzDPP-T) measured by high-temperature GPC at 150 °C.

Thermal gravity analyses (TGA) were carried out on a TA Instrument Q600 SDT analyzer, and differential scanning calorimetry (DSC) analyses were performed on a TA Instrument Q2000 analyzer. The three polymers exhibit similar thermal properties with decomposition temperatures above 380 °C with no phase transitions from room temperature to 300 °C.

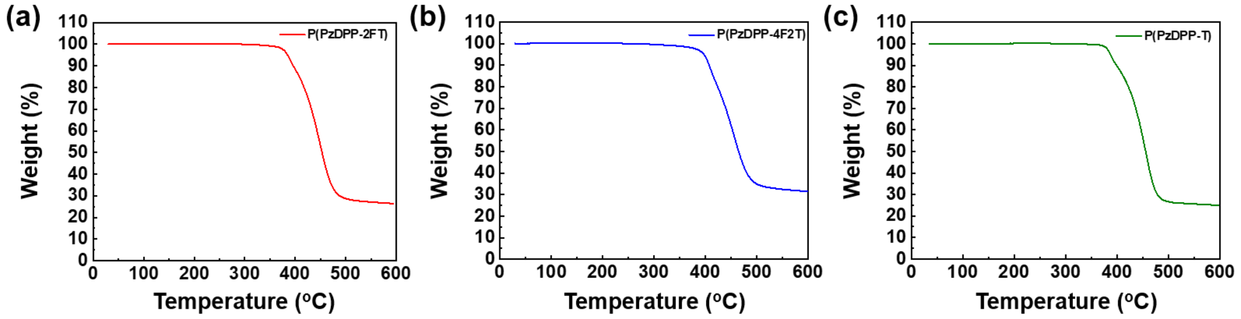

Supplementary Figure 10. Thermal gravity analysis (TGA) of (a) P(PzDPP-2FT), (b) P(PzDPP-4F2T) and (c) P(PzDPP-T).

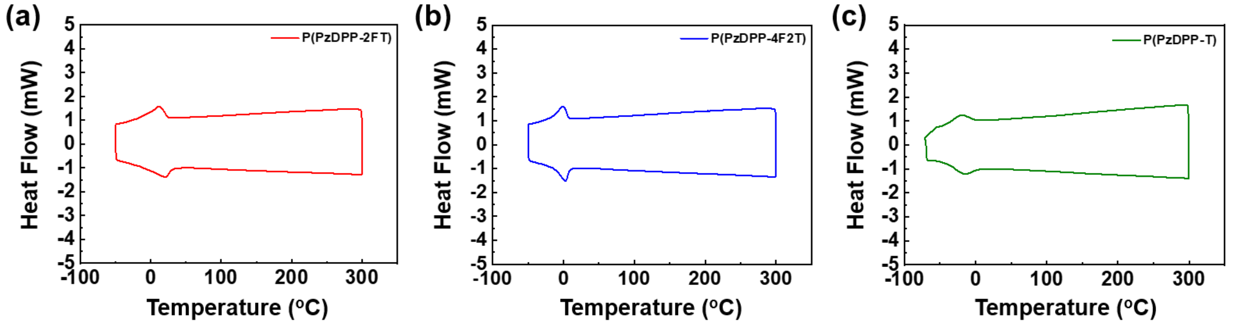

Supplementary Figure 11. Differential scanning calorimeter (DSC) traces of (a) P(PzDPP-2FT), (b) P(PzDPP-4F2T), and (c) P(PzDPP-T) measured at a temperature ramp rate of 10 °C min^{-1} under N_2. Phase transition caused by the melting of the long alkyl side chains were observed (T = 20 °C for P(PzDPP-2FT), T = 3.0 °C for P(PzDPP-4F2T) and T = −15.0 °C for P(PzDPP-T)). A similar side-chain phase transition was also observed in other polymers with similar types of branched alkyl side chains[15,16].

Cyclic voltammetry (CV) was performed on the BioLogic SP-300 workstation. Thin-film measurements were carried out in acetonitrile containing 0.1 M *n*-Bu_4NPF_6 as a supporting electrolyte. A glassy carbon electrode was used as a working electrode and a platinum wire as a counter electrode, and all potentials were recorded versus AgCl/Ag (saturated) as a reference electrode (scan rate: 50 mV s^{-1}). Supplementary Fig. 12 shows that the LUMO (lowest unoccupied molecular orbital) energy level of P(PzDPP-2FT) reaches −3.90 eV, slightly lower than that of P(PzDPP-4F2T) (−3.82 eV) and P(PzDPP-T) (−3.76 eV).

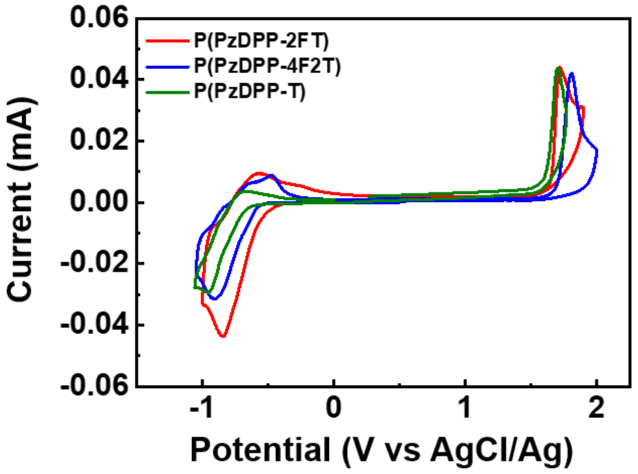

Supplementary Figure 12. Cyclic voltammograms of P(PzDPP-2FT), P(PzDPP-4F2T), and P(PzDPP-T) in thin films. The calculated LUMO energy levels of P(PzDPP-2FT), P(PzDPP-4F2T), and P(PzDPP-T) are −3.90 eV, −3.82 eV, and −3.76 eV, respectively.

Absorption spectra were recorded on PerkinElmer Lambda 750 UV-vis spectrometer. Fluorescence spectra of the solution samples were recorded on Shimadzu RF6000 fluorophotometer. The wavelength of the excitation light is 460 nm. The excitation grating width and emission grating width are both 10 nm. The thin-film absorption spectra of both P(PzDPP-2FT) and P(PzDPP-T) do not exhibit a noticeable shift compared to their solution ones (Supplementary Fig. 13a, c), suggesting that they have rigid and coplanar backbones with similar molecular conformations in both solution and solid state. In contrast, the film absorption spectrum of P(PzDPP-4F2T) exhibited an obvious red-shift compared to the solution (Supplementary Fig. 13b), suggesting that P(PzDPP-4F2T) has a relatively flexible backbone and it may adopt more planar backbone conformation in solid state. Compared to P(PzDPP-4F2T), P(PzDPP-2FT) exhibits smaller FWHM for both its solution absorption and emission peaks (Supplementary Fig. 15). These results indicate that P(PzDPP-2FT) has a more planar and more shape-persistent backbone than P(PzDPP-4F2T). In contrast to the solution absorption, the FWHM for the film 0-0 absorption peak of P(PzDPP-2FT) is 52.26 nm, which is larger than that of P(PzDPP-4F2T) (42.47 nm) (Supplementary Fig. 16). This result can be explained by the fact that the strong π-π stacking interactions in film considerably restrain the torsion angle distributions. Compared with the zigzag configuration of P(PzDPP-2FT), the pseudo-linear backbone of P(PzDPP-4F2T) allows

it to adopt a more ordered molecular packing in film, which considerably restrain the torsion angle distributions in film.

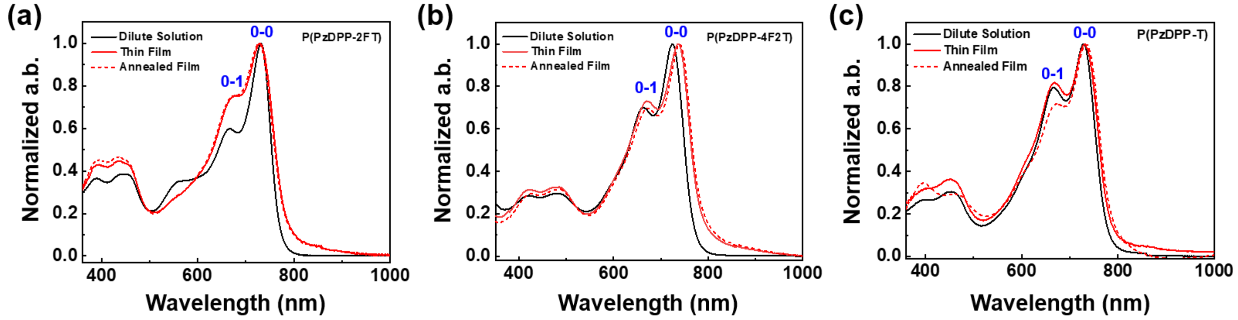

Supplementary Figure 13. Normalized absorption spectra of (a) P(PzDPP-2FT), (b) P(PzDPP-4F2T), and (c) P(PzDPP-T) in solution, thin film, and annealed film.

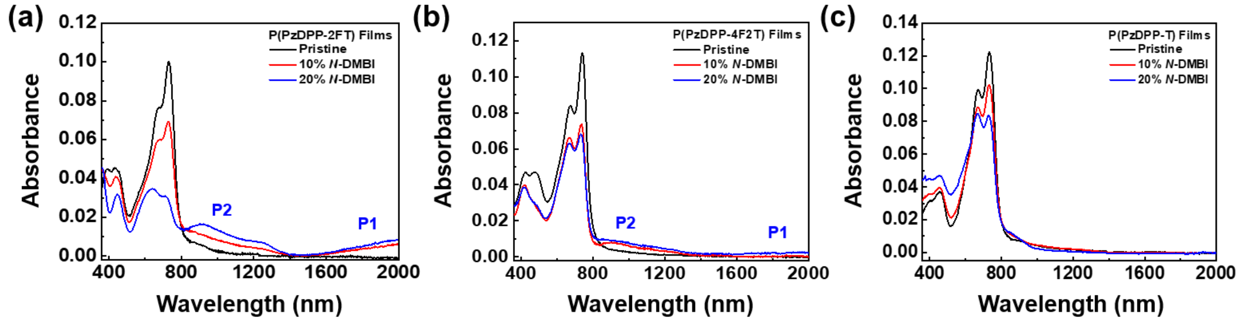

Supplementary Figure 14. Absorption spectra of the pristine and *N*-DMBI doped (a) P(PzDPP-2FT), (b) P(PzDPP-4F2T), and (c) P(PzDPP-T) films.

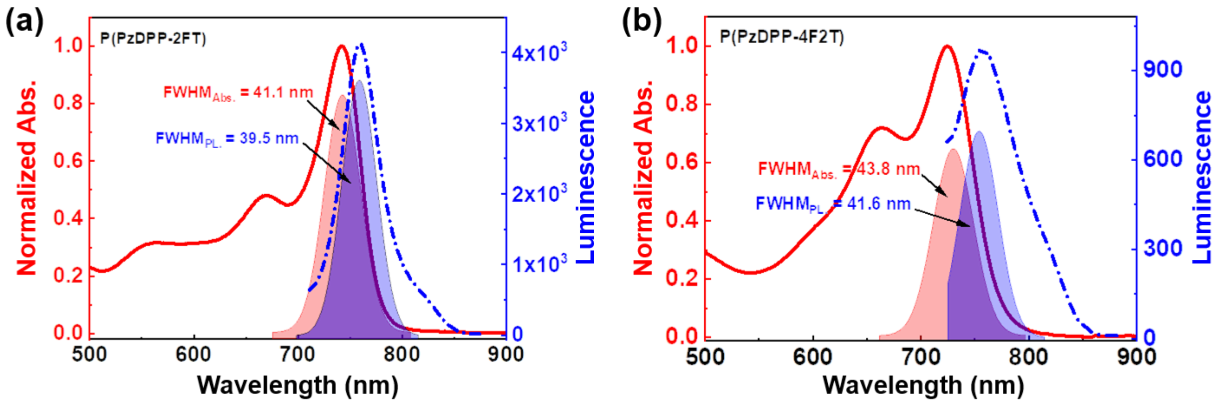

Supplementary Figure 15. Normalized UV-vis absorption spectra and luminescence of (a) P(PzDPP-2FT) and (b) P(PzDPP-4F2T) in CN (1.0×10^{-5} mol/L). The full width at half maxima

of 0−0 absorption peak ($FWHM_{Abs.}$) and the full width at half maxima of the emitting peak ($FWHM_{PL.}$) are obtained through the Gaussian fitting of the 0−0 absorption peak and the corresponding emitting peak.

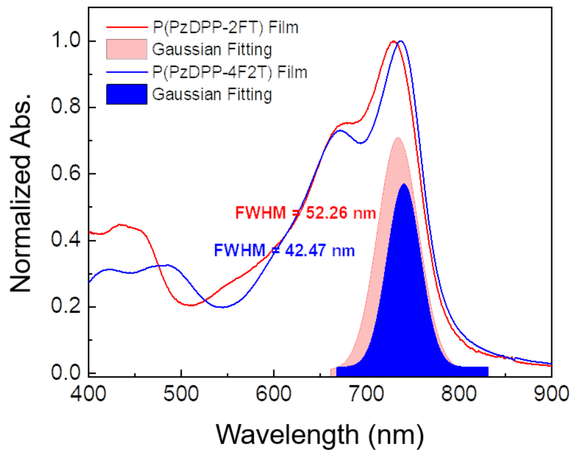

Supplementary Figure 16. Normalized UV-vis absorption spectra of P(PzDPP-2FT) and P(PzDPP-4F2T) in films. The full width at half maxima of 0-0 absorption peak (FWHM) of both polymers are obtained through the Gaussian fitting of the 0-0 absorption peaks.

Section 5. Temperature-dependent conductivity (σ) measurement

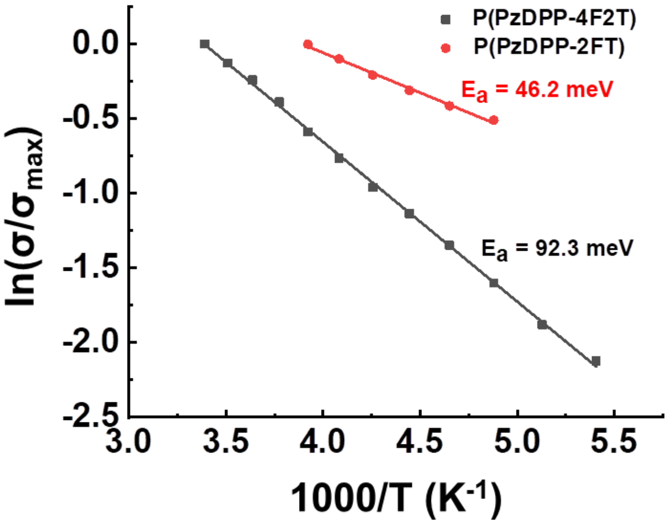

Supplementary Figure 17. Temperature-dependent conductivity (σ) measurement of the *N*-DMBI doped P(PzDPP-2FT) and P(PzDPP-4F2T) film.

As the temperature increasing, the electrical conductivities of the polymers increase. In a hopping transport conducting polymer, assuming that the density of states is constant, the electrical conductivity is temperature dependent:

$$\sigma = \sigma_0 \exp[(\frac{T_0}{T})^s]$$

σ_0 is the pre-exponential factor and T_0 is a characteristic temperature. When 0 < s < 1 is fulfilled, the variable-range hopping mechanism dominates the conduction. The s = 1 contributes to the near-neighbor hopping conduction (NNH) mechanism. In NNH conduction, the carriers with certain activation energy (E_a) hops to the nearest neighbor empty sites. The E_a can be correlated with the conductivity:

$$\sigma = \sigma_0 \exp[-\frac{E_a}{k_B T}]$$

The activation energy was calculated to be 92.3 meV for P(PzDPP-4F2T) and 46.2 meV for P(PzDPP-2FT). The difference in the activation energy implies the lower hopping barriers in the doped P(PzDPP-2FT) films.

Section 6. The doping efficiency comparison of P(PzDPP-2FT) and P(PzDPP-4F2T)

In the XPS characterization, the signal of 400.6 eV and 399.6 eV in the intrinsic film can be attributed to the N(1s) peaks of the amide group in DPP and C=N in pyrazine. When the polymer films were doped with *N*-DMBI, a new N(1s) peak at 402 eV appeared, indicating the generation of *N*-DMBI$^+$. The doping efficiency could be evaluated by the newly formed quadrivalent nitrogen signal of *N*-DMBI$^+$ at 402 eV. At each dopant/polymer ratio, the relative intensity of the cationic *N*-DMBI$^+$ to other N (1s) signals in P(PzDPP-2FT) film is larger than that of P(PzDPP-4F2T) (Supplementary Fig. 18). These results indicate that P(PzDPP-2FT) films exhibit higher doping levels than P(PzDPP-4F2T) in solid state.

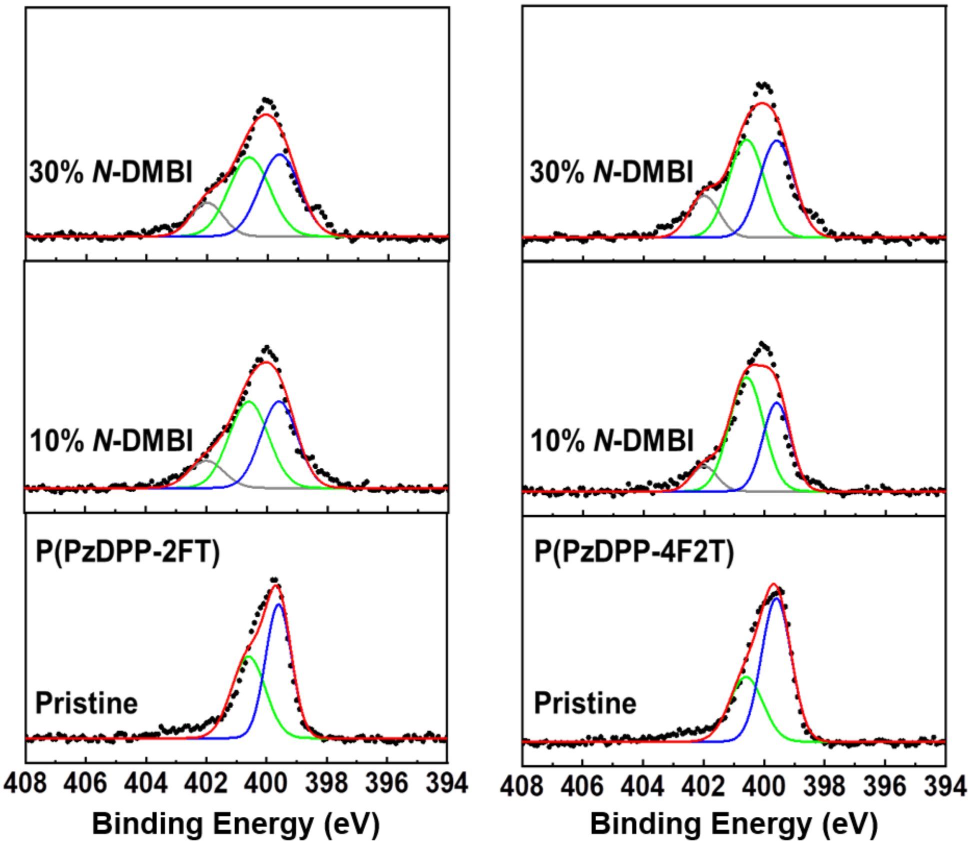

Supplementary Figure 18. XPS data of the pristine and the doped polymers with 10% and 30% *N*-DMBI.

Supplementary Table 1. Calculated electron paramagnetic resonance spectra parameters.

	**Spin counting** [mmol]	**Mass** [mg]	**Spin density** [cm^{-3}]
**P(PzDPP-2FT)**	5.51843E−07	0.13	3.32209E+17
**5% *N*-DMBI**	6.7797E−05	0.29	4.08138E+19
**15% *N*-DMBI**	6.67723E−05	0.94	4.01969E+19
**P(PzDPP-4F2T)**	3.24628E−07	0.16	1.95426E+17
**5% *N*-DMBI**	2.43125E−05	0.85	1.46361E+19
**15% *N*-DMBI**	6.94771E−05	0.50	4.18252E+19

Section 7. AC Hall measurements

The AC-field Hall measurements were carried out in the Lake Shore model 8404 system with an AC field amplitude of 1.2T RMS. The device configuration was designed according to van-der-Pauw geometry. The size of the Hall device is 1 cm × 1 cm.

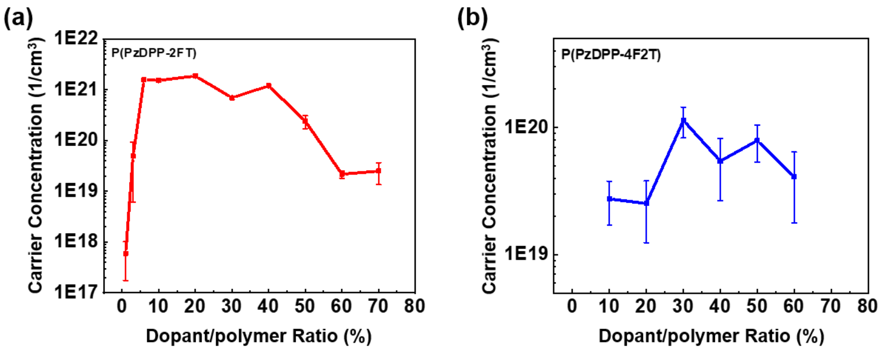

Supplementary Figure 19. Charge carrier concentrations in both *N*-DMBI-doped polymer films evaluated using the AC Hall method. The error bars have been determined as 50% of the experimental value.

Section 8. Microstructure characterization of the pristine and *N*-DMBI doped polymers

GIWAXS experiment was performed on Xenocs Xuess 2.0 beamline, with an incident X-ray angle of 0.2 degrees and wavelength of 1.54 angstrom. The scattered signal was collected by a Pilatus 1M detector at a sample to detector distance of 150 mm. Diffraction data analysis was performed using the Nika software package for Wavemetrics Igor, in combination with WAXS tools.

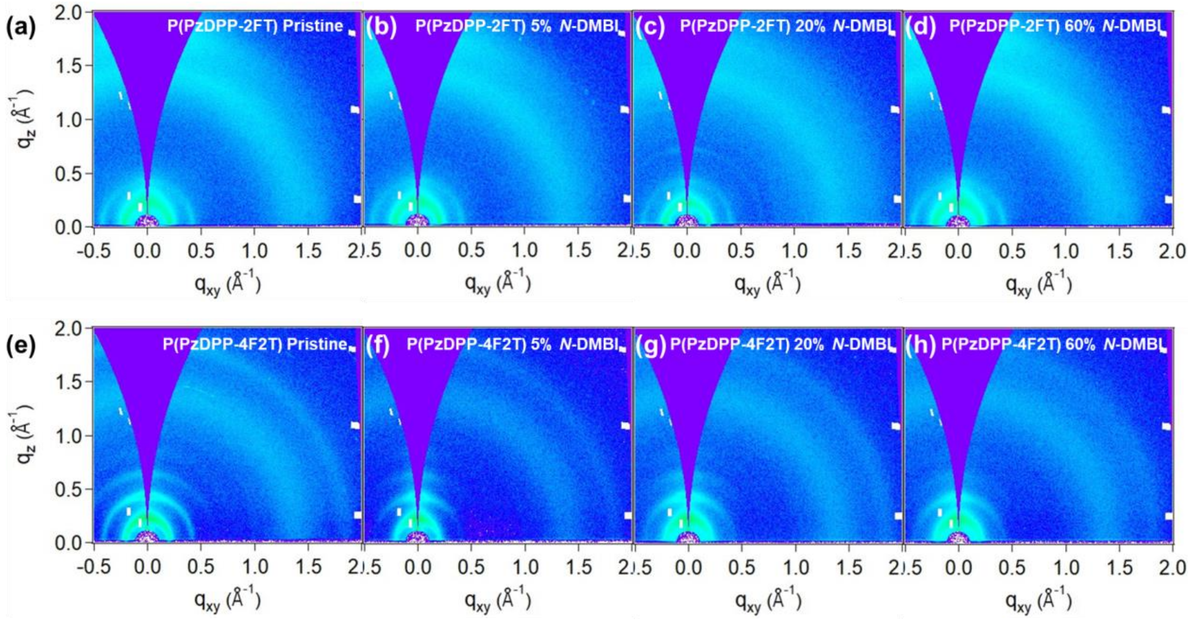

Supplementary Figure 20. (a-d) 2D GIWAXS images of the pristine and the *N*-DMBI doped P(PzDPP-2FT) films. (e-h) 2D GIWAXS images of the pristine and the *N*-DMBI doped P(PzDPP-4F2T) films.

Both pristine polymers exhibit clear (010) diffraction peaks. P(PzDPP-2FT) mainly packs in a face-on orientation, while P(PzDPP-4F2T) exhibits mixed face-on and edge-on orientations. The π-π stacking distances were calculated to be 3.48 Å and 3.37 Å for P(PzDPP-2FT) and P(PzDPP-4F2T), respectively. After *N*-DMBI doping, the lamellar packing and π-π stacking distances of P(PzDPP-4F2T) remain almost unchanged (Supplementary Fig. 21b), while a slight increase of both the lamellar packing and π-π stacking distances was observed for P(PzDPP-2FT) (Supplementary Fig. 21a).

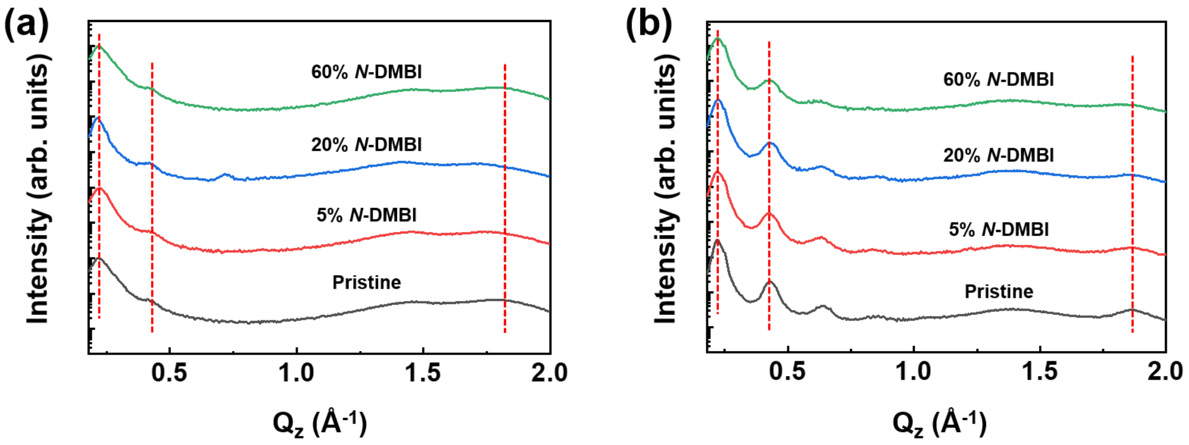

Supplementary Figure 21. Out-of-plane diffractions in GIWAXS analysis of the pristine and *N*-DMBI doped (a) P(PzDPP-2FT) and (b) P(PzDPP-4F2T).

Supplementary Table 2. Calculated lamellar and π-π stacking distances and the FWHM of 100 and 010 from the GIWAXS.

	**Lamellar distance** [Å]	**FWHM of 100** [Å]	**π-π distance** [Å]	**FWHM of 010** [Å]
**P(PzDPP-2FT)**	28.16	106	3.48	26.4
**5% *N*-DMBI**	27.91	111	3.54	25.0
**20% *N*-DMBI**	28.94	112	3.60	24.2
**60% *N*-DMBI**	28.16	103	3.48	27.07
**P(PzDPP-4F2T)**	28.16	157	3.37	53.22
**5% *N*-DMBI**	28.16	131	3.37	40.78
**20% *N*-DMBI**	28.04	137	3.36	45.18
**60% *N*-DMBI**	28.16	117	3.41	33.76

The g values are calculated according to Eq.1

$$g = \sqrt{\frac{\Delta_q}{2\pi q_0}} \quad \text{Eq.1}$$

in which q_0 and Δ_q are the center position and FWHM of a diffraction peak, respectively.

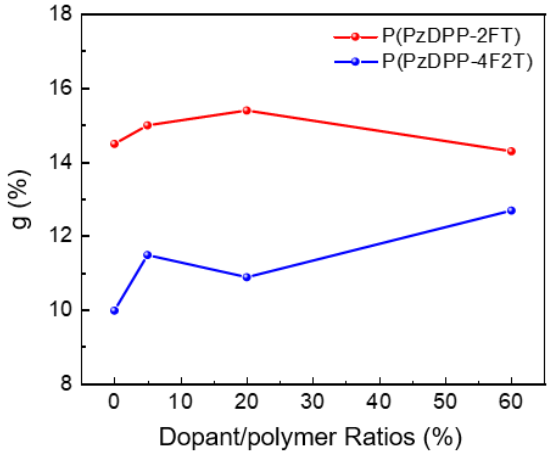

Supplementary Figure 22. Calculated paracrystalline disorder of 010 diffractions of P(PzDPP-2FT) and P(PzDPP-4F2T) based on GIWAXS.

Atomic force microscopy (AFM) measurements were performed with a Cypher atomic force microscope (Asylum Research, Oxford Instruments). The surface morphology and film thickness were recorded with a scan rate of 2.44 Hz at AC mode.

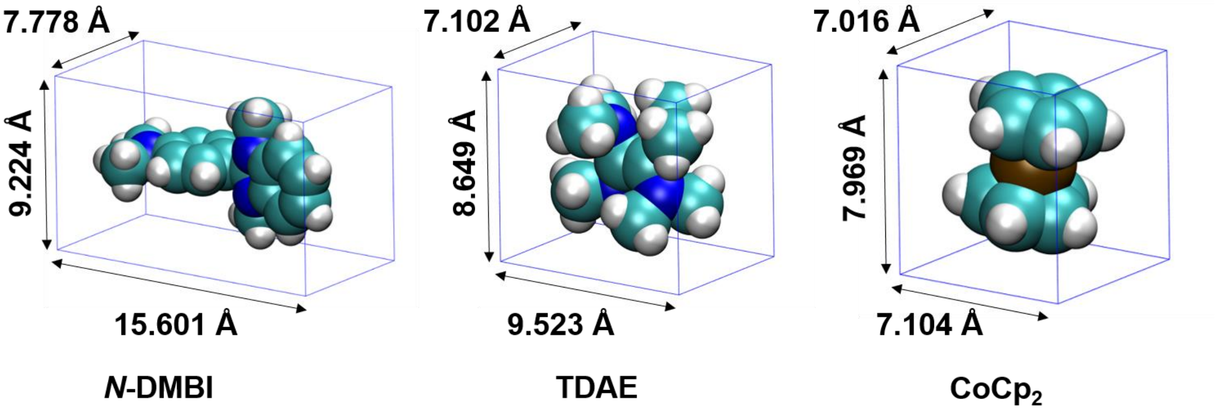

Supplementary Figure 23. The calculated molecular size of the three dopants *N*-DMBI, TDAE, and $CoCp_2$. Compared to *N*-DMBI, $CoCp_2$ and TDAE have smaller molecular sizes.

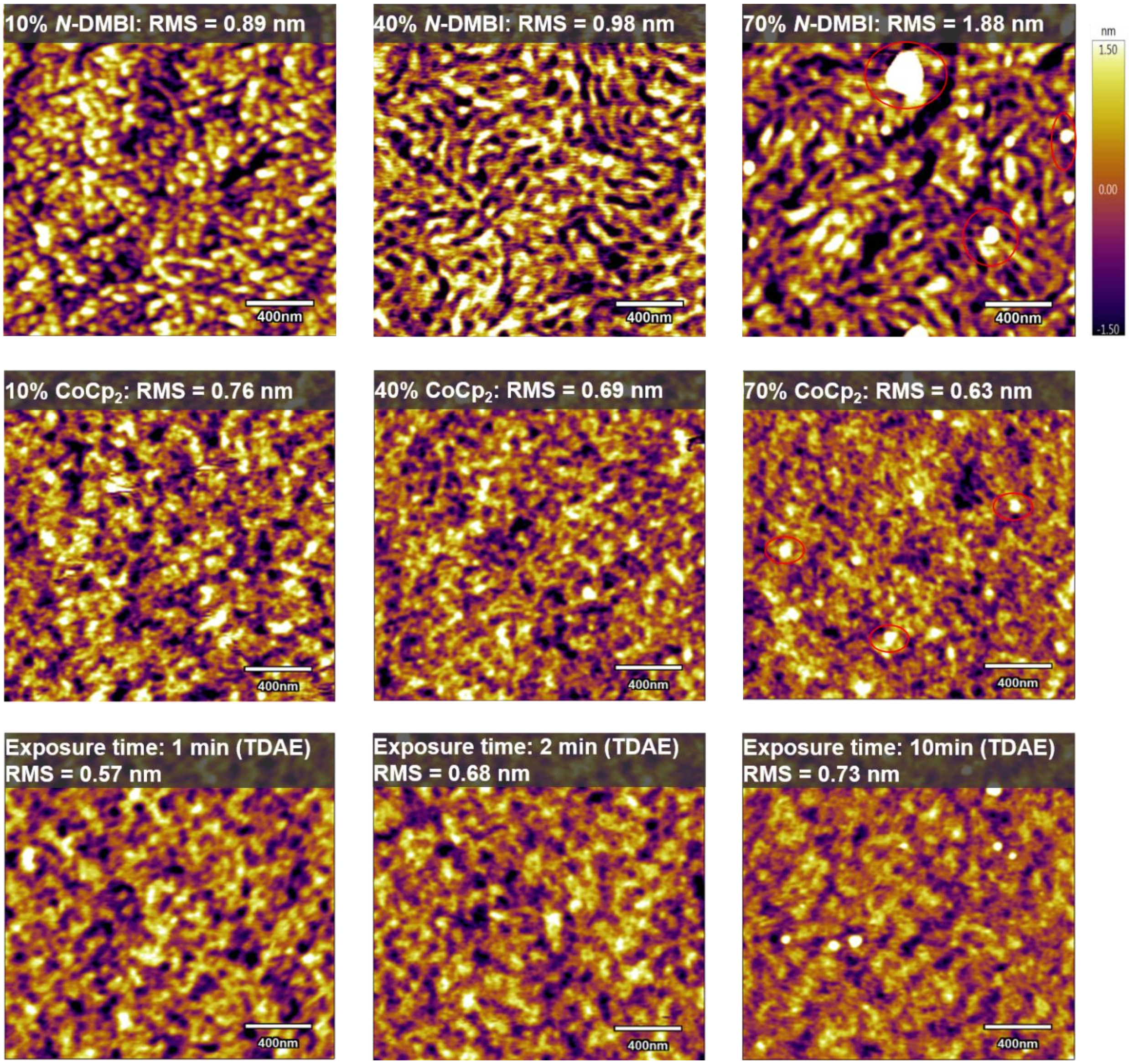

Supplementary Figure 24. AFM height images of P(PzDPP-4F2T) polymer films doped with *N*-DMBI, $CoCp_2$, and TDAE at various doping concentrations. *N*-DMBI doped films showed rougher surfaces and exhibited obvious phase separation and large aggregates at high doping concentrations (70%). $CoCp_2$ and TDAE doped films exhibited more uniform and smoother surfaces with a few small aggregates at high doping concentrations. Therefore, $CoCp_2$ and TDAE showed better miscibility with the polymer matrix. The large aggregates could damage the film microstructures and charge transport pathways, thus leading to decreased conductivity.

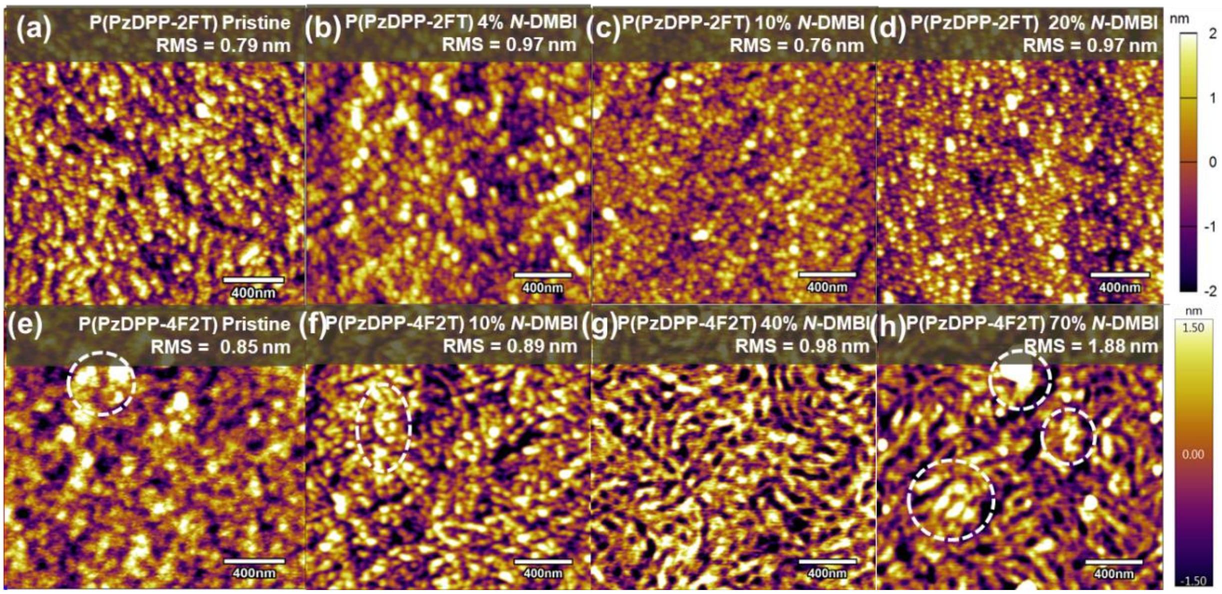

Supplementary Figure 25. AFM height images of the pristine and the *N*-DMBI doped films of both polymers: (a-d) P(PzDPP-2FT) and (e-h) P(PzDPP-4F2T).

Section 9. AFM-IR characterization

The infrared absorption spectra were recorded on Bruker Tensor 27 FTIR spectrometer. The sample preparation is shown below: The solutions of all samples were drop-cast on KBr salt tablets and dried under vacuum. Another KBr salt tablet was used to cover the surface of the sample, and the edge of the salt tablets was sealed with parafilm. These operations were carried out in a glove box under a nitrogen atmosphere. Supplementary Fig. 26 shows that *N*-DMBI has a unique IR absorption at 1524 cm^{-1}, and the *N*-DMBI doped P(PzDPP-2FT) film has very weak IR absorption at the same wavenumber. On the other hand, the *N*-DMBI doped P(PzDPP-2FT) film exhibits intense IR absorption at 1672 cm^{-1}, while the *N*-DMBI has no IR absorption at this wavenumber. In the AFM-IR characterization, both the doped samples were measured at both unique wavenumbers for the dopant and the doped polymers (1524 cm^{-1} and 1672 cm^{-1}).

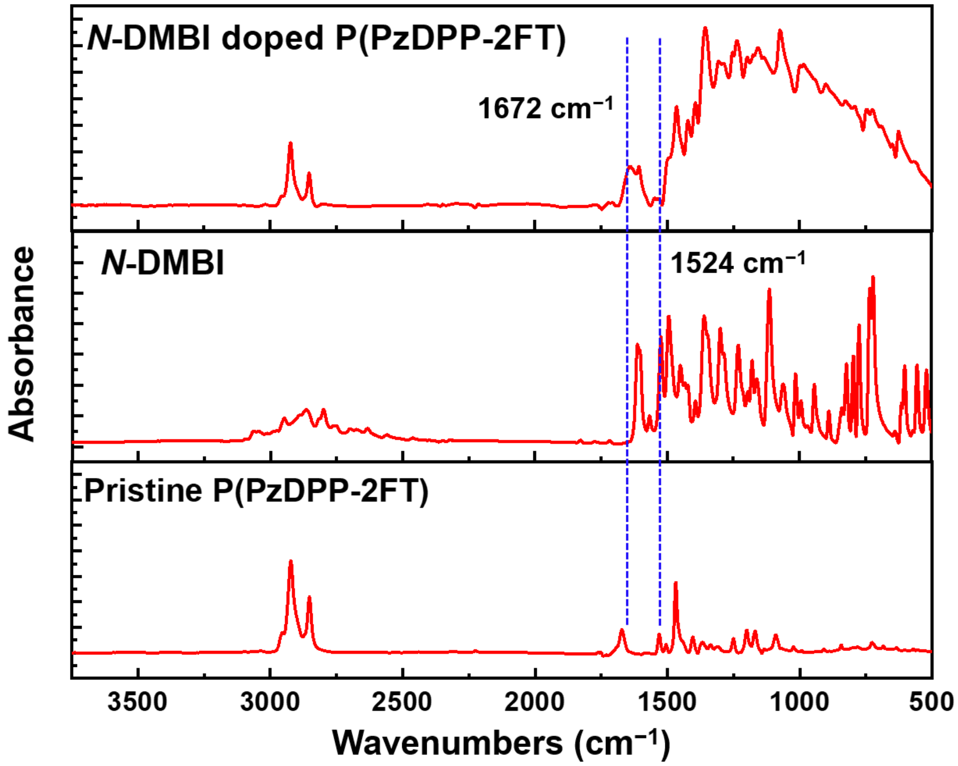

Supplementary Figure 26. Infrared absorption spectra of the *N*-DMBI doped P(PzDPP-2FT) (top), the dopant *N*-DMBI (middle), and the pristine P(PzDPP-2FT) (bottom) films.

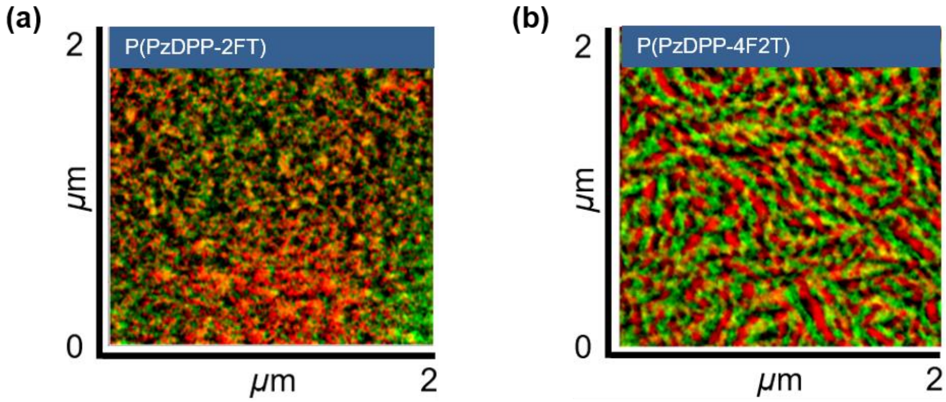

Supplementary Figure 27. AFM-IR maps of 30% *N*-DMBI doped (a) P(PzDPP-2FT) and (b) P(PzDPP-4F2T) films. The *N*-DMBI has a unique IR peak at 1524 cm^{-1} (green area), and the doped polymers have a unique IR peak at 1672 cm^{-1} (red area).

Section 10. Solubility evaluation for P(PzDPP-2FT) and P(PzDPP-4F2T).

The standard solutions of both polymers with different concentrations were prepared by dissolving 3 mg polymer in 1 mL CN at 100 ^{o}C, and then diluted from this solution into different proportions after it was cooled to room temperature. The saturated solutions of both polymers were prepared by dissolving 4 mg polymer in 0.3 mL CN at 100 ^{o}C. These solutions were cooled to room temperature and aged for 2 h. We observed that there were small particles absorbed on the bottle wall, indicating the formation of saturated solutions. These solution mixtures were filtered with a 0.45 μm filter membrane and then diluted 60 μL of these filtrates to 5 mL solutions. Then the solutions were diluted 10 times, and their concentrations were calibrated by the UV-vis absorption spectra. For P(PzDPP-2FT), the linear function $A = -0.00853 + 38.37889C$ was fitted according to the linear relationship between the absorption value at 743 nm (A) and the solution concentrations (C). The solution diluted from the saturated solution exhibited an absorption value of 0.528 at 743 nm, and the calculated concentration was 0.0140 mg mL^{-1}. Then the concentration of the original saturated solution was 833.3×0.0140 mg mL^{-1} = 11.67 mg mL^{-1}. For P(PzDPP-4F2T), the linear function $A = -0.0119 + 40.28934C$ was fitted according to the linear relationship between the absorption value at 724 nm (A) and the solution concentrations (C). The solution diluted from the saturated solution exhibited an absorption value of 0.524 at 724 nm, and the calculated concentration was 0.0133 mg mL^{-1}. Then the concentration of the original saturated solution was 833.3×0.0133 mg mL^{-1} = 11.08 mg mL^{-1}.

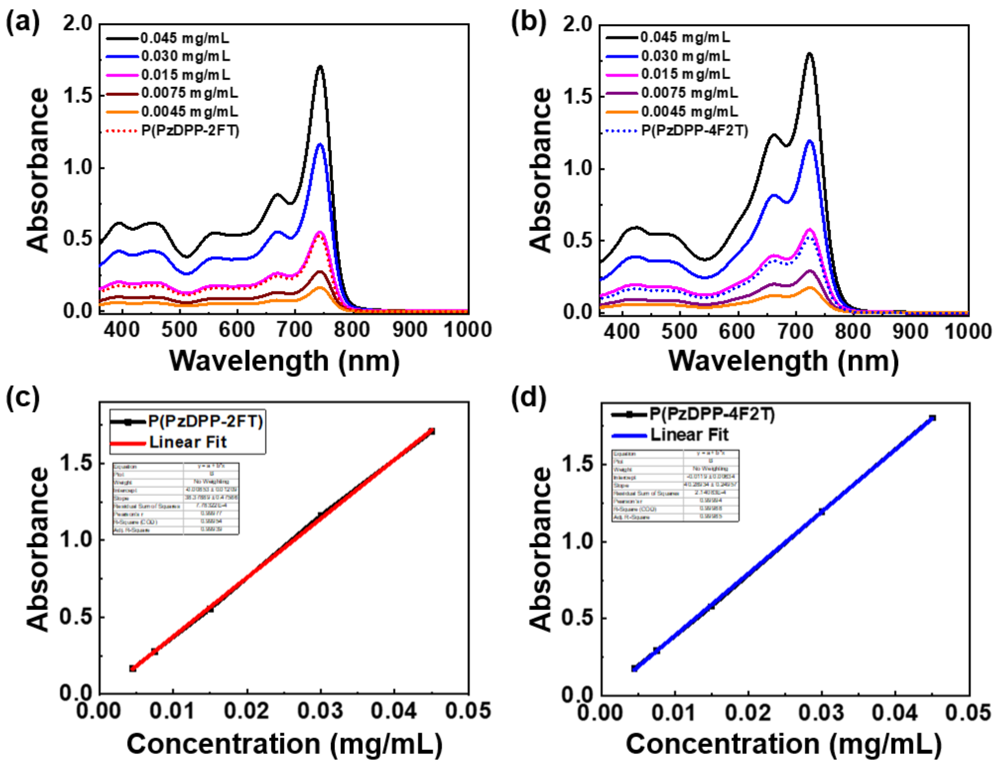

Supplementary Figure 28. Solubility comparison of P(PzDPP-2FT) and P(PzDPP-4F2T) in CN solutions. (a) UV-vis absorption spectra of P(PzDPP-2FT) solutions with different concentrations in CN (solid lines) and the P(PzDPP-2FT) solution diluted from saturated CN solution (dash line). (b) UV-vis absorption spectra of P(PzDPP-4F2T) solutions with different concentrations in CN (solid lines) and the P(PzDPP-4F2T) solution diluted from saturated CN solution (dash line). (c) The linear relationship between the absorption value at 743 nm and the solution concentrations, as well as the linear fitting according to this relationship. (d) The linear relationship between the absorption value at 724 nm and the solution concentrations, as well as the linear fitting according to this relationship.

Section 11. Field-effect transistor (FET) devices fabrication and characterization

TG-BC FET devices fabricated on the Si-SiO_2 substrates. The source and drain electrodes (Ti/Au) were patterned by photolithography. The substrates were cleaned by using ultrasonication in acetone, detergent, deionized water (three times), and isopropyl alcohol. The substrates were dried by nitrogen flow and cleaned by plasma. The polymer films were deposited on the cleaned substrates by spin-coating the polymer solution at 1800 rpm for 60 s and then annealed at 150 ^{o}C for 10 min. After depositing the polymer film, a CYTOP solution (CTL809M/CT-solv 180 = 3/1)

was spin-coated onto the polymer film at 2000 rpm for 60 s and annealed at 200 ^{o}C for 1 h in a glovebox. Al (45 nm) was evaporated through a shadow mask onto the dielectric layer by thermal evaporation as the gate electrode. The characterization of FETs was carried out under ambient conditions on the probe stage using Keithley 4200 SCS. The mobility is calculated by the saturated regime according to the equation $I_{SD} = (W/L)C_i\mu(V_G - V_T)^2$. W and L are the device channel width and length (W = 100 μm, L = 5 μm). C_i (C_i = 3.7 nF cm^{-2}) is the capacitance per unit area of the gate dielectric layer. V_G and V_T are the gate voltage and the threshold voltage.

FET characterization shows that the pristine P(PzDPP-2FT) with higher packing disorder exhibited comparable electron mobilities (1.30 $\pm$ 0.14 cm^2 V^{-1} s^{-1}) with that of P(PzDPP-4F2T) (1.28 $\pm$ 0.25 cm^2 V^{-1} s^{-1}). P(PzDPP-T) exhibited low electron mobility of 0.001 cm^2 V^{-1} s^{-1}.

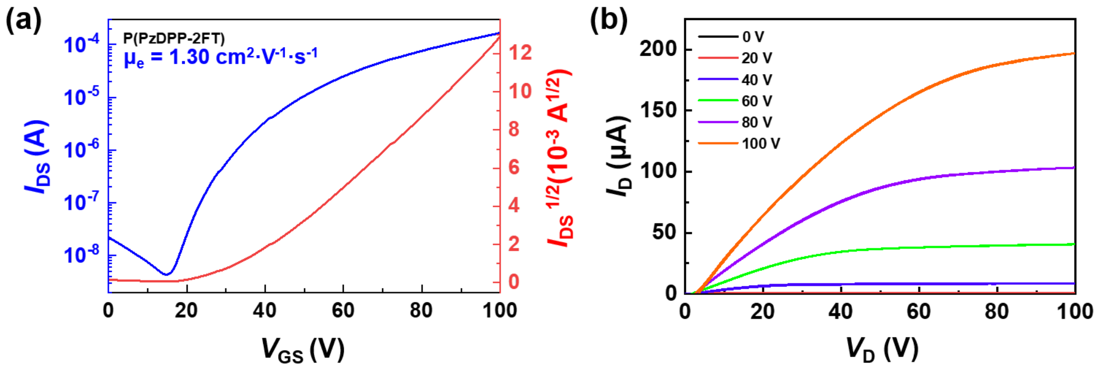

Supplementary Figure 29. (a) Transfer and (b) output characteristics for the pristine polymer P(PzDPP-2FT).

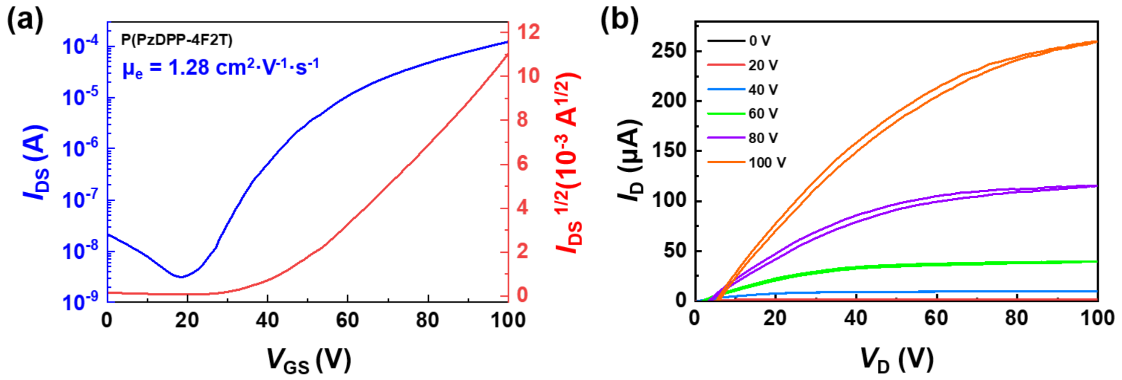

Supplementary Figure 30. (a) Transfer and (b) output characteristics for the pristine P(PzDPP-4F2T) film.

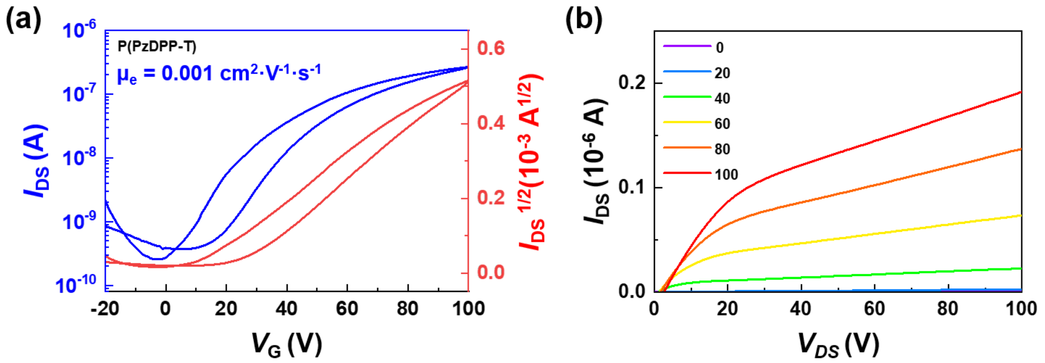

Supplementary Figure 31. (a) Transfer and (b) output characteristics for the pristine P(PzDPP-T) film.

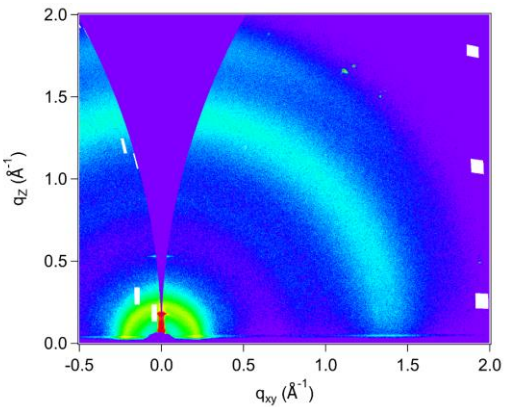

Supplementary Figure 32. 2D GIWAXS images of the pristine P(PzDPP-T) films.

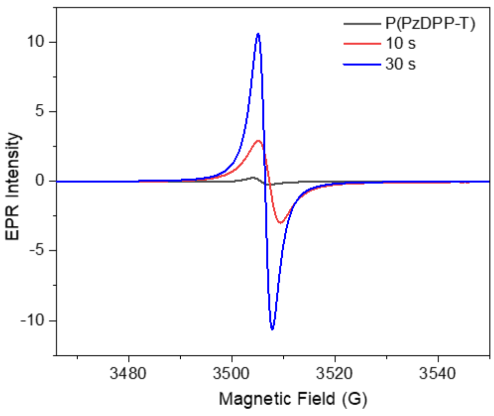

Supplementary Figure 33. EPR signals of the pristine and the TDAE doped P(PzDPP-T) films under different exposure times.

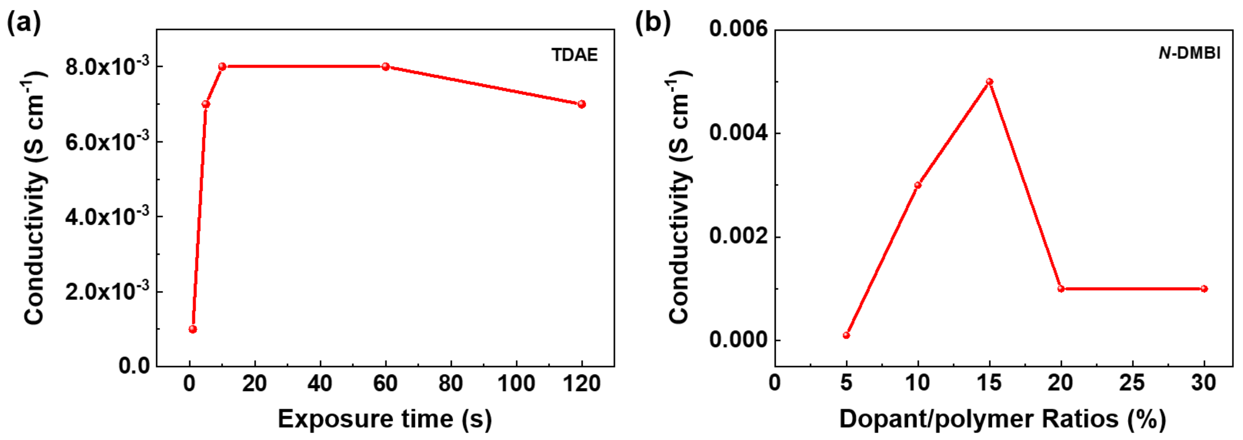

Supplementary Figure 34. (a) The conductivities of P(PzDPP-T) films doped by TDAE. (b) Electrical conductivities of doped P(PzDPP-T) films at different *N*-DMBI/polymer ratios.

Due to the low charge carrier mobility of P(PzDPP-T), it is difficult to obtain its charge carrier concentration by Hall effect measurements. After several trials, we could not obtain reliable data for P(PzDPP-T). Therefore, EPR measurement was used to evaluate the charge carrier concentration of the polymer films doped by TDAE. EPR shows that P(PzDPP-T) can be effectively doped by TDAE, and the spin density is approximately 10^{19} cm^{-3} (Supplementary Fig. 33). The charge carrier concentration of doped P(PzDPP-T) films is comparable to the doped P(PzDPP-2FT) (4.08×10^{19} cm^{-3} when doped with 5% *N*-DMBI) and P(PzDPP-4F2T) (1.46×10^{19} cm^{-3} when doped with 5% *N*-DMBI) films (Supplementary Table 1). As we know, the spin density measured by EPR only reflects the number of polarons, and the bipolarons are silent in EPR. Thus, EPR provides a lower bound for the charge carrier concentration (n). Considering the n for P(PzDPP-T) is 1×10^{19} cm^{-3} and the n for P(PzDPP-2FT) is 1×10^{21} cm^{-3} (Hall) or 4×10^{19} cm^{-3} (EPR), the n of P(PzDPP-2FT) is at most two orders of magnitude higher than that of P(PzDPP-T), which cannot explain the four orders of magnitude higher electrical conductivity of P(PzDPP-2FT). Therefore, the low electrical conductivity of the doped P(PzDPP-T) film is attributed to its intrinsically large structural and energetic disorder and low charge carrier mobility.

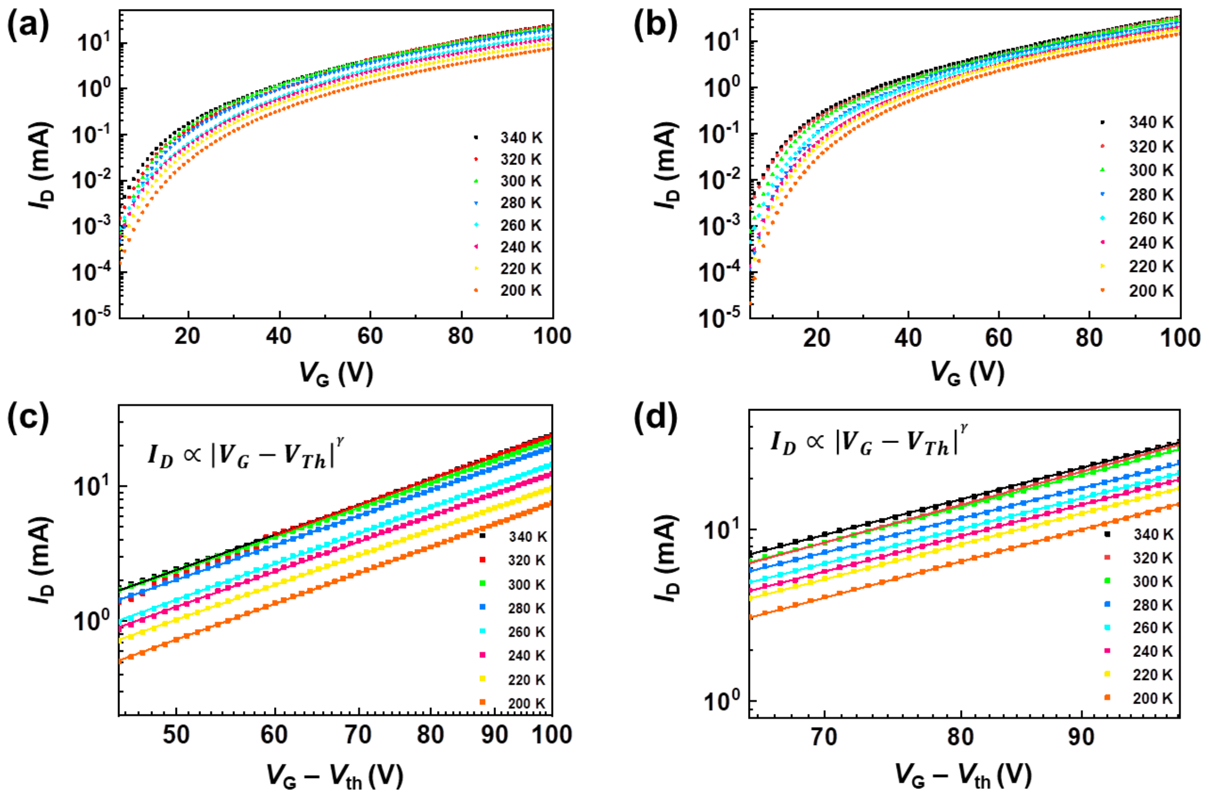

Supplementary Figure 35. Temperature evolution of (a) P(PzDPP-2FT) and (b) P(PzDPP-4F2T) transfer curves; Replotted curves of the temperature-dependent transfer characteristics on a double logarithmic scale for (c) P(PzDPP-2FT) and (d) P(PzDPP-4F2T). Solid lines are fitted to extract the parameter γ for each temperature.

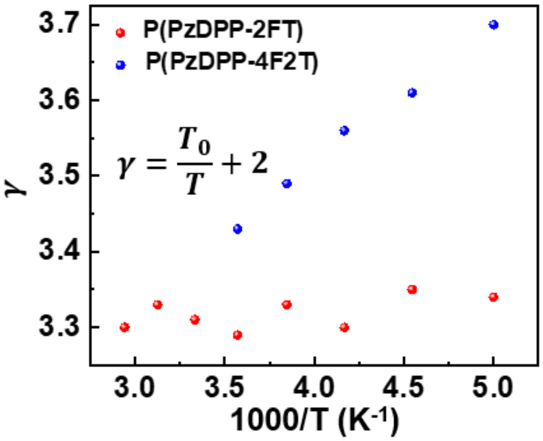

Supplementary Figure 36. Extracted γ values versus 1/T for both polymers.

For both polymers, the temperature evaluation of the transfer curves can be fitted using the disorder FET model[17]. The data was collected in the temperature range of 200 K ~ 340 K. The model can be described as:

$$I_D^{2D} = A\frac{W}{L}d_{sc}^{1-\left(\frac{T_0}{T}\right)}\left(\frac{C_i}{e}\right)^{\frac{T_0}{T}}\frac{T}{T_0 - T}(V_G - V_t)^{\left(\frac{T_0}{T}\right)+1}$$

In the above expression, T_0 is the characteristic width of the exponential DOS. The parameter d_{sc} is the thickness of the slab in the semiconductor. In the saturation regime, the data can be adjusted by $I_D \propto (V_G - V_{Th})^{\gamma}$. The exponent γ reflects the temperature dependence of the charge transport property. For P(PzDPP-2FT), the exponent γ takes a temperature-independent value 3.3, while the exponent γ decreases with increasing the temperature for P(PzDPP-4F2T). These results suggest a higher energetic disorder in P(PzDPP-4F2T).

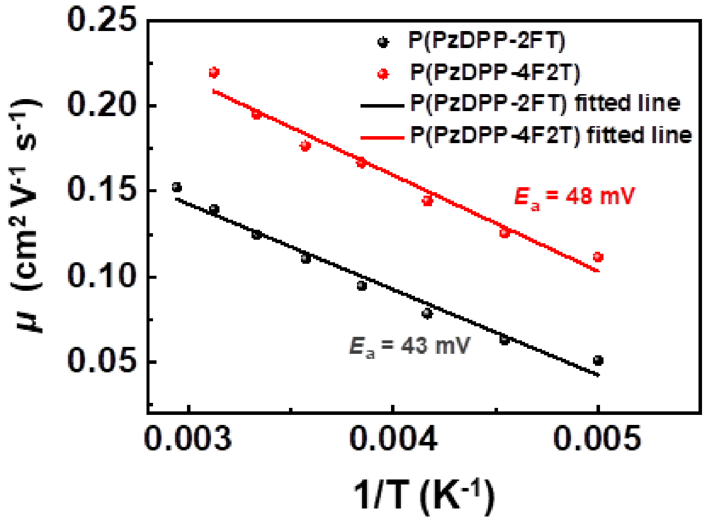

Supplementary Figure 37. Temperature-dependent charge carrier mobilities for both polymers.

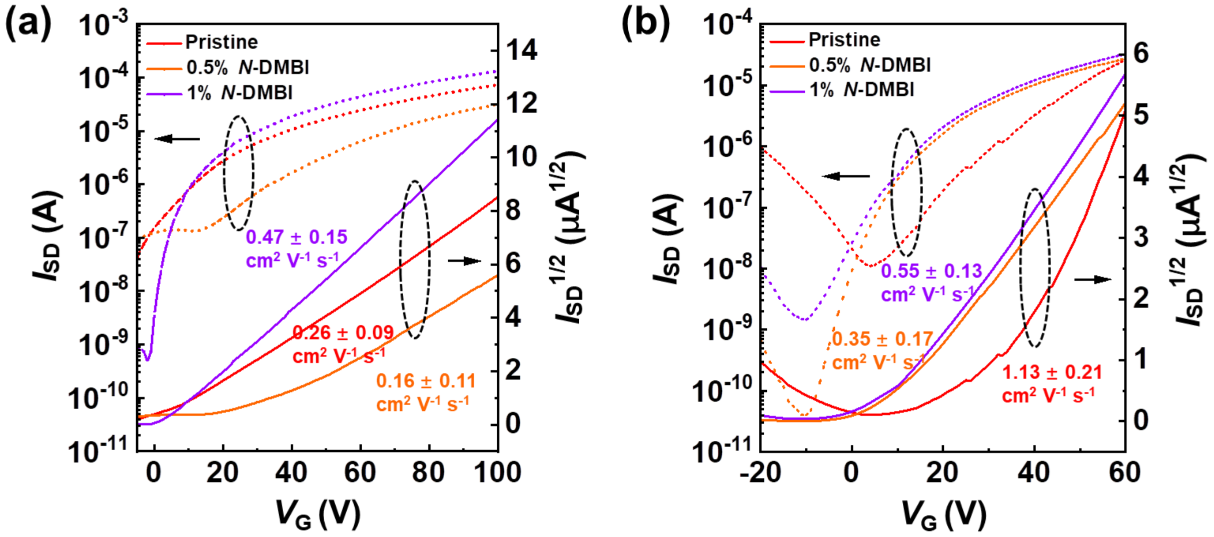

Supplementary Figure 38. Transfer characteristics and calculated electron mobilities of the FET devices of the pristine, 0.5%, and 1% *N*-DMBI-doped (a) P(PzDPP-2FT) and (b) P(PzDPP-4F2T) films.

Section 12. Photothermal deflection spectroscopy (PDS)

The PDS measurements were carried out with a 1 kW Xe arc lamp and a 1/4 m grating monochromator (Oriel) as the tunable light source. The pump beam was modulated at 13 Hz using a mechanical chopper before irradiating the sample. Perfluorohexane was used as the deflection fluid. A Uniphase He-Ne laser was directed parallel to the sample surface as the probe laser. A quadrant cell (United Detector Technology) was used as the position sensor for monitoring the photothermal deflection signal of the probe beam. The output of the detector was fed into a lock-in amplifier (Stanford Research, Model SR830) for phase-sensitive measurements. All PDS spectra were normalized to the incident power of the pump beam.

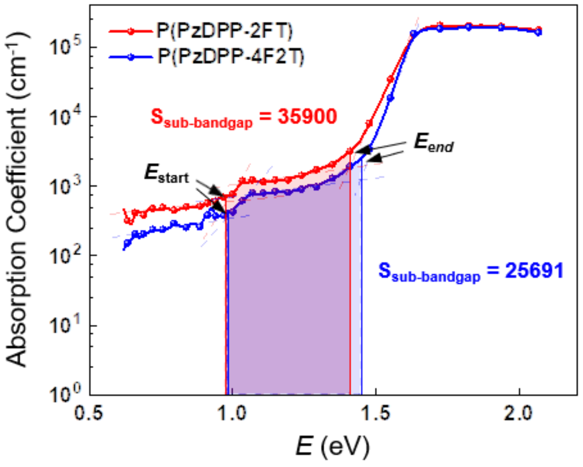

Supplementary Figure 39. PDS spectra of spin-coated P(PzDPP-2FT) and P(PzDPP-4F2T) thin films. The starting and ending points of the sub-bandgap shoulder area are identified through the intersection points of the absorption spectrum fitting of adjacent regions, namely the E_{start} and the E_{end} within the plots. All the y axis values (absorption coefficients) within the colored region are summed up, representing the area of sub-bandgap ($S_{\text{sub-bandgap}}$) of the corresponding polymer film.

Section 13. Temperature-dependent electrical conductivities.

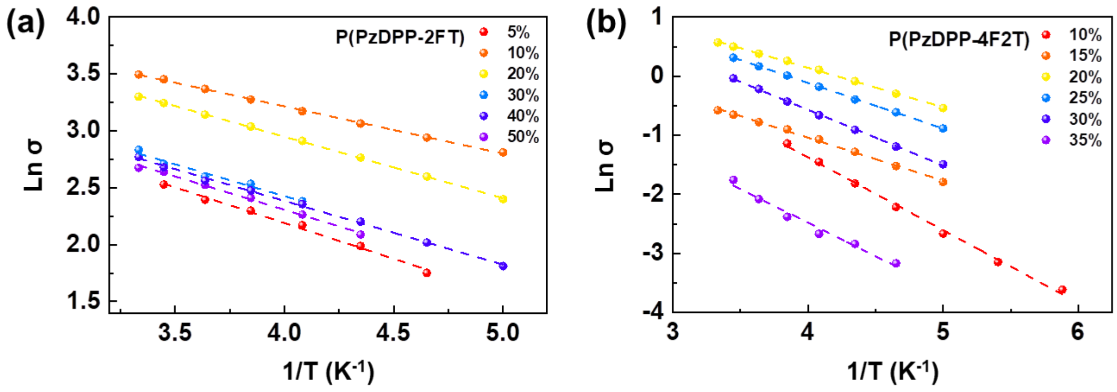

Supplementary Figure 40. The variable temperature-dependent conductivities of (a) P(PzDPP-2FT) and (b) P(PzDPP-4F2T) at different doping concentrations.

Section 14. Molecular dynamics calculations

Based on the quantum-chemical calculations, a force field derived from the Dreiding force field was used, in which the torsion potentials between adjacent conjugated units have been re-parameterized against ωB97X-D/6-311G(d,p) calculation. DFT optimized geometries of the

dimers were used as initial structures, and atomic charges were resigned according to DFT calculated ESP charges. We used the methodologies according to the literature to simulate the crystalline and disorder region of P(PzDPP-2FT), P(PzDPP-4F2T), and P(PzDPP-T)[3]. All molecular dynamics simulations were performed using Material Studio[18], and all DFT calculations were performed with Gaussian 16 package[13].

To search for the most stable molecular structures, we performed 4 thermodynamics steps: (i) unit cells with different cell parameters (according to XRD results), containing one chain made of 2 monomers, were built under periodic boundary conditions to simulate an infinite system. (ii) both molecular systems were optimized and thereafter quenched at the molecular mechanics level (NPT, T = 300 K, quench frequency = 1 ps, simulation time = 100 ps) until the energy between two consecutive structures no longer decreases; (iii) the continued 100 ps quenched dynamics at higher temperatures (600 K and 1000 K) are performed for more stable structures; (iv) molecular structures obtained at step iii are quenched with longer simulation time (500 ps) at increasing temperatures (300 K, 600 K, and 1000 K).

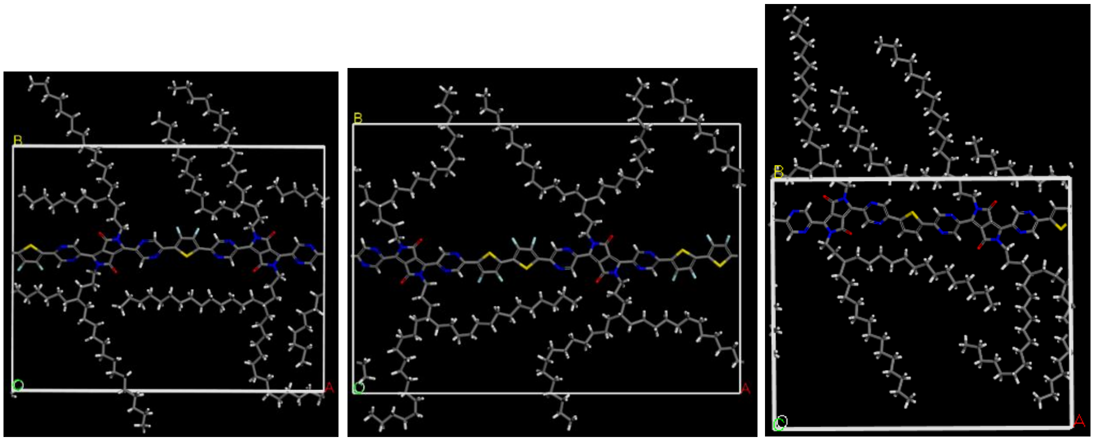

Supplementary Figure 41. Examples of the most stable unit cell of P(PzDPP-2FT) (left), P(PzDPP-4F2T) (middle), and P(PzDPP-T) (right). The cell parameters are a = 35.79 Å, b = 31.74 Å, c = 3.80 Å for P(PzDPP-2FT), a = 38.97 Å, b = 30.66 Å, c = 3.79 Å for P(PzDPP-4F2T), and a = 36.95 Å, b = 31.08 Å, c = 3.86 Å.

The steric effects of bulk alky side-chains greatly impede the molecular packing when constructing the unit cell with XRD cell parameters. To search possible molecular packing modes in crystalline, we build initial unit structures with index a as the dimer length and c as π-π stacking distance while keeping the lamella distance large enough (~ 50 Å) to contain almost linear alkyl chains. Gradually decrease the b value to around 28 Å by multiple geometry optimizations,

possible structures with appropriately relaxed alky chains are obtained as shown in Supplementary Figure 41. Unsurprisingly, the search procedures for the possible molecular packing modes of P(PzDPP-2FT) and P(PzDPP-T) in crystalline are far more challenging than that of P(PzDPP-4F2T). Normally, it only takes a few optimization steps for P(PzDPP-4F2T) to find the local most stable structures, while more steps are needed for the other two rigid polymers. In addition, P(PzDPP-4F2T) unit cell, consisting of more atoms, is thought to have higher energy. However, we find that after optimization, P(PzDPP-4F2T) accesses to lower energy with more significant enthalpy reduction, which also indicates that the crystalline phase of P(PzDPP-4F2T) is easier to access and is more stable. This result is consistent with our experimental results that the crystallinity of P(PzDPP-2FT) is much lower than P(PzDPP-4F2T), which suggests that P(PzDPP-2FT) has more disorder regions and interchain spacing for dopants diffusion and storage.

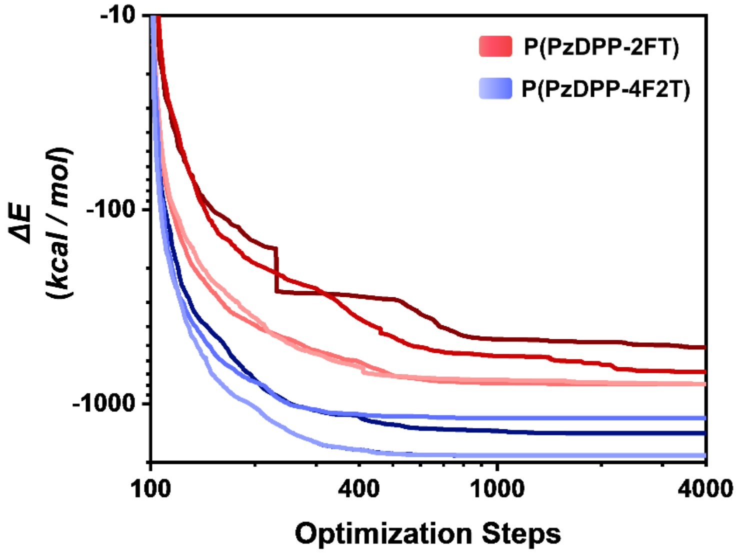

Supplementary Figure 42. Analysis of the system enthalpy change (ΔE) during structure optimization. Different lines in red or blue represent different starting structures of P(PzDPP-2FT) and P(PzDPP-4F2T), respectively.

To simulate the crystalline structure of these polymers, we constructed supercells made of 3 layers of 8 π-stacked of dodecamers. All supercells have been thermalized and stabilized with two consecutive molecular dynamics: (i) Each supercell is thermalized and relaxed with the first molecular dynamics (NPT, P = 1 atm, T = 298 K, 200 ps); (ii) The second step is performed to collect data (NPT, P = 1 atm, T = 298 K, 200 ps for 201 snapshots).

To further compare the different behaviors of P(PzDPP-2FT), P(PzDPP-4F2T), and P(PzDPP-T) in less dense structures, we generated the disorder region of the three polymers via

four procedures: (i) Based on the most stable unit structure, supercells with three layers of 8 π-stacked of dodecamers were built; (ii) The π-π stacking confinement was increased from 3.48 Å to 5.00 Å, and lamellar distances were also expanded to 50 Å to avoid chains interdigitation; (iii) To introduce disorder, the whole systems were submitted to a high-temperature dynamics simulation (NPT, P = 1 atm, T = 500 K, 50 ps), providing a full relaxation along and between chains; (iv) According to the previous simulation for crystalline phase, two 200 ps-long consecutive molecular dynamics calculation (NPT, P = 1 atm, T = 298 K, snapshots saved every 1 ps) have been accomplished for thermalization and data analysis, respectively.

The width of the density of states (DOS) tail is an important parameter to reveal the effect of conformational disorder on charge transport. To generate the DOS, we extracted isolated polymer chains made of dodecamers among the 201 snapshots during the last molecular dynamics run to calculate their single energies at the DFT level (B3LYP/6-311g(d, p)). With a Gaussian broadening of 0.02 eV, we summed up the intensities of different broadened Gaussian functions at well-defined energy E and eventually got the DOS for contrastive molecular systems. Since electrons are the main carrier charges in n-type semiconductors, the distribution of LUMO, i.e., unoccupied DOS, is a key point. Then we fitted an exponential function to unoccupied DOS distributions with the equation[3]:

$$DOS_{unocc}(E) = \exp(\frac{E + c}{E_b})$$

where E_b is the characteristic depth of the trap states.

Section 15. Visualization of weak interaction and calculation of binding energy

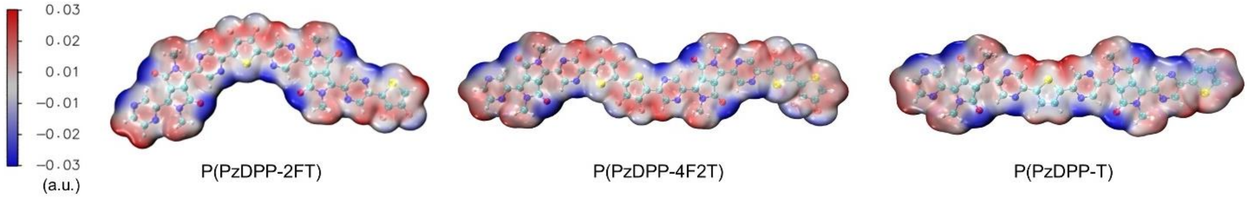

Supplementary Figure 43. Electrostatic surface potential of P(PzDPP-2FT), P(PzDPP-4F2T), and P(PzDPP-T).

Supplementary Fig. 43 shows the electrostatic surface potential (ESP) of the oligomers, which were generated by Multiwfn[10] and VMD[12]. After optimizing several starting molecular packing

structures of the anionic oligomers and $TDAE^+$ complexes, we found that cationic $TDAE^+$ can be easily bound in the zigzag cavity of P(PzDPP-2FT). For P(PzDPP-4F2T) and P(PzDPP-T), $TDAE^+$ tends to stack on the oligomers π-π stacking directions or far away from the backbone. To visualize interactions between polymer and dopant, Independent Gradient Model (IGM) analysis based on promolecular density is performed using the Multiwfn software[10]. IGM is a very useful tool for visually studying inter-fragment without the interference of intramolecular interactions[19]. Here, we summarize IGM approach briefly, considering two given fragments A and B. For more details, we refer the reader to IGM fundamentals in the original papers. For intermolecular interaction, IGM defines the δ_g^{inter} descriptor that uniquely defines intermolecular interaction regions:

$$\delta g^{inter} = \left|\nabla \rho^{IGM,inter}\right| - |\nabla \rho|$$

where $\nabla\rho$ stands for the electron density gradient and is an upper limit to $\nabla\rho^{IGM,inter}$. Non-zero values of δ_g^{inter} exclusively correspond to interaction situations: the larger δ_g^{inter}, the stronger the interaction. The 2D plot of δ_g^{inter} as a function of sign(λ_2)ρ (the sign of the second largest eigenvalue of electron density Hessian matrix and electron density) are performed with color scaling such that −0.5 sign(λ_2)ρ is colored blue representing an attractive interaction, and 0.5 sign(λ_2)ρ is colored red representing a steric repulsive interaction. IGM analysis in this work utilizes the B3LYP/6-31+G(d,p) electron density. All the isosurfaces are colored according to the BGR scheme over the range $-0.05 < \text{sign}(\lambda_2)\rho < 0.05$ *a.u.* 3-D isosurfaces (isovalues set equal to 0.003 *a.u.*) are generated with VMD. In addition to the isosurface plots, δ_g^{inter} vs. sign(λ_2)ρ plots are included for a full description of the electron density topology.

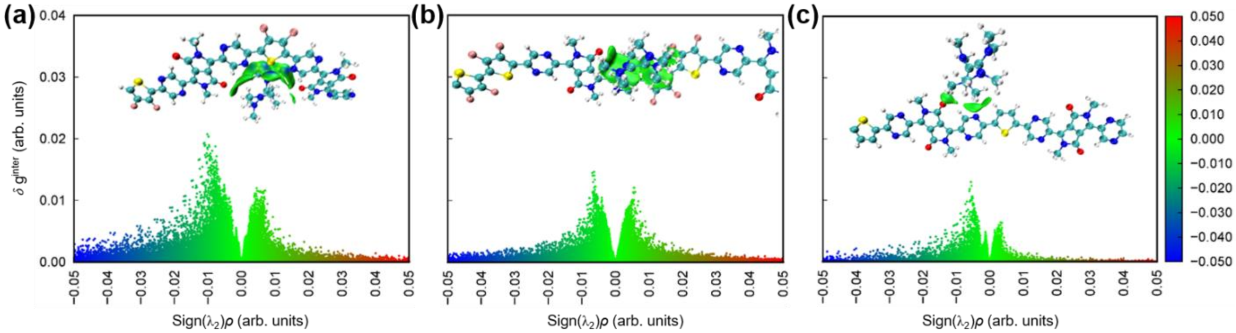

Supplementary Figure 44. δg^{inter}-isosurface and δg^{inter}-sign(λ_2)ρ 2D fingerprint-plots associated with the intermolecular interaction between (a) $P(PzDPP\text{-}2FT)^-$-$TDAE^+$, (b) $P(PzDPP\text{-}4F2T)^-$-$TDAE^+$ and (c) $P(PzDPP\text{-}T)^-$-$TDAE^+$.

Large continuous isosurface extended across the region of interaction between backbones and ionized dopant $TDAE^+$, indicating the existence of non-covalent interaction. The isosurface between $P(PzDPP\text{-}2FT)^-$ and $TDAE^+$ showed blue and green colors, suggesting the main interaction was due to hydrogen bonding and Van der Waals forces. However, only the weaker Van der Waals dispersion forces can be observed from the interaction between $P(PzDPP\text{-}4F2T)^-$ (or $P(PzDPP\text{-}T)^-$) and $TDAE^+$. Larger δ_g^{inter} in 2D fingerprint plot of $P(PzDPP\text{-}2FT)^-$-$TDAE^+$ proved the stronger intermolecular interaction with the biggest binding energy of −71.44 kcal/mol, compared to the other two collinear polymers (Supplementary Fig. 44 and Supplementary Table 3). All these results indicate that this zigzag backbone curvature can effectively capture TDAE dopants without much destruction to polymer packing and lead to enhanced miscibility and higher doping efficiency in P(PzDPP-2FT) film.

Followed by counterpoise correction with the aid of ORCA[20], single-point energy of ion-pairs and isolated polymer or dopant in the existence of each other were calculated, and binding energy is the energy difference between the complex and summary of the polymer and dopant, i.e., $\Delta G = E_{total} - E_{polymer} - E_{dopant}$.

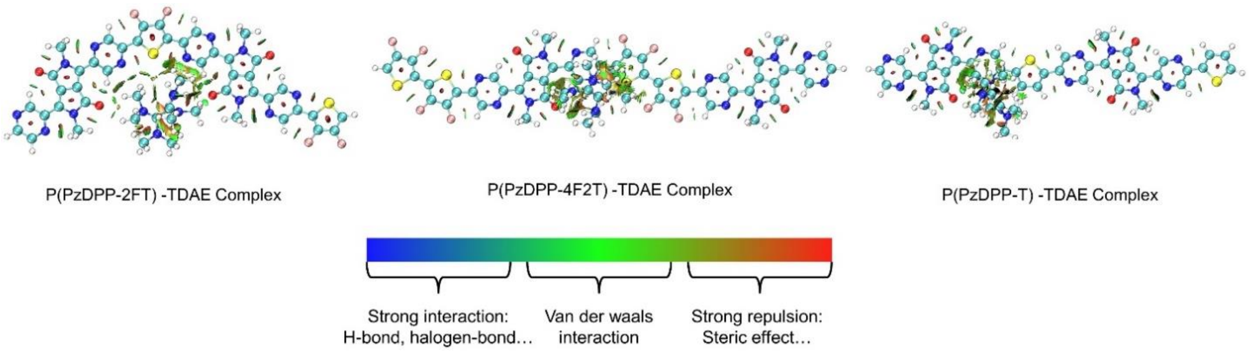

Supplementary Figure 45. Visualization of weak interactions between polymers and cationic $TDAE^+$.

Supplementary Table 3. Binding energy calculations for the three oligomers and $TDAE^+$ complex.

Polymer	P(PzDPP-2FT)	P(PzDPP-4F2T)	P(PzDPP-T)
E_{dopant} (kcal/mol)	−385451.93	−385452.22	−385459.00
$E_{polymer}$ (kcal/mol)	−2316734.00	−3258524.73	−2067537.00
E_{total} (kcal/mol)	−2702260.60	−3644043.49	−2453058.00
Δ G (kcal/mol)	−71.44	−66.54	−62.76

Section 16. Time-dependent decay of the electrical conductivity

The stability of the electrical conductivity of the doped P(PzDPP-2FT) films was measured, and the results showed that the *N*-DMBI and $CoCp_2$ doped P(PzDPP-2FT) films exhibited good stability. The TDAE doped P(PzDPP-2FT) films were unstable due to the volatilization of the dopant.

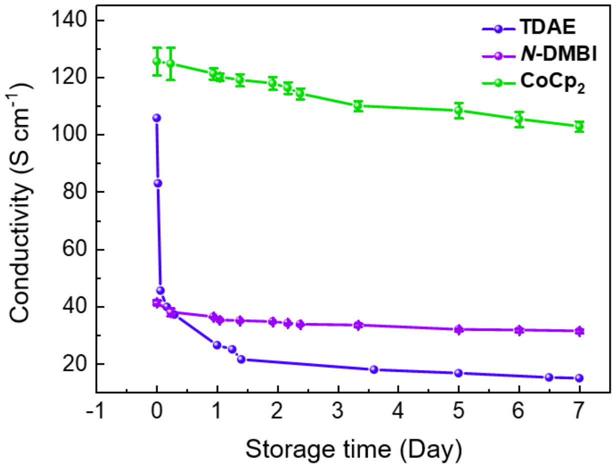

Supplementary Figure 46. Time-dependent decay of the electrical conductivity of three dopants doped P(PzDPP-2FT) films. The error bars have been determined as 2% of the experimental value.

The $CoCp_2$ doped P(PzDPP-2FT) thick films showed obviously better stability than its thin films due to the self-encapsulation effects[21,22].

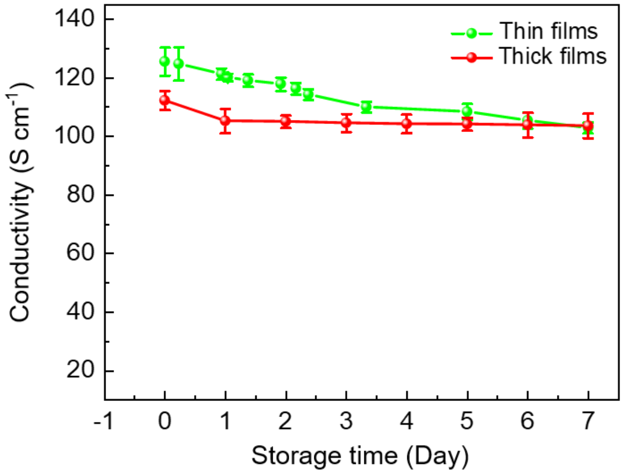

Supplementary Figure 47. Conductivity stability of the $CoCp_2$ doped P(PzDPP-2FT) thick films (> 1 μm) in comparison with the thin films (20 nm). The thin films were encapsulated with a

CYTOP layer. The conductivities were measured in air. The error bars have been determined as 2% of the experimental value.

Supplementary Table 4. Compiled literature values of the electrical conductivity maxima (σ_{max}) of solution-processable n-doped conjugated polymers and their LUMO energy levels.

Polymer	σ_{max} [S cm^{-1}]	LUMO [eV]	Reference
**P(PzDPP-2FT)**	129	-3.90[a]	This work
**P(PzDPP-4F2T)**	7.06	-3.82[a]	This work
**P(PzDPP-T)**	0.01	-3.76[a]	This work
**P(PzDPP-CT2)**	8.4	-4.03[a]	[23]
**FBDPPV**	14	-4.17[a]	[24]
**BBL**	2.4	-4.00	[25]
**P(PDI2OD-A)**	0.45	-4.19[a]	[26]
**FBDPPV**	12	--	[27]
**ClBDPPV**	0.62	-4.20[b]	[28]
**PNDTI-BBT-DT**	0.18	-4.40[a]	[29]
**PNDTI-BBT-DP**	5.0	-4.40[a]	[29]
**P(BTP-DPP)**	0.45	-3.69[b]	[30]
**P(gNDI-gT2)**	0.29	--	[31]
**TEG-N2200**	0.17	-3.69[a]	[32]
**PNDI2TEG-2Tz**	1.8	-4.26[a]	[33]
**P(NDI2OD-Tz2)**	0.1	-4.10[a]	[34]
**PDPF**	1.3	-4.11[b]	[35]
**2S-trans-PNDIT2**	0.006	-3.96[a]	[36]
**LPPV**	1.1	-4.49[a]	[21]
**PDTzTI**	4.6	-3.80[a]	[37]

[a)] Calculated from the cyclic voltammetry (CV) measurement; [b)] Calculated from the optical bandgaps and UPS measurement.

Section 17. Synthesis of P(PzDPP-2FT), P(PzDPP-4F2T), and P(PzDPP-T)

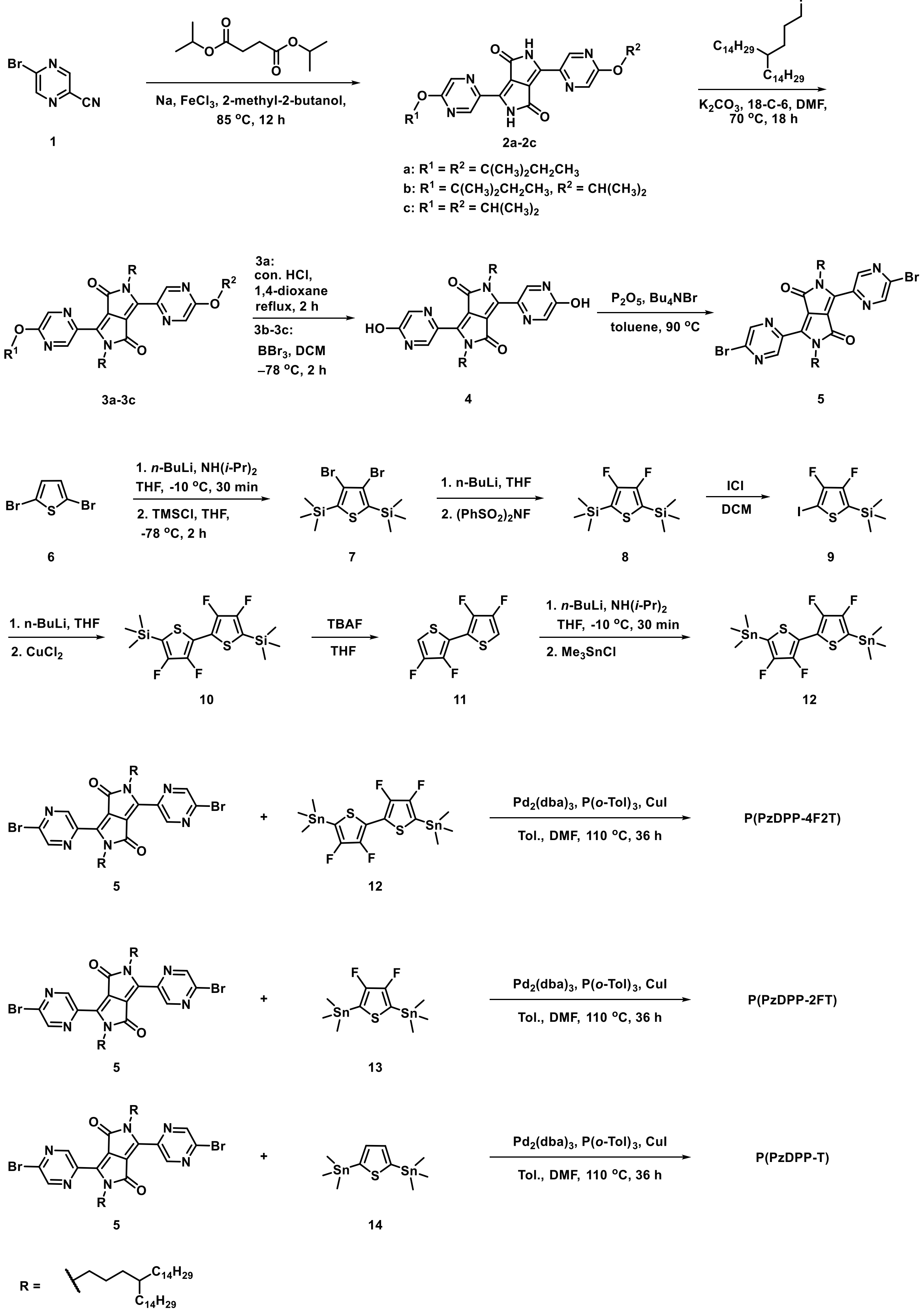

All chemical reagents were purchased and used as received unless otherwise indicated. All air and water-sensitive reactions were performed under a nitrogen atmosphere. Dichloromethane, tetrahydrofuran, toluene, and *N*,*N*-Dimethylformamide were dried by a JC Meyer solvent drying system prior to use. *N*-DMBI was purchased from Sigma-Aldrich and recrystallized from methanol prior to use. Compounds **13** and **14** were purchased from Derthon Optoelectronic Materials Science Technology Co LTD and recrystallized from ethanol prior to use. Compounds **1-4** were synthesized following the procedures from the previous literature[23].

^{1}H NMR and ^{13}C NMR spectra were recorded on Bruker ARX-400 (400 MHz). All chemical shifts were reported in parts per million (ppm). ^{1}H NMR chemical shifts were referenced to $CDCl_3$ (7.26 ppm), and ^{13}C NMR chemical shifts were referenced to $CDCl_3$ (77.16 ppm). Mass spectra were recorded on an AB Sciex-5800 MALDI-TOF mass spectrometer and a Bruker Solarix XR mass spectrometer. Elemental analyses were performed on Vario EL elemental analyzer.

Synthesis of 5

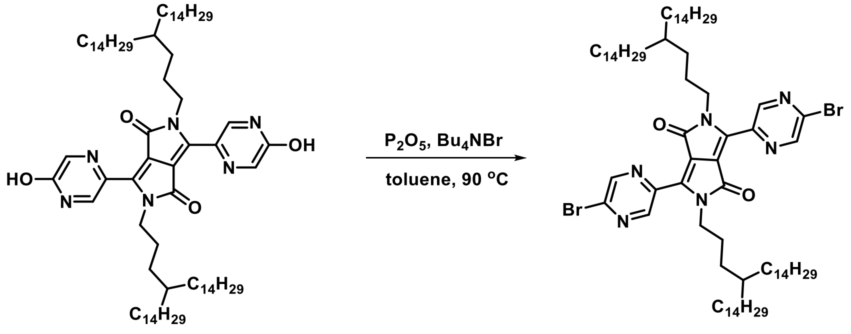

4 5

Under nitrogen atmosphere, a 100 mL Schlenk tube was charged with a suspension of compound **4** (1.28 g, 1.05 mmol), P_2O_5 (1.28 g, 9.03 mmol) and tetrabutylammonium bromide (1.49 g, 4.61 mmol) in 30 mL dry toluene. The resulting mixture was heated to 90 °C and stirred for 4.5 h. Then the reaction mixture was cooled to room temperature and concentrated under reduced pressure. The residue was purified by silica gel chromatography (dichloromethane/petrol ether = 1:5 ~ 3:1) to afford compound **5** (1.05 g, yield 74%) as deep purple solid. ^{1}H NMR ($CDCl_3$, 400 MHz, ppm) δ 10.01 (d, J = 1.2 Hz, 2H), 8.75 (d, J = 1.6 Hz, 2H), 4.18 (t, J = 7.6 Hz, 4H), 1.52-1.62 (m, 4H), 1.13-1.33 (m, 110H), 0.87 (t, J = 6.6 Hz, 12H); ^{13}C NMR ($CDCl_3$, 100 MHz, ppm) δ 161.76, 148.51, 146.41, 142.26, 141.89, 112.24, 43.27, 37.10, 33.64, 32.09, 30.54, 30.28,

29.92, 29.88, 29.83, 29.53, 27.22, 26.81, 22.86, 14.29; MALDI-TOF HRMS calcd. for $(M + H)^+$: 1345.8935, Found: 1345.9020.

Synthesis of 7

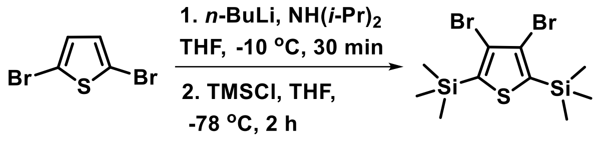

6 **7**

Under nitrogen atmosphere, a 250 mL two-necked round bottom flask was charged with 40 mL dry THF. After the system was cooled to −10 °C, *n*-BuLi (36.7 mL, 2.4 M, 88.08 mmol) was added in one portion. Then diisopropylamine (8.77 g, 86.66 mmol) was added dropwise. The resulting mixture was stirred at −10 °C for 0.5 h. Under nitrogen atmosphere, a 500 mL two-necked round bottom flask was charged with a solution of compound **6** (10 g, 41.33 mmol) in 100 mL dry THF. After the solution was cooled to −78 °C, the new prepared LDA solution was added dropwise. The resulting mixture was stirred at −78 °C for 1 h. Then trimethylchlorosilane (11 mL, 86.77 mmol) was added dropwise. After the addition was completed, the mixture was allowed to warm to room temperature and stirred overnight. Water was added to quench the reaction, and the mixture was extracted with dichloromethane (200 mL × 4). The combined organic layers were dried over anhydrous Na_2SO_4 and concentrated under reduced pressure. The residue was distilled under reduced pressure to afford compound **7** (14.3 g, yield 90%) as a colorless oil. ^1H NMR ($CDCl_3$, 400 MHz, ppm) δ 0.40 (s, 18H).

Synthesis of 8

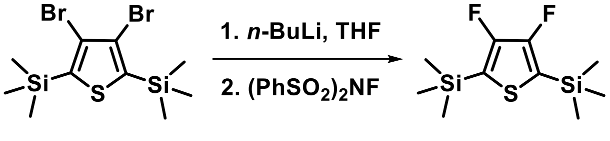

7 **8**

Under nitrogen atmosphere, a 100 mL three-necked round bottom flask was charged with a solution of compound **7** (5 g, 12.94 mmol) in 35 mL dry THF. After the solution was cooled to −78 °C, *n*-BuLi (5.5 mL, 2.4 M in hexane) was added dropwise. The resulting mixture was stirred at −78 °C for 0.5 h, and $(PhSO_2)_2NF$ (4.25 g, 13.50 mmol) was added. The reaction mixture was stirred at −78 °C for 0.5 h. Then *n*-BuLi (2.8 mL, 2.4 M in hexane) was added dropwise. The

resulting mixture was stirred at −78 °C for 0.5 h, and $(PhSO_2)_2NF$ (2.15 g, 6.80 mmol) was added. The reaction mixture was stirred at −78 °C for 0.5 h. Then *n*-BuLi (1.4 mL, 2.4 M in hexane) was added dropwise. The resulting mixture was stirred at −78 °C for 0.5 h, and $(PhSO_2)_2NF$ (1.08 g, 3.40 mmol) was added. The reaction mixture was stirred at −78 °C for 0.5 h. Then *n*-BuLi (1.4 mL, 2.4 M in hexane) was added dropwise. The resulting mixture was stirred at −78 °C for 0.5 h, and $(PhSO_2)_2NF$ (1.08 g, 3.40 mmol) was added. After the addition was completed, the reaction mixture was allowed to warm to room temperature and stirred overnight. Then water was added to quench the reaction, and the mixture was extracted with ethyl acetate (100 mL × 4). The combined organic layers were dried over anhydrous Na_2SO_4, concentrated, and the residue was purified by silica gel chromatography (petrol ether) to afford compound **8** (1.86 g, yield: 54%) as a colorless oil. ^1H NMR ($CDCl_3$, 400 MHz, ppm) δ 0.34 (s, 18H); ^{13}C NMR ($CDCl_3$, 100 MHz, ppm) δ 151.22 (dd, J = 259.7, 23.4 Hz), 120.87 (dd, J = 17.2, 7.5 Hz), −0.78 (s).

Synthesis of 9

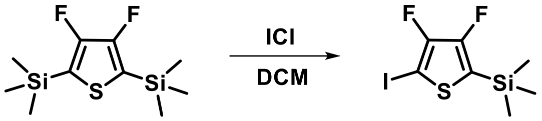

8 9

Under nitrogen atmosphere, 35 mL dry dichloromethane and compound **8** (1.86 g, 7.03 mmol) were added to a 100 mL two-necked round bottom flask. Then a solution of ICl (1.73 g, 10.66 mmol) in 10 mL dry dichloromethane was added dropwise. After the addition was completed, the reaction mixture was stirred at 25 °C overnight. The reaction mixture was quenched with saturated aqueous $NaHCO_3$. The organic layer was separated and washed with saturated aqueous $NaHCO_3$ (20 mL × 2). Then the organic layer was dried over anhydrous Na_2SO_4, concentrated under reduced pressure, and the residue was purified by recycling SEC to afford compound **9** (1.05 g, 47%) as a colorless oil. ^1H NMR ($CDCl_3$, 400 MHz, ppm) δ 0.33 (s, 9H); ^{19}F NMR ($CDCl_3$, 400 MHz, ppm) δ −126.18 (d, J = 12.5 Hz), −128.24 (d, J = 15.5 Hz); ^{13}C NMR ($CDCl_3$, 100 MHz, ppm) δ 150.47 (dd, J = 61.2, 23.6 Hz), 147.86 (dd, J = 57.4, 23.7 Hz), 122.21 (d, J = 26.5 Hz), 57.68 (d, J = 23.2 Hz), −0.78 (s).

Synthesis of 10

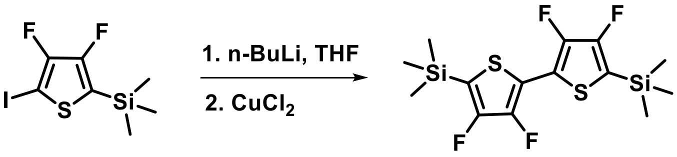

9 10

Under nitrogen atmosphere, compound **9** (1.05 g, 3.30 mmol) and 25 mL dry THF were added to a 100 mL two-necked round bottom flask. When the solution was cooled to −78 °C, *n*-BuLi (1.54 mL, 2.4 M in hexane) was added dropwise. After the addition was completed, the reaction mixture was stirred at −78 °C for 1 h. Anhydrous $CuCl_2$ (666 mg, 4.97 mmol) was added in one portion. The reaction mixture was stirred at −78 °C for 1 h. Then the mixture was allowed to warm to room temperature and stirred overnight. After the reaction was completed, the mixture was quenched with water. The resulting mixture was extracted with dichloromethane (100 mL × 3). The combined organic layers were dried over anhydrous Na_2SO_4, concentrated under reduced pressure, and the residue was purified by recycling SEC to afford compound **10** (406 mg, yield: 64%) as a light yellow solid. ^{1}H NMR ($CDCl_3$, 400 MHz, ppm) δ 0.36 (s, 18H); ^{19}F NMR ($CDCl_3$, 500 MHz, ppm) δ −128.54 (d, J = 14.5 Hz), −134.40 (d, J = 15.0 Hz); ^{13}C NMR ($CDCl_3$, 100 MHz, ppm) δ 149.3 (dd, J = 256.0, 18.0 Hz), 142.76 (dd, J = 269.0, 25.0 Hz), 115.50 (s), 114.74 (d, J = 21.0 Hz), −0.77 (s).

Synthesis of 11

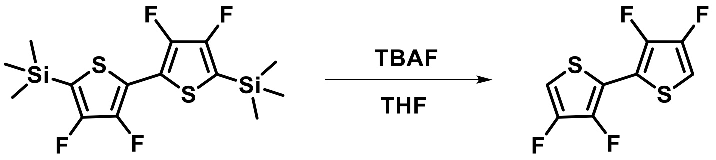

10 11

Under nitrogen atmosphere, a 50 mL two-necked round bottom flask was charged with a solution of compound **10** (406 mg, 1.06 mmol) in 6 mL dry THF. After the solution was cooled to 0 °C, TBAF (6.4 mL, 1.0 M in THF) was added dropwise. After the addition was completed, the reaction mixture was allowed to warm to room temperature and stirred for 12 h. Then the reaction was quenched with water, and the mixture was extracted with hexane (50 mL × 3). The combined organic layers were dried over anhydrous Na_2SO_4, concentrated under reduced pressure, and the residue was purified by silica gel chromatography (hexane) to afford compound **11** (216 mg, yield:

85%) as a white solid. ^{1}H NMR ($CDCl_3$, 400 MHz, ppm) δ 6.76 (d, J = 2.0 Hz, 2H); ^{13}C NMR ($CDCl_3$, 100 MHz, ppm) δ 145.55 (dd, J = 20, 261 Hz), 142.26 (dd, J = 20, 265 Hz), 110.80-1101.05 (m), 102.66 (dt, J = 4, 15 Hz).

Synthesis of 12

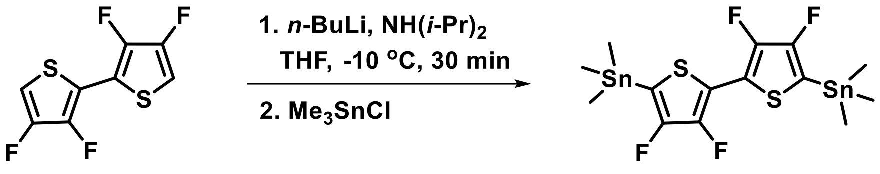

11 **12**

Under nitrogen atmosphere, a 50 mL two-necked round bottom flask was charged with 3 mL dry THF. After the system was cooled to −78 °C, *n*-BuLi (1.5 mL, 2.2 M in hexane) was added in one portion. Then diisopropylamine (335 mg, 3.3 mmol) was added dropwise. The resulting mixture was stirred at −78 °C for 0.5 h. Under nitrogen atmosphere, another 100 mL three-necked round bottom flask was charged with a solution of compound **11** (216 mg, 0.91 mmol) in 10 mL dry THF. After the solution was cooled to −78 °C, the new prepared LDA solution was added dropwise. The resulting mixture was stirred at −78 °C for 45 min, then Me_3SnCl (669 mg, 3.3 mmol) was added in one portion. After the addition was completed, the reaction mixture was allowed to warm to room temperature and stirred for 2 h. The reaction mixture was quenched with water, and the mixture was extracted with hexane (50 mL × 3). The combined organic layers were dried over anhydrous Na_2SO_4, concentrated under reduced pressure, and the residue was purified by recycling SEC to afford compound **12** (360 mg, yield: 71%) as a white solid. ^{1}H NMR ($CDCl_3$, 400 MHz, ppm) δ 0.44 (s, 18H); ^{19}F NMR ($CDCl_3$, 500 MHz, ppm) δ −128.74 (*d*, J = 16 Hz), −136.16 (*dd*, J =2, 16 Hz).

Synthesis of P(PzDPP-4F2T)

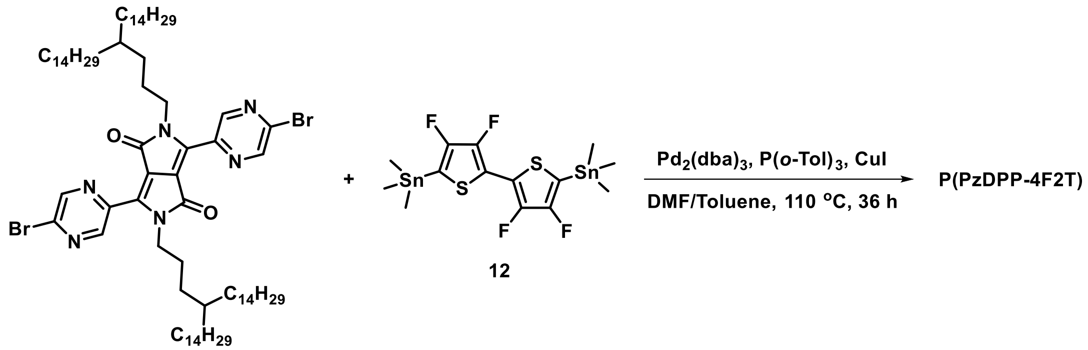

5 **12**

A 50 mL Schlenk tube was added complex **5** (50 mg, 0.037 mmol), complex **12** (20.10 mg, 0.037 mmol), $Pd_2(dba)_3$ (1.36 mg, 4 mol%), P(*o*-Tol)$_3$ (1.81 mg, 16 mol%), CuI (1.41 mg, 20 mol%), 1.6 mL anhydrous DMF and 8 mL anhydrous toluene. The tube was placed in liquid nitrogen to freeze the solution and then evacuation. This procedure was repeated three times. The sealed tube was heated to 110 ^{o}C and stirred for 36 h. After the reaction mixture was cooled to room temperature, *N,N'*-Diethylphenylazothioformamide (3 mg, 0.014 mmol) was added, and the resulting mixture was stirred at 80 ^{o}C for 30 min. The reaction mixture was poured into 200 mL methanol to precipitate the polymer, which was filtered, and the solid was placed in Soxhlet extractor and extracted successively with methanol, acetone, hexane, tetrahydrofuran, dichloromethane, chloroform, and chlorobenzene. The chlorobenzene solution was concentrated under reduced pressure, and the concentrated solution was poured into 200 mL methanol to precipitate the polymer. The suspension was filtered and dried in vacuum to afford **P(PzDPP-4F2T)** as a dark blue solid (32 mg, 62%). ^{1}H NMR ($CDCl_2CDCl_2$, 500 MHz, 363 K, ppm) δ 9.72, 9.36, 9.00, 4.20, 1.80-1.15, 0.83. Elemental Anal. Calcd. for $(C_{86}H_{136}F_4N_6O_2S_2)_n$: C, 72.43; H, 9.61; N, 5.89; Found: C, 71.67; H, 9.60; N, 5.55.

Synthesis of P(PzDPP-2FT)

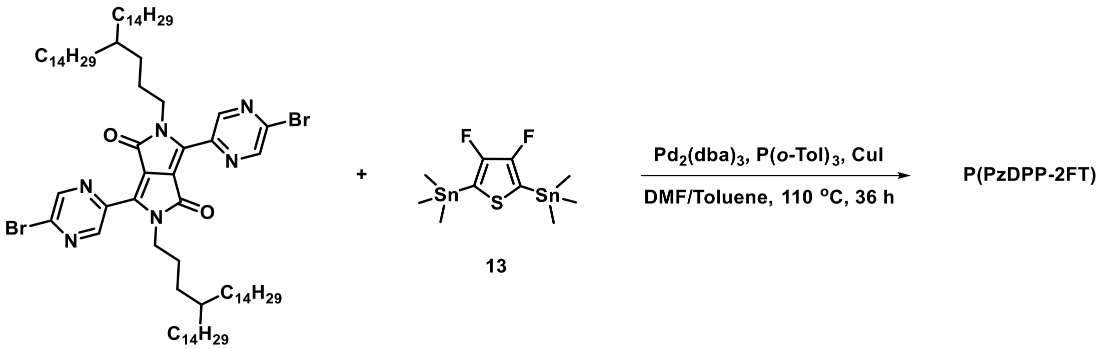

5 13

A 50 mL Schlenk tube was added complex **5** (50 mg, 0.037 mmol), complex **13** (19.98 mg, 0.037 mmol), $Pd_2(dba)_3$ (1.35 mg, 4 mol%), P(*o*-Tol)$_3$ (1.80 mg, 16 mol%), CuI (1.41 mg, 20 mol%), 1.6 mL anhydrous DMF and 8 mL anhydrous toluene. The tube was placed in liquid nitrogen to freeze the solution and then evacuation. This procedure was repeated three times. The sealed tube was heated to 110 ^{o}C and stirred for 36 h. After the reaction mixture was cooled to room temperature, *N,N'*-Diethylphenylazothioformamide (3 mg, 0.014 mmol) was added, and the resulting mixture was stirred at 80 ^{o}C for 30 min. Then the reaction mixture was poured into 200 mL methanol to precipitate the polymer, which was filtered, and the solid was placed in Soxhlet extractor and extracted successively with methanol, acetone, hexane, tetrahydrofuran, dichloromethane, chloroform, and chlorobenzene. The chlorobenzene solution was concentrated under reduced pressure, and the concentrated solution was poured into 200 mL methanol to precipitate the polymer. The suspension was filtered and dried in vacuum to afford **P(PzDPP-2FT)** as a dark blue solid (28 mg, 54%). ^{1}H NMR ($CDCl_2CDCl_2$, 500 MHz, 363 K, ppm) δ 10.09, 8.81, 4.21, 1.50-1.00, 0.80. Elemental Anal. Calcd. for $(C_{82}H_{136}F_2N_6O_2S)_n$: C, 75.29; H, 10.48; N, 6.42; Found: C, 74.42; H, 10.30; N, 6.15.

Synthesis of P(PzDPP-T)

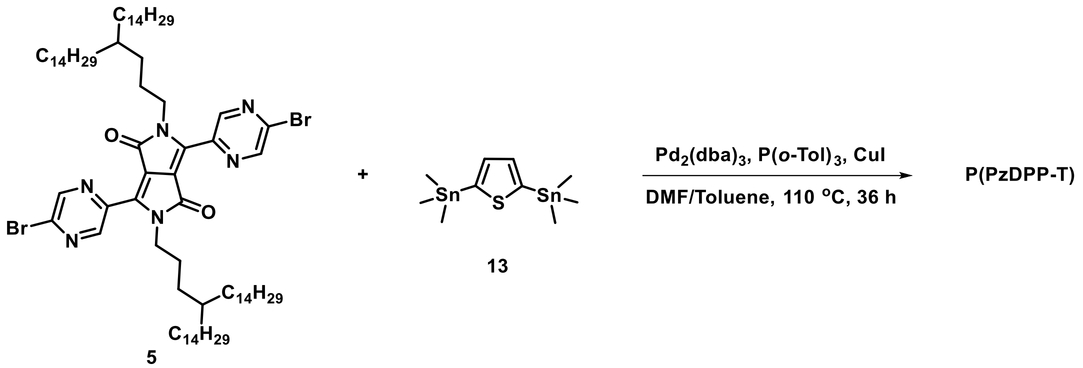

A 50 mL Schlenk tube was added complex **5** (50 mg, 0.037 mmol), complex **13** (15.20 mg, 0.037 mmol), $Pd_2(dba)_3$ (1.35 mg, 4 mol%), P(*o*-Tol)$_3$ (1.80 mg, 16 mol%), CuI (1.41 mg, 20 mol%), 1.6 mL anhydrous DMF and 8 mL anhydrous toluene. The tube was placed in liquid nitrogen to freeze the solution and then evacuation. This procedure was repeated three times. The sealed tube was heated to 110 ^{o}C and stirred for 36 h. After the reaction mixture was cooled to room temperature, *N,N'*-Diethylphenylazothioformamide (3 mg, 0.014 mmol) was added, and the resulting mixture was stirred at 80 ^{o}C for 30 min. Then the reaction mixture was poured into 200 mL methanol to precipitate the polymer, which was filtered, and the solid was placed in Soxhlet extractor and extracted successively with methanol, acetone, and hexane. The hexane solution was concentrated under reduced pressure, and the concentrated solution was poured into 200 mL methanol to precipitate the polymer. The suspension was filtered and dried in vacuum to afford **P(PzDPP-T)** as a dark blue solid (45 mg, 95%). ^{1}H NMR ($CDCl_2CDCl_2$, 500 MHz, 363 K, ppm) δ 9.93, 8.82, 7.64, 4.23, 1.80-1.00, 0.79. Elemental Anal. Calcd for $(C_{82}H_{138}N_6O_2S)$n: C, 77.42; H, 10.93; N, 6.61; Found: C, 74.46; H, 10.71; N, 6.09.

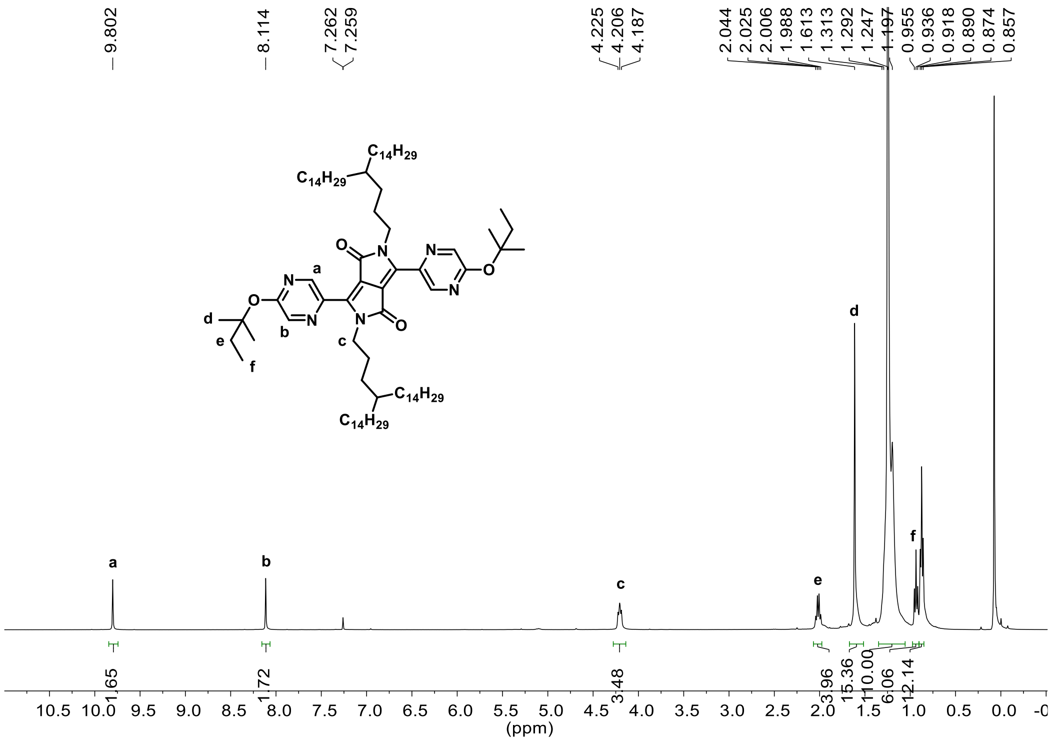

Supplementary Figure 48. ^{1}H NMR spectrum of compound **3a**

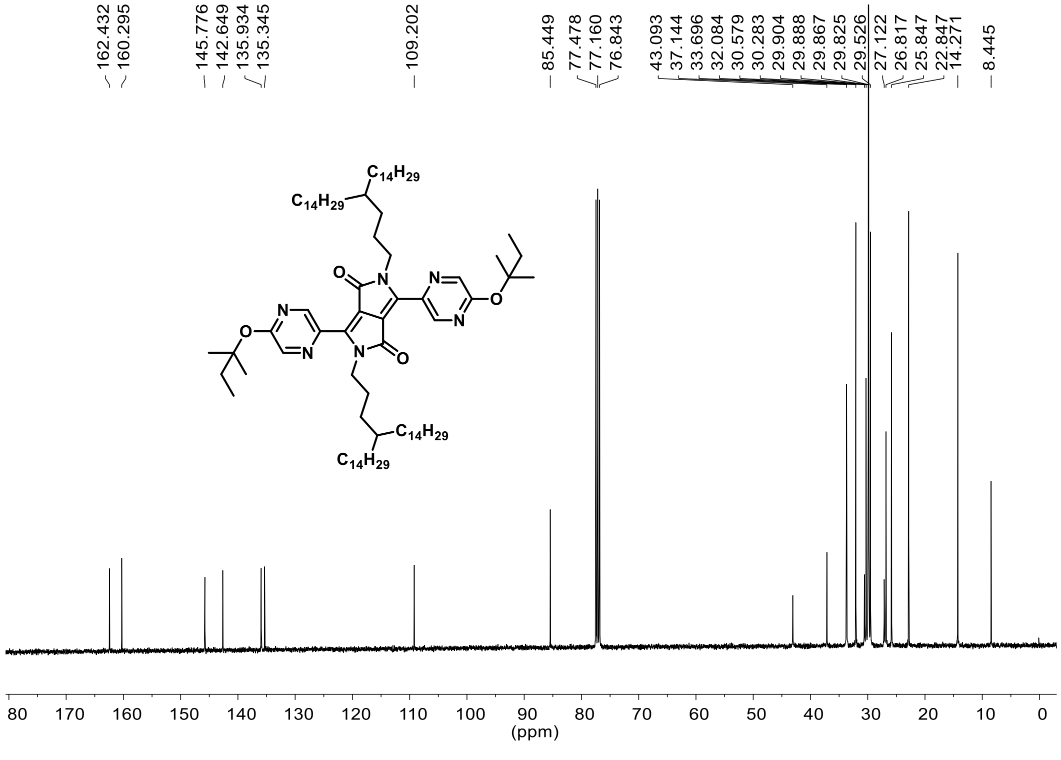

Supplementary Figure 49. ^{13}C NMR spectrum of compound **3a**

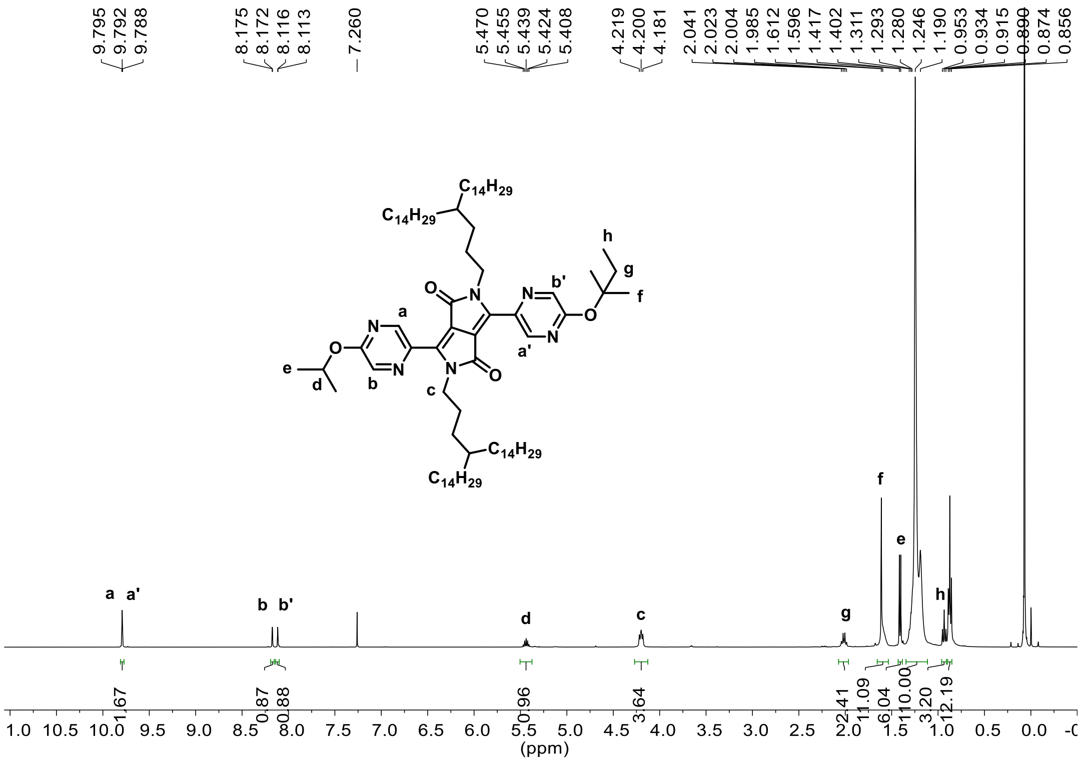

Supplementary Figure 50. ^{1}H NMR spectrum of compound **3b**

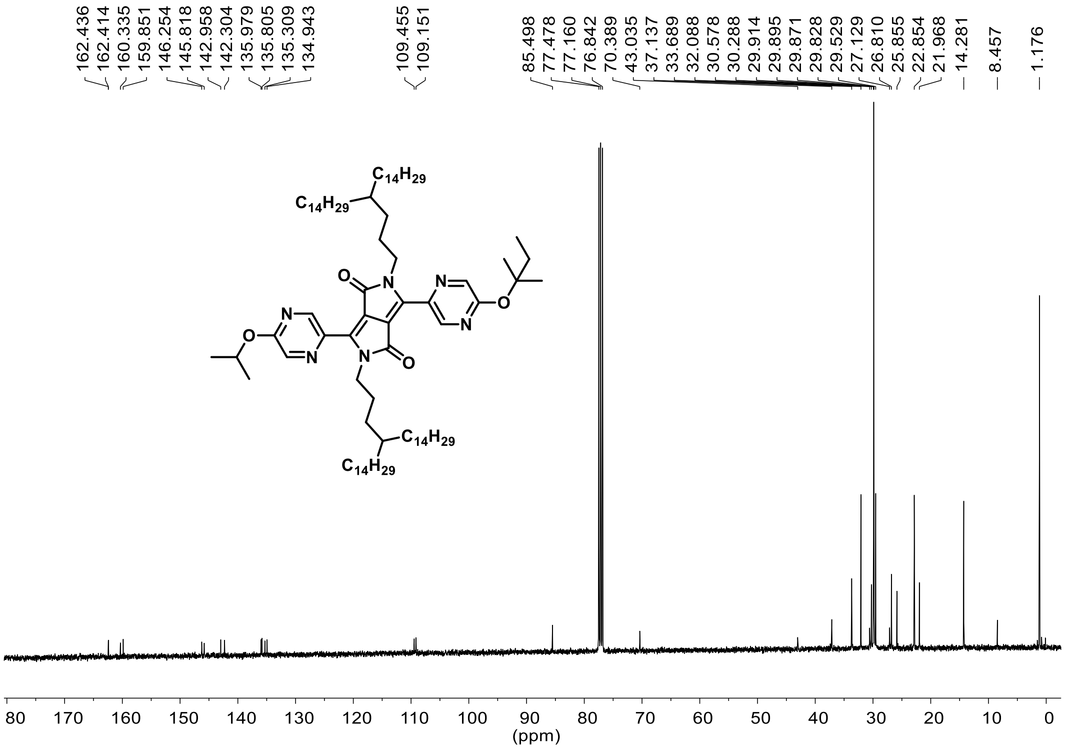

Supplementary Figure 51. ^{13}C NMR spectrum of compound **3b**

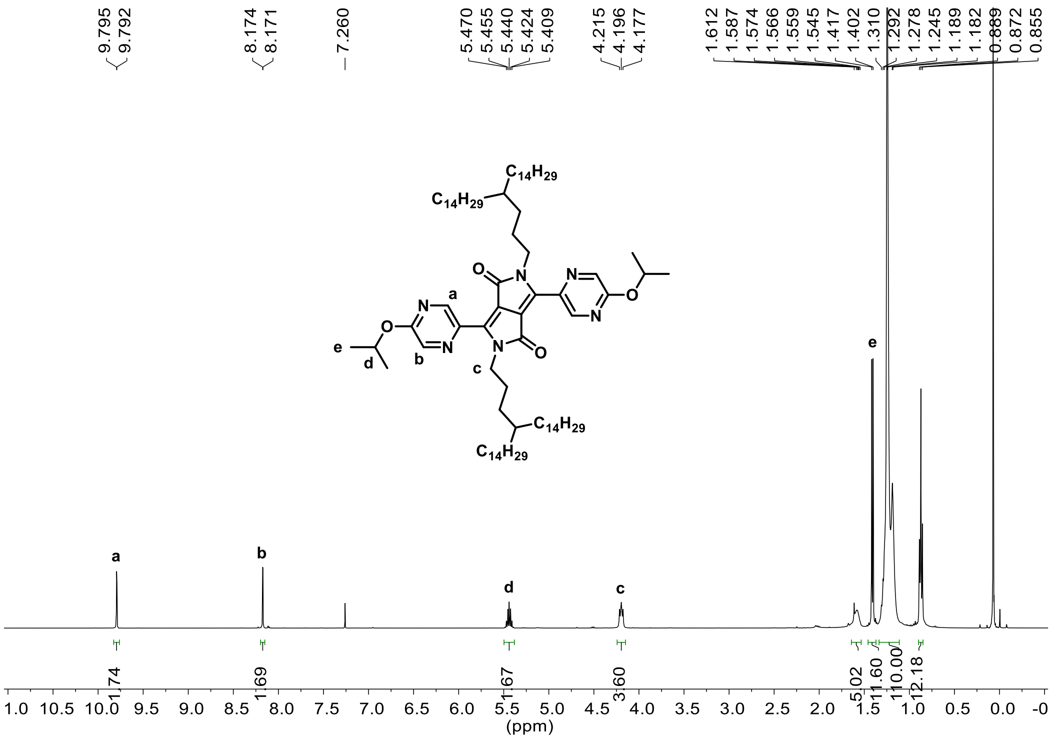

Supplementary Figure 52. ^{1}H NMR spectrum of compound **3c**

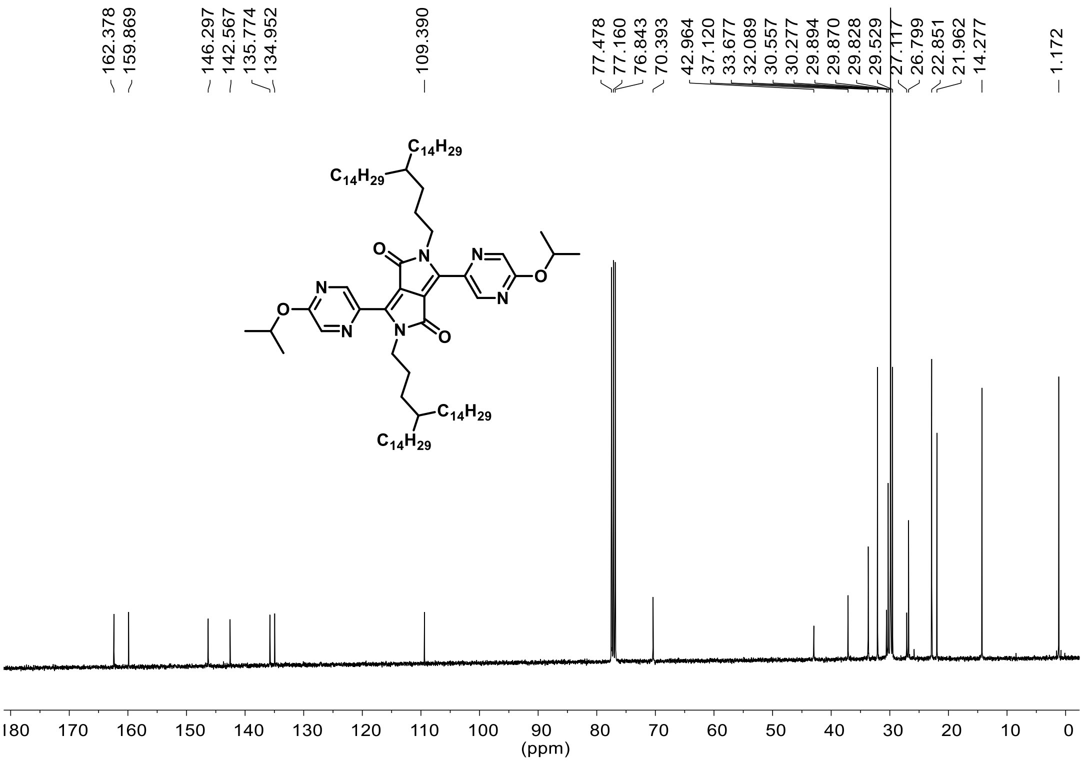

Supplementary Figure 53. ^{13}C NMR spectrum of compound **3c**

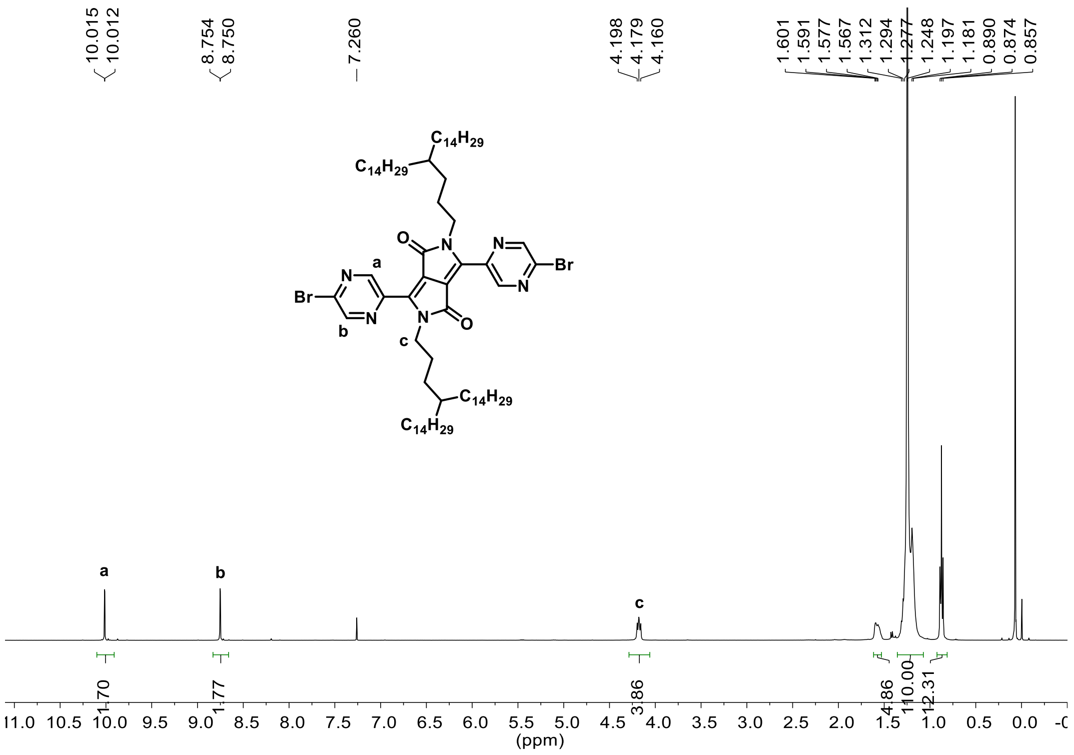

Supplementary Figure 54. ^{1}H NMR spectrum of compound **5**

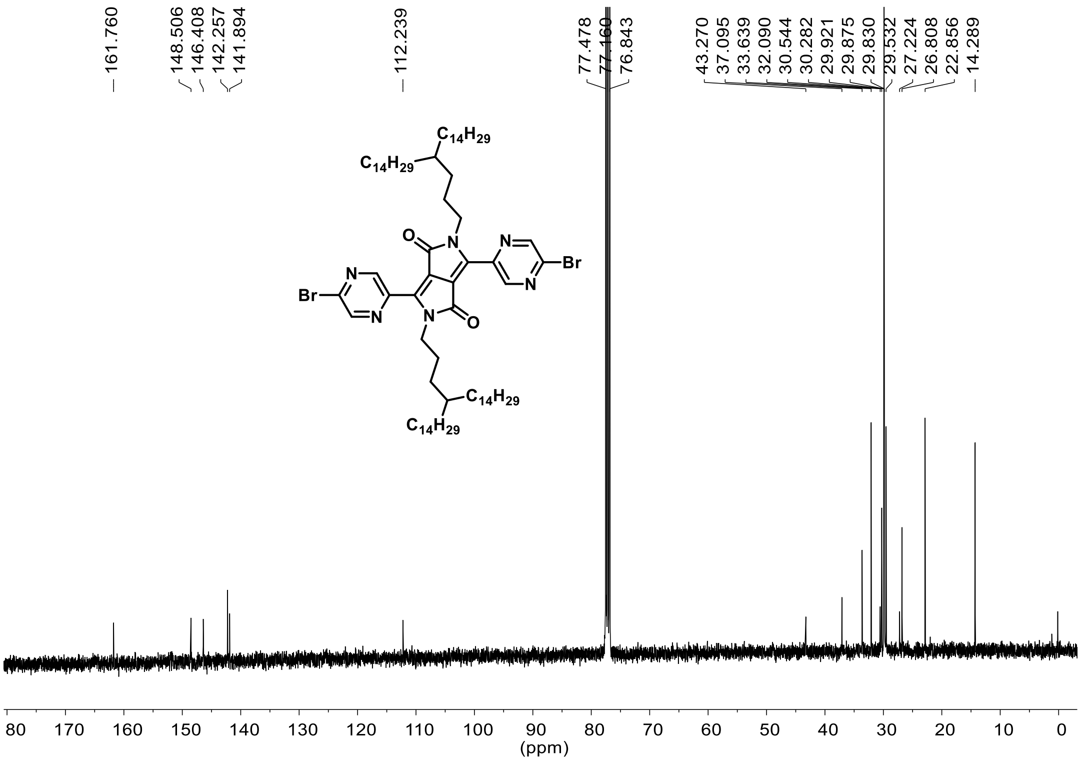

Supplementary Figure 55. ^{13}C NMR spectrum of compound **5**

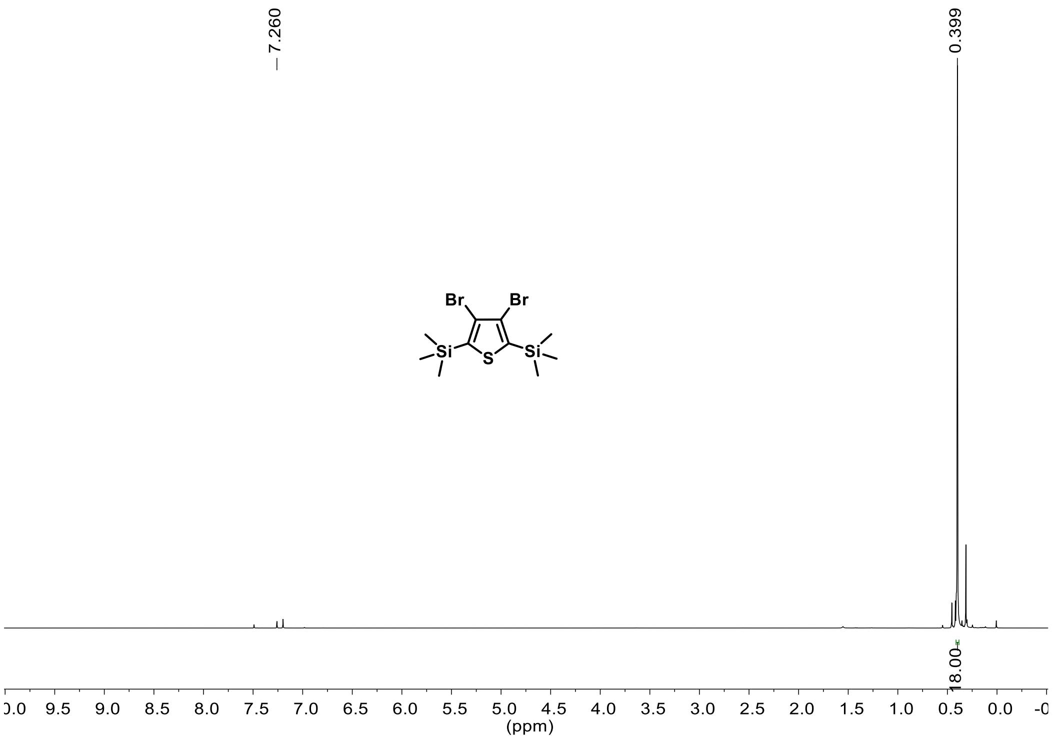

Supplementary Figure 56. ^{1}H NMR spectrum of compound **7**

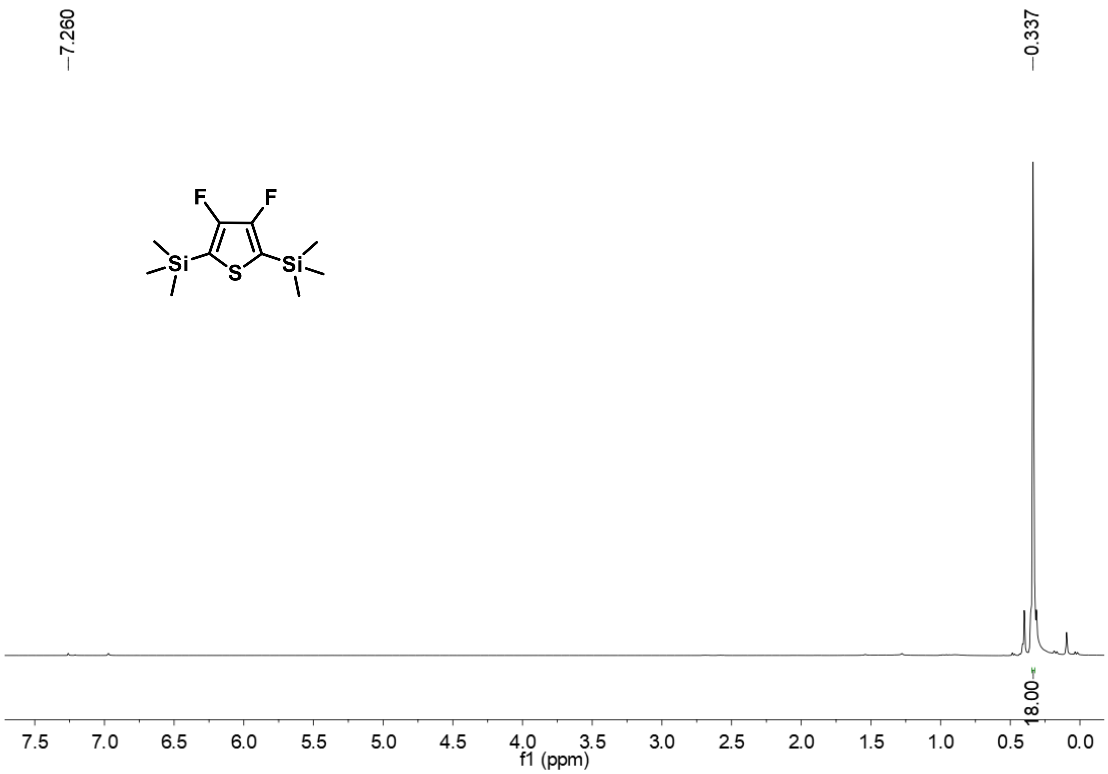

Supplementary Figure 57. ^{1}H NMR spectrum of compound **8**

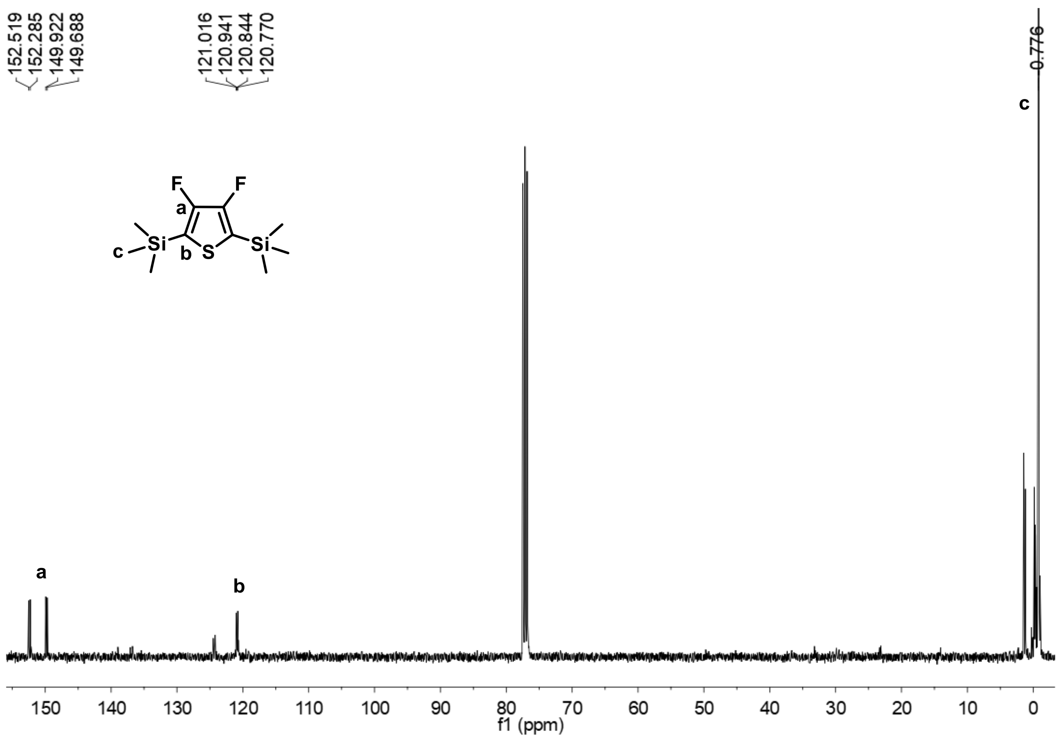

Supplementary Figure 58. ^{13}C NMR spectrum of compound **8**

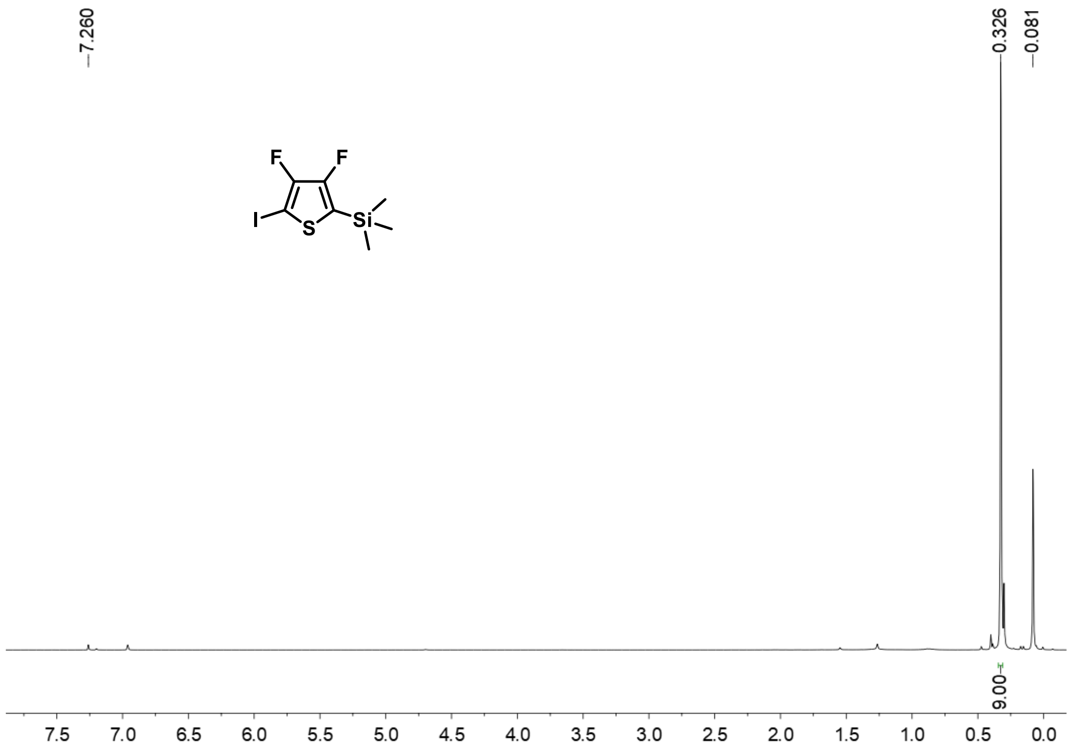

Supplementary Figure 59. ^{1}H NMR spectrum of compound **9**

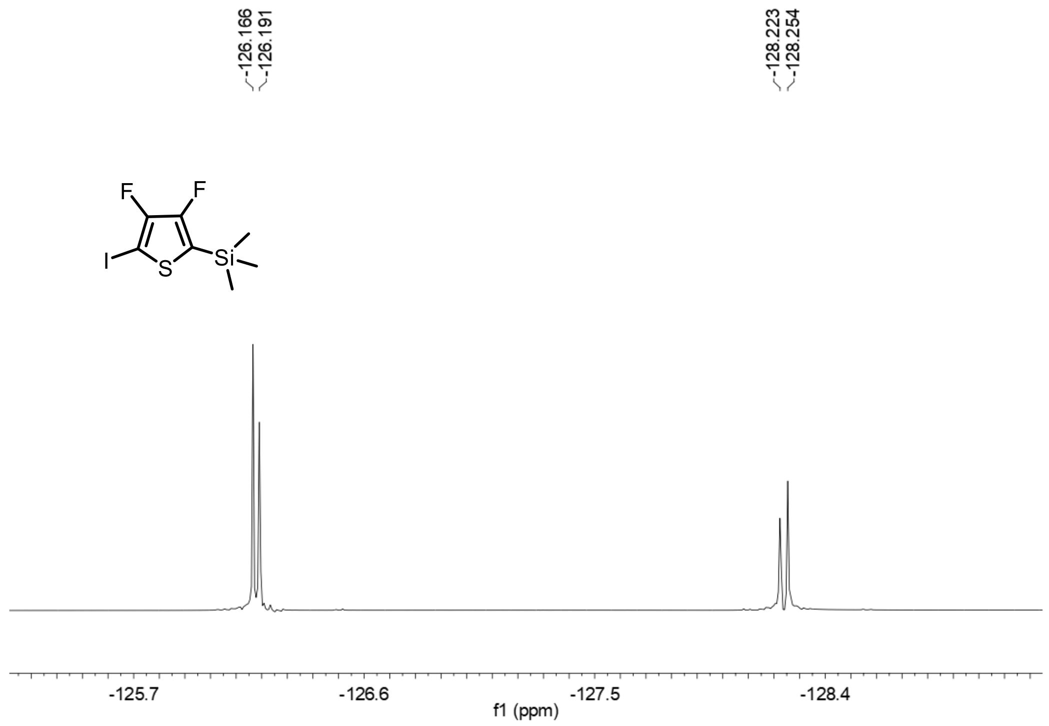

Supplementary Figure 60. ^{19}F NMR spectrum of compound **9**

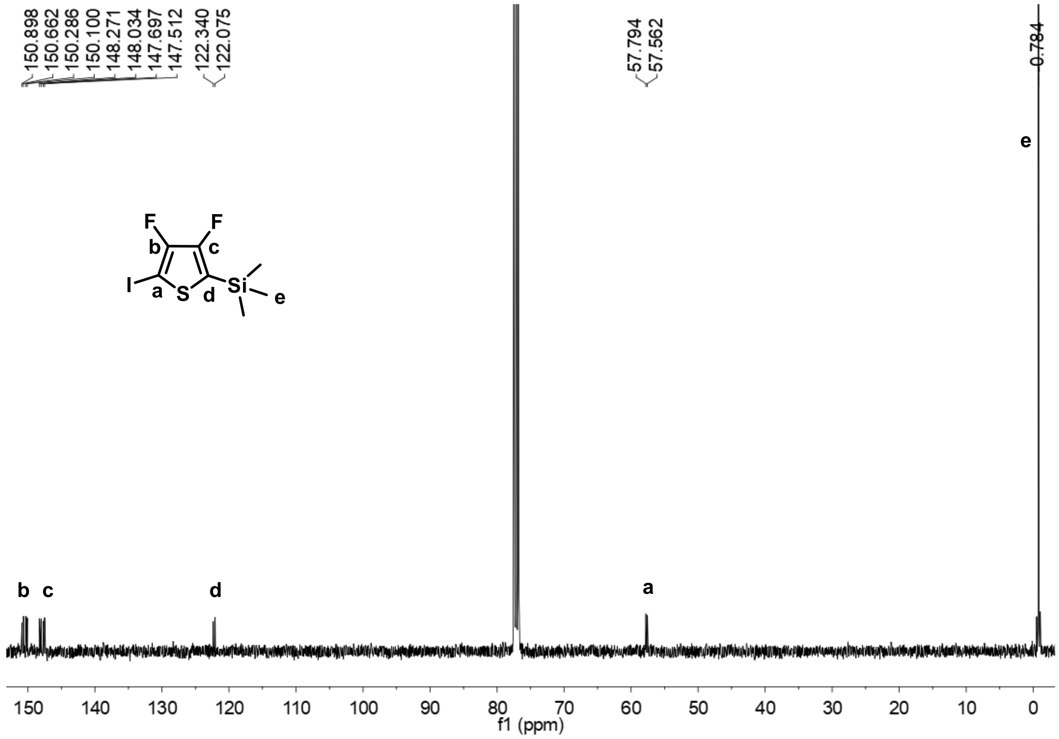

Supplementary Figure 61. ^{13}C NMR spectrum of compound **9**

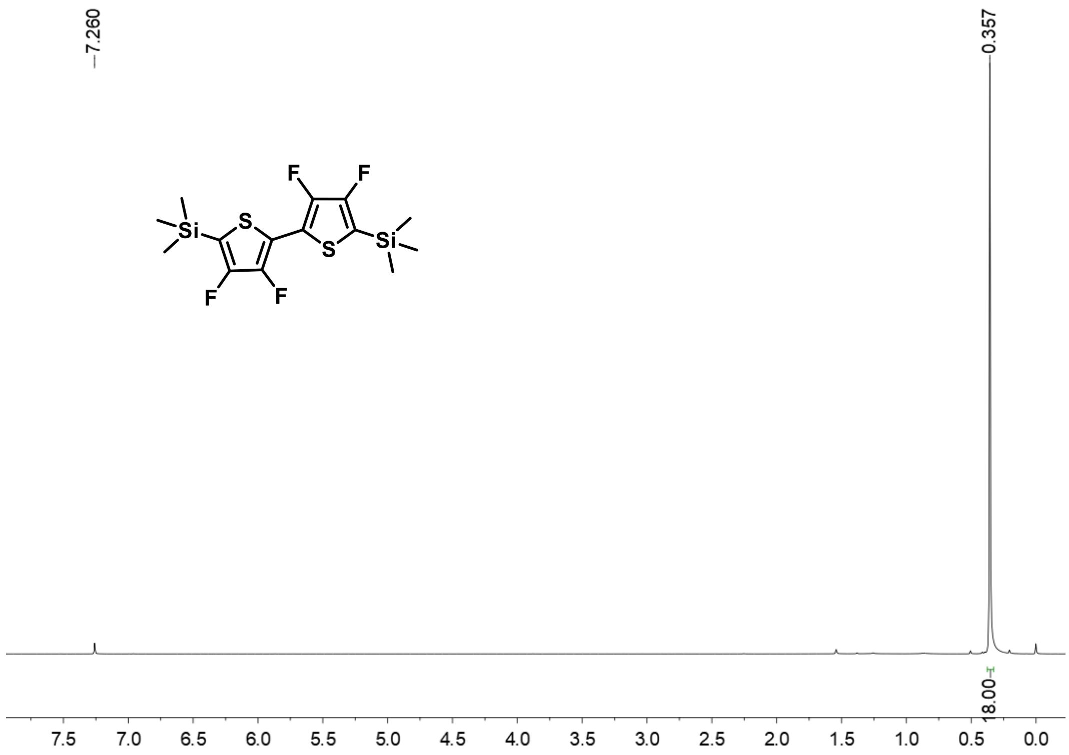

Supplementary Figure 62. ^{1}H NMR spectrum of compound **10**

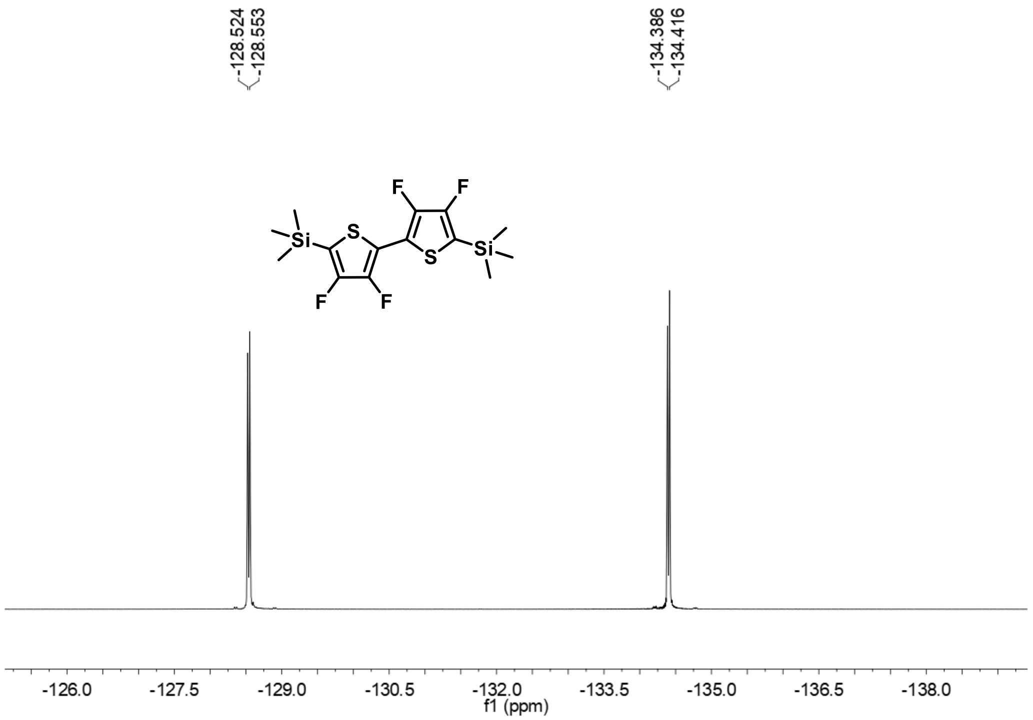

Supplementary Figure 63. ^{19}F NMR spectrum of compound **10**

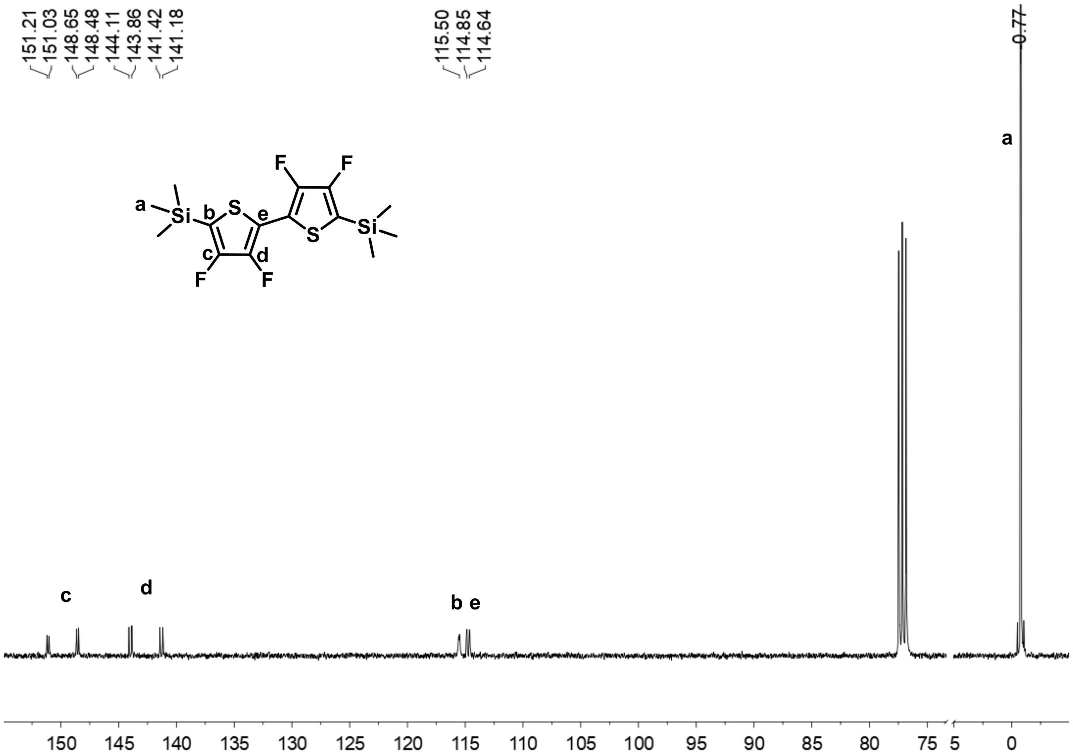

Supplementary Figure 64. ^{13}C NMR spectrum of compound **10**.

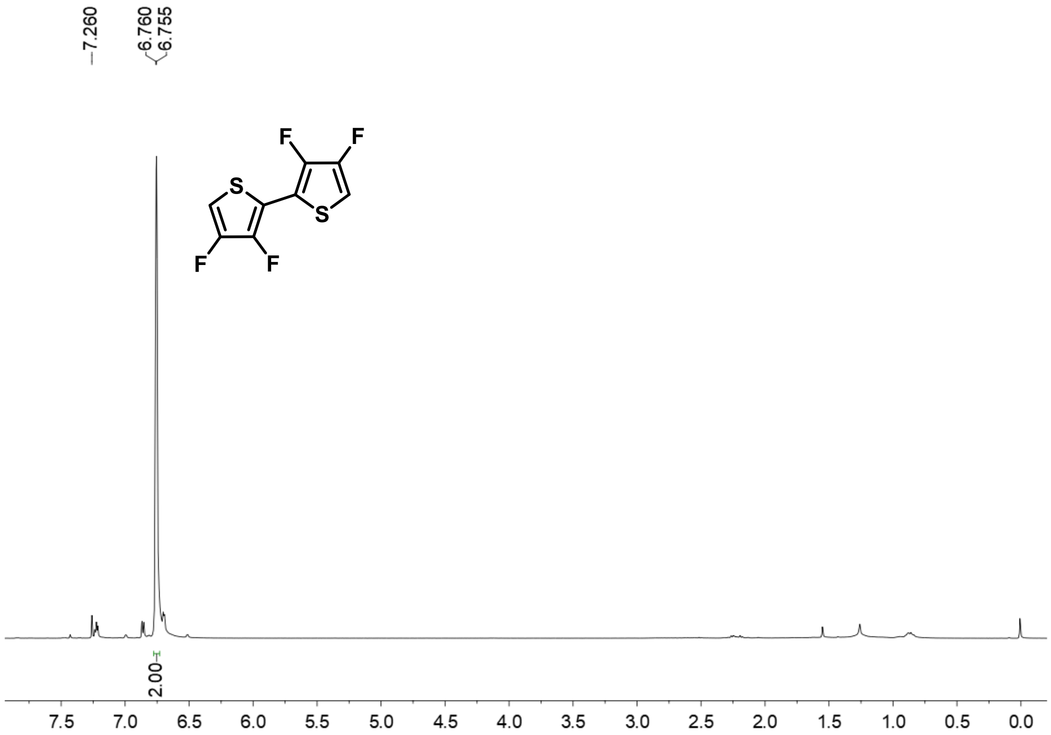

Supplementary Figure 65. ^{1}H NMR spectrum of compound **11**.

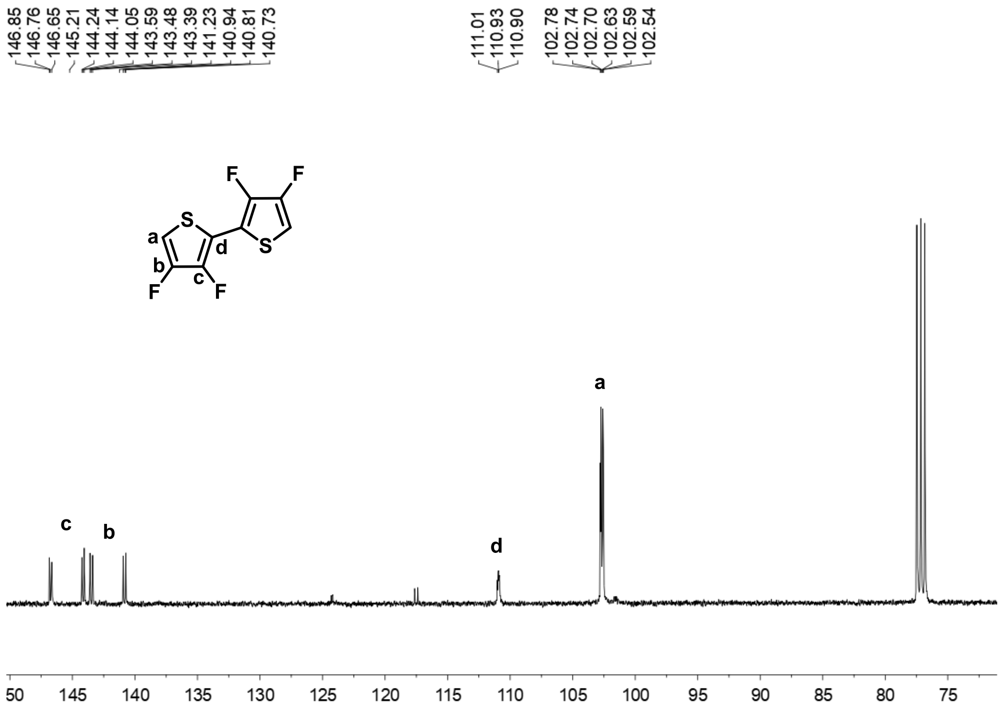

Supplementary Figure 66. ^{13}C NMR spectrum of compound **11**.

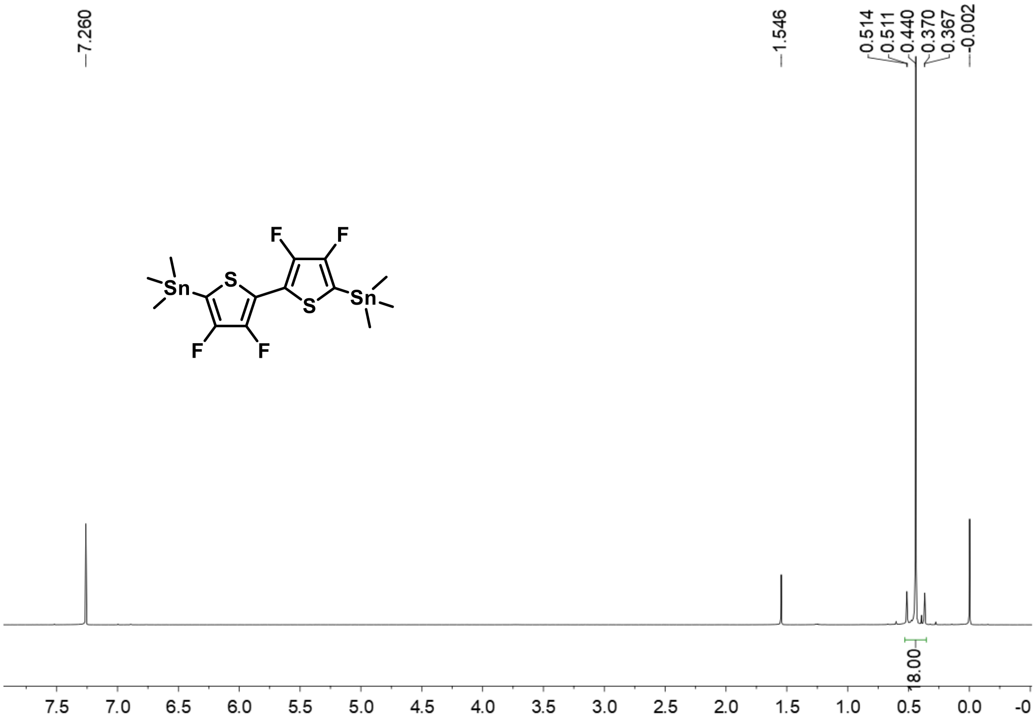

Supplementary Figure 67. ^{1}H NMR spectrum of compound **12**.

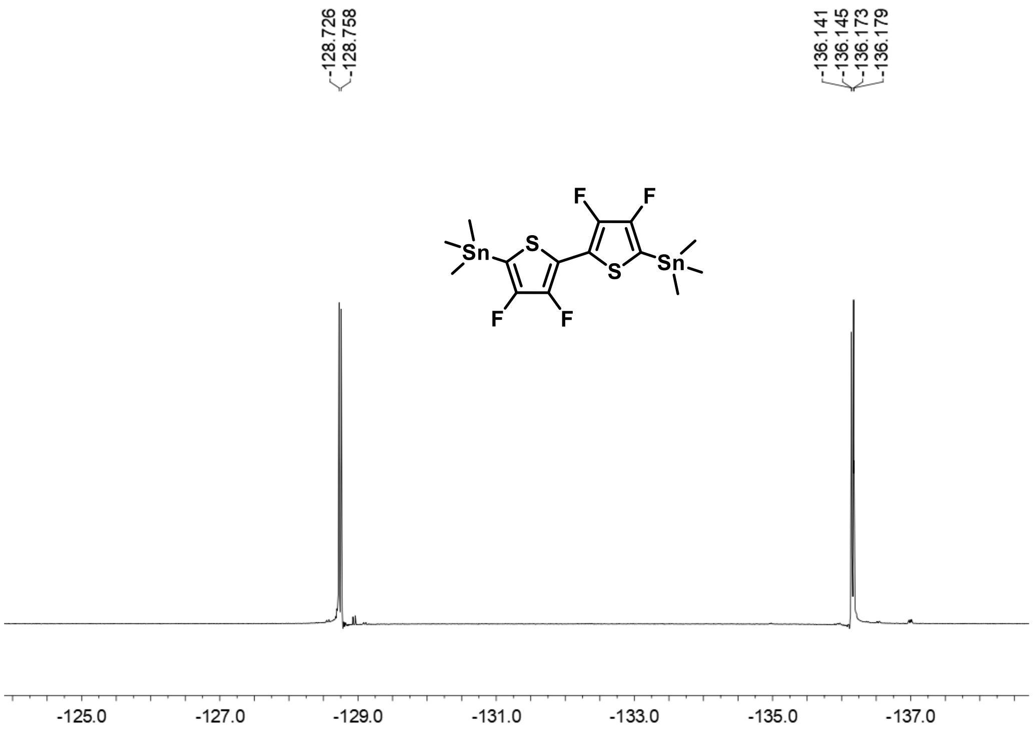

Supplementary Figure 68. ^{19}F NMR spectrum of compound **12**.

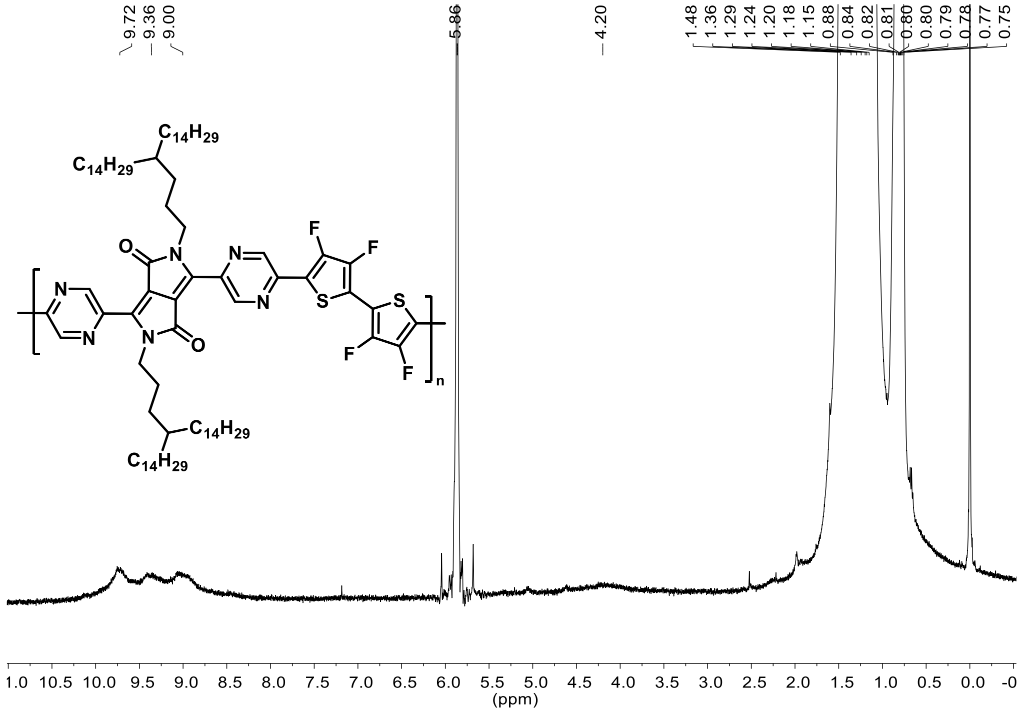

Supplementary Figure 69. ^{1}H NMR spectrum of polymer **P(PzDPP-4F2T)**.

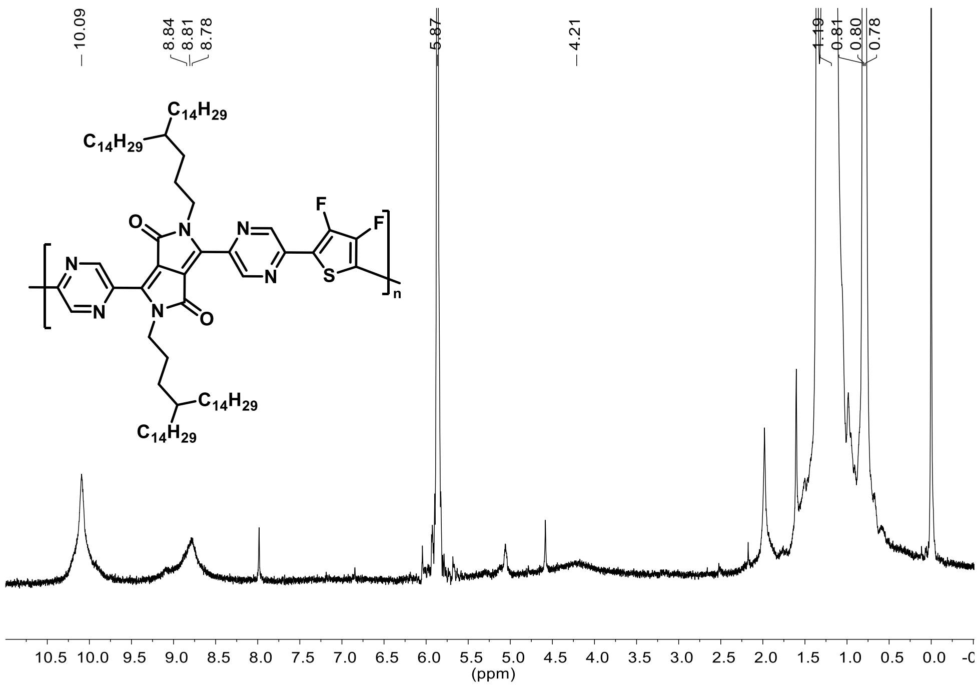

Supplementary Figure 70. ^{1}H NMR spectrum of polymer **P(PzDPP-2FT)**.

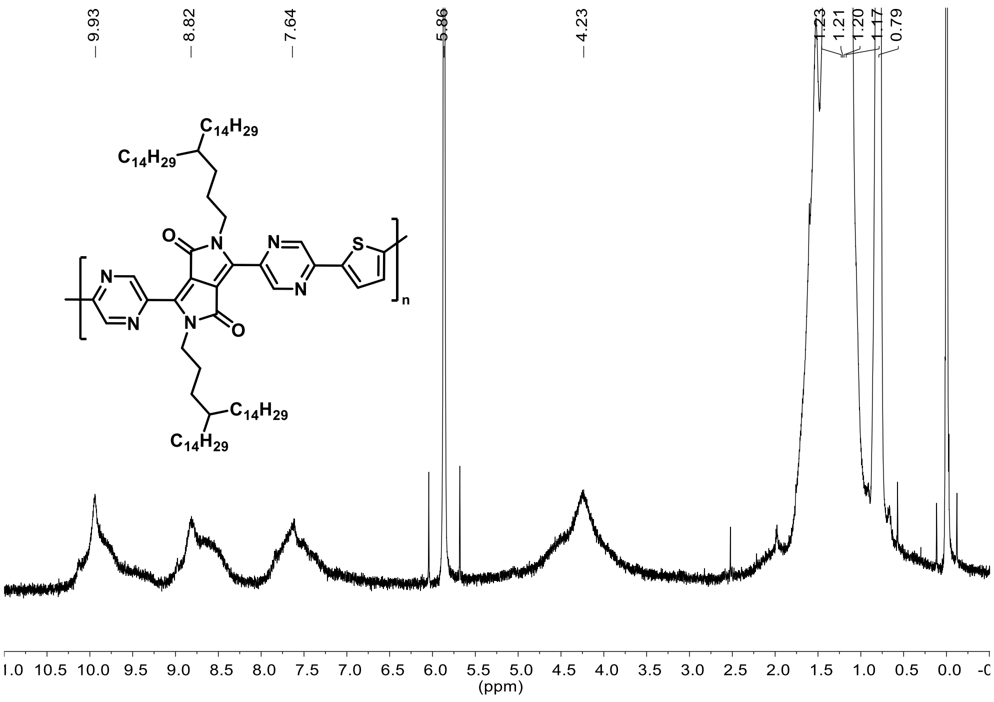

Supplementary Figure 71. ^{1}H NMR spectrum of polymer **P(PzDPP-T)**.

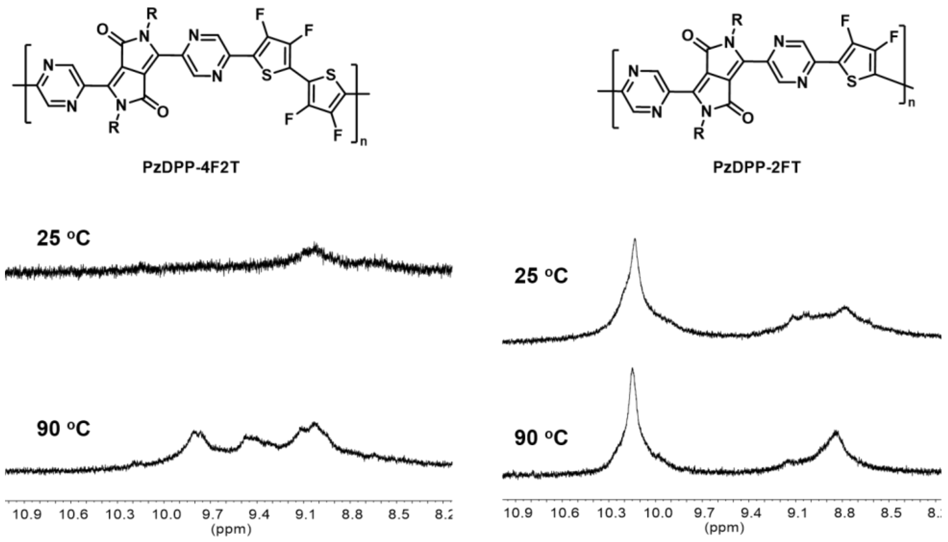

Supplementary Figure 72. ^{1}H NMR spectra of the aromatic signals of P(PzDPP-4F2T) and P(PzDPP-2FT) in $CDCl_2CDCl_2$ at 25 °C and 90 °C.

Because polymers are not single-component compounds and the relaxation times of aromatic hydrogen and aliphatic hydrogen are different, the integral ratio of aromatic hydrogens to aliphatic hydrogens usually deviates far from the normal value[38]. It is difficult to clearly characterize the structure of conjugated polymers by ^{1}H NMR spectra due to the entanglement and strong interaction between polymer chains. Recently, Adele Mucci *et al.* reported that the ^{1}H NMR spectra of conjugated polymers had a close relationship with their aggregation behavior in solution[39]. They observed significant changes of the aromatic signals in the ^{1}H NMR spectra of a P3HT derivative by adjusting the ratio of good solvent and poor solvent. The results demonstrated that the aggregation behavior of polymer in solution strongly affected the shape of the peaks in ^{1}H NMR. Compared with the P3HT derivatives, our rigid polymers are more strongly aggregated in solution to form different aggregates and exhibit more complicated ^{1}H NMR spectra.

The broad and multiple aromatic signals at 8.0-10.5 ppm in the ^{1}H NMR spectra of P(PzDPP-4F2T) are caused by the aggregation behavior in the solution. To further confirm that the multiple broad aromatic signals of P(PzDPP-4F2T) are caused by its different aggregates in solution, temperature-dependent ^{1}H NMR spectra of P(PzDPP-4F2T) are tested (Supplementary Fig. 72). Due to the strong π-π stacking and aggregation in the solution, P(PzDPP-4F2T) showed broad and unobvious signals in the aromatic region at 25 °C. When the temperature was raised up to 90 °C, the signals became sharp and obvious due to the disaggregation at high temperatures. P(PzDPP-2FT) showed broad and multiple signals in the aromatic region at 25 °C. The signals became

sharper, and the multiple peaks in the 25 °C spectrum emerged into one narrow peak when increasing the temperature from 25 °C to 90 °C, indicating the weaker chain interactions at high temperatures.

Recently, John R. Reynolds *et al.* also reported that the ^{1}H NMR spectra of conjugated polymers are closely related to the differences in polymer conformations[40]. In our work, the very small torsion barrier between the two thiophene units (φ_5) in P(PzDPP-4F2T) allows the polymer to adopt different conformations in solution. Therefore, it's reasonable that P(PzDPP-4F2T) shows multiple aromatic signals in its ^{1}H NMR spectrum. In contrast, P(PzDPP-2FT) shows steep torsional potentials at Pyrazine-DPP (φ_1) and Pyrazine-2FT (φ_2) angles, which makes it adopt a dominant conformation in solution and exhibit the expected two signals of similar intensity in the aromatic region.

Based on the above discussion, the ^{1}H NMR spectra of both P(PzDPP-2FT) and P(PzDPP-4F2T) exactly reflect that P(PzDPP-2FT) has a more planar and more shape-persistent backbone than P(PzDPP-4F2T).

Supplementary References:

1 Yang, J., Zhao, Z., Wang, S., Guo, Y. & Liu, Y. Insight into high-performance conjugated polymers for organic field-effect transistors. *Chem* **4**, 2748−2785 (2018).

2 Sun, H., Guo, X. & Facchetti, A. High-performance n-type polymer semiconductors: applications, recent development, and challenges. *Chem* **6**, 1310−1326 (2020).

3 Venkateshvaran, D. *et al.* Approaching disorder-free transport in high-mobility conjugated polymers. *Nature* **515**, 384−388 (2014).

4 Riplinger, C., Sandhoefer, B., Hansen, A. & Neese, F. Natural triple excitations in local coupled cluster calculations with pair natural orbitals. *J. Chem. Phys.* **139**, 134101 (2013).

5 Salzner, U. & Aydin, A. Improved prediction of properties of π-conjugated oligomers with range-separated hybrid density functionals. *J. Chem. Theory. Comput.* **7**, 2568−2583 (2011).

6 Che, Y. & Perepichka, D. F. Quantifying planarity in the design of organic electronic materials. *Angew. Chem. Int. Ed.* **60**, 1364−1373 (2021).

7 Lin, T.-J. & Lin, S.-T. Theoretical study on the torsional potential of alkyl, donor, and acceptor substituted bithiophene: the hidden role of non-covalent interaction and backbone conjugation. *Phys. Chem. Chem. Phys.* **17**, 4127−4136 (2015).

8 Jackson, N. E. *et al.* Controlling conformations of conjugated polymers and small molecules: the role of nonbonding interactions. *J. Am. Chem. Soc.* **135**, 10475−10483 (2013).

9 Contreras-García, J. *et al.* NCIPLOT: a program for plotting noncovalent interaction regions. *J. Chem. Theory. Comput.* **7**, 625−632 (2011).

10 Lu, T. & Chen, F. Multiwfn: a multifunctional wavefunction analyzer. *J. Comput. Chem.* **33**, 580−592 (2012).

11 Johnson, E. R. *et al.* Revealing non-covalent interactions. *J. Am. Chem. Soc.* **132**, 6498−6506 (2010).

12 Humphrey, W., Dalke, A. & Schulten, K. VMD: Visual molecular dynamics. *J. Mol. Graph.* **14**, 33−38 (1996).

13 Gaussian 16, Revision A.03, M. J. Frisch, G. W. Trucks, H. B. Schlegel, G. E. Scuseria, M. A. Robb, J. R. Cheeseman, G. Scalmani, V. Barone, B. Mennucci, G. A. Petersson, H. Nakatsuji, M. Caricato, X. Li, H. P. Hratchian, A. F. Izmaylov, J. Bloino, G. Zheng, J. L. Sonnenberg, M. Hada, M. Ehara, K. Toyota, R. Fukuda, J. Hasegawa, M. Ishida, T. Nakajima, Y. Honda, O. Kitao, H. Nakai, T. Vreven, J. A. Montgomery, Jr., J. E. Peralta, F. Ogliaro, M. Bearpark, J. J. Heyd, E. Brothers, K. N. Kudin, V. N. Staroverov, R. Kobayashi, J. Normand, K. Raghavachari, A. Rendell, J. C. Burant, S. S. Iyengar, J. Tomasi, M. Cossi, N. Rega, J. M. Millam, M. Klene, J. E. Knox, J. B. Cross, V. Bakken, C. Adamo, J. Jaramillo, R. Gomperts, R. E. Stratmann, O. Yazyev, A. J. Austin, R. Cammi, C. Pomelli, J. W. Ochterski, R. L. Martin, K. Morokuma, V. G. Zakrzewski, G. A. Voth, P. Salvador, J. J. Dannenberg, S. Dapprich, A. D. Daniels, O. Farkas, J. B. Foresman, J. V. Ortiz, J. Cioslowski, and D. J. Fox, Gaussian, Inc., Wallingford CT, 2016.

14 Matthews, J. R. *et al.* Scalable synthesis of fused thiophene-diketopyrrolopyrrole semiconducting polymers processed from nonchlorinated solvents into high performance thin film transistors. *Chem. Mater.* **25**, 782−789 (2013).

15 Lei, T., Dou, J.-H., Cao, X.-Y., Wang, J.-Y. & Pei, J. A BDOPV-based donor–acceptor polymer for high-performance n-type and oxygen-doped ambipolar field-effect transistors. *Adv. Mater.* **25**, 6589−6593 (2013).

16 Dou, J.-H. *et al.* Systematic investigation of side-chain branching position effect on electron carrier mobility in conjugated polymers. *Adv. Funct. Mater.* **24**, 6270−6278 (2014).

17 Kronemeijer, A. J. *et al.* Two-dimensional carrier distribution in top-gate polymer field-effect transistors: correlation between width of density of localized states and Urbach energy. *Adv. Mater.* **26**, 728−733 (2014).

18 Materials Studio v6.0.0 Accelrys Software Inc, S. D., CA, 2011.

19 Lefebvre, C. *et al.* Accurately extracting the signature of intermolecular interactions present in the NCI plot of the reduced density gradient versus electron density. *Phys. Chem. Chem. Phys.* **19**, 17928−17936 (2017).

20 Neese, F., Wennmohs, F., Becker, U. & Riplinger, C. The ORCA quantum chemistry program package. *J. Chem. Phys.* **152**, 224108 (2020).

21 Lu, Y. *et al.* Rigid coplanar polymers for stable n-type polymer thermoelectrics. *Angew. Chem. Int. Ed.* **58**, 11390−11394 (2019).

22 Russ, B., Glaudell, A., Urban, J. J., Chabinyc, M. L. & Segalman, R. A. Organic thermoelectric materials for energy harvesting and temperature control. *Nat. Rev. Mater.* **1**, 16050 (2016).

23 Yan, X. *et al.* Pyrazine-flanked diketopyrrolopyrrole (DPP): a new polymer building block for high-performance n-type organic thermoelectrics. *J. Am. Chem. Soc.* **141**, 20215−20221 (2019).

24 Shi, K. *et al.* Toward high performance n-type thermoelectric materials by rational modification of BDPPV backbones. *J. Am. Chem. Soc.* **137**, 6979−6982 (2015).

25 Wang, S. H. *et al.* Thermoelectric properties of solution-processed n-doped ladder-type conducting polymers. *Adv. Mater.* **28**, 10764−10771 (2016).

26 Naab, B. D. *et al.* Role of polymer structure on the conductivity of n-doped polymers. *Adv. Electron. Mater.* **2**, 1600004 (2016).

27 Ma, W. *et al.* Enhanced molecular packing of a conjugated polymer with high organic thermoelectric power factor. *ACS Appl. Mater. Inter.* **8**, 24737−24743 (2016).

28 Zhao, X. G. *et al.* High conductivity and electron-transfer validation in an n-type fluoride-anion-doped polymer for thermoelectrics in air. *Adv. Mater.* **29**, 1606928 (2017).

29 Wang, Y. *et al.* Naphthodithiophenediimide–benzobisthiadiazole-based polymers: versatile n-type materials for field-effect transistors and thermoelectric devices. *Macromolecules* **50**, 857−864 (2017).

30 Perry, E. E. *et al.* High conductivity in a nonplanar n-doped ambipolar semiconducting polymer. *Chem. Mater.* **29**, 9742−9750 (2017).

31 Kiefer, D. *et al.* Enhanced n-doping efficiency of a naphthalenediimide-based copolymer through polar side chains for organic thermoelectrics. *ACS Energy Lett.* **3**, 278−285 (2018).

32 Liu, J. *et al.* Enhancing molecular n-type doping of donor-acceptor copolymers by tailoring side chains. *Adv. Mater.* **30**, 1704630 (2018).
33 Liu, J. *et al.* N-type organic thermoelectrics of donor-acceptor copolymers: improved power factor by molecular tailoring of the density of states. *Adv. Mater.* **30**, 1804290 (2018).
34 Wang, S. H. *et al.* A chemically doped naphthalenediimide-bithiazole polymer for n-type organic thermoelectrics. *Adv. Mater.* **30**, 1801898 (2018).
35 Yang, C. Y. *et al.* Enhancing the n-type conductivity and thermoelectric performance of donor–acceptor copolymers through donor engineering. *Adv. Mater.* **30**, 1802850 (2018).
36 Nava, D. *et al.* Drastic improvement of air stability in an n-type doped naphthalene-diimide polymer by thionation. *ACS Appl. Energ. Mater.* **1**, 4626−4634 (2018).
37 Liu, J. *et al.* Overcoming coulomb interaction improves free-charge generation and thermoelectric properties for n-doped conjugated polymers. *ACS Energy Lett.* **4**, 1556−1564 (2019).
38 Robert M. Silverstein, Francis X. Webster, David J. Kiemle & Bryce, D. L. *Spectrometric identification of organic compounds*. 8th Edition edn, 126−190 (Wiley, 2014).
39 Parenti, F., Tassinari, F., Libertini, E., Lanzi, M. & Mucci, A. π-Stacking signature in NMR solution spectra of thiophene-based conjugated polymers. *ACS Omega* **2**, 5775−5784 (2017).
40 Lo, C. K. *et al.* Every atom counts: elucidating the fundamental impact of structural change in conjugated polymers for organic photovoltaics. *Chem. Mater.* **30**, 2995−3009 (2018).
